# Exploratory Analysis of *TP53* Mutations in Circulating Tumour DNA as Biomarkers of Treatment Response for Patients with Relapsed High-Grade Serous Ovarian Carcinoma: A Retrospective Study

**DOI:** 10.1371/journal.pmed.1002198

**Published:** 2016-12-20

**Authors:** Christine A. Parkinson, Davina Gale, Anna M. Piskorz, Heather Biggs, Charlotte Hodgkin, Helen Addley, Sue Freeman, Penelope Moyle, Evis Sala, Karen Sayal, Karen Hosking, Ioannis Gounaris, Mercedes Jimenez-Linan, Helena M. Earl, Wendi Qian, Nitzan Rosenfeld, James D. Brenton

**Affiliations:** 1 Cancer Research UK Cambridge Institute, University of Cambridge, Cambridge, United Kingdom; 2 Department of Oncology, Hutchison/MRC Research Centre, University of Cambridge, Cambridge, United Kingdom; 3 NIHR Cambridge Biomedical Research Centre, Cambridge, United Kingdom; 4 Cambridge University Hospitals NHS Foundation Trust, Cambridge, United Kingdom; 5 Cancer Research UK Major Centre–Cambridge, Cancer Research UK Cambridge Institute, Cambridge, United Kingdom; Washington University School of Medicine, UNITED STATES

## Abstract

**Background:**

Circulating tumour DNA (ctDNA) carrying tumour-specific sequence alterations may provide a minimally invasive means to dynamically assess tumour burden and response to treatment in cancer patients. Somatic *TP53* mutations are a defining feature of high-grade serous ovarian carcinoma (HGSOC). We tested whether these mutations could be used as personalised markers to monitor tumour burden and early changes as a predictor of response and time to progression (TTP).

**Methods and Findings:**

We performed a retrospective analysis of serial plasma samples collected during routine clinical visits from 40 patients with HGSOC undergoing heterogeneous standard of care treatment. Patient-specific *TP53* assays were developed for 31 unique mutations identified in formalin-fixed paraffin-embedded tumour DNA from these patients. These assays were used to quantify ctDNA in 318 plasma samples using microfluidic digital PCR. The *TP53* mutant allele fraction (TP53MAF) was compared to serum CA-125, the current gold-standard response marker for HGSOC in blood, as well as to disease volume on computed tomography scans by volumetric analysis. Changes after one cycle of treatment were compared with TTP.

The median TP53MAF prior to treatment in 51 relapsed treatment courses was 8% (interquartile range [IQR] 1.2%–22%) compared to 0.7% (IQR 0.3%–2.0%) for seven untreated newly diagnosed stage IIIC/IV patients. TP53MAF correlated with volumetric measurements (Pearson *r =* 0.59, *p <* 0.001), and this correlation improved when patients with ascites were excluded (*r =* 0.82). The ratio of TP53MAF to volume of disease was higher in relapsed patients (0.04% per cm^3^) than in untreated patients (0.0008% per cm^3^, *p =* 0.004). In nearly all relapsed patients with disease volume > 32 cm^3^, ctDNA was detected at ≥20 amplifiable copies per millilitre of plasma. In 49 treatment courses for relapsed disease, pre-treatment TP53MAF concentration, but not CA-125, was associated with TTP. Response to chemotherapy was seen earlier with ctDNA, with a median time to nadir of 37 d (IQR 28–54) compared with a median time to nadir of 84 d (IQR 42–116) for CA-125. In 32 relapsed treatment courses evaluable for response after one cycle of chemotherapy, a decrease in TP53MAF of >60% was an independent predictor of TTP in multivariable analysis (hazard ratio 0.22, 95% CI 0.07–0.67, *p =* 0.008). Conversely, a decrease in TP53MAF of ≤60% was associated with poor response and identified cases with TTP < 6 mo with 71% sensitivity (95% CI 42%–92%) and 88% specificity (95% CI 64%–99%). Specificity was improved when patients with recent drainage of ascites were excluded. Ascites drainage led to a reduction of TP53MAF concentration. The limitations of this study include retrospective design, small sample size, and heterogeneity of treatment within the cohort.

**Conclusions:**

In this retrospective study, we demonstrated that ctDNA is correlated with volume of disease at the start of treatment in women with HGSOC and that a decrease of ≤60% in TP53MAF after one cycle of chemotherapy was associated with shorter TTP. These results provide evidence that ctDNA has the potential to be a highly specific early molecular response marker in HGSOC and warrants further investigation in larger cohorts receiving uniform treatment.

## Abstract 2

James Brenton and colleagues reveal tumour burden and prognosis correlations from circulating DNA analysis in high grade serous ovarian carcinoma.

## Author Summary

**Why Was This Study Done?:**

- The standard clinical blood test for measuring response in women receiving chemotherapy for high-grade serous ovarian cancer (HGSOC) is the serum protein cancer antigen 125 (CA-125).
- CA-125 is sensitive but it lacks specificity for detection of ovarian cancer, and in response to chemotherapy, CA-125 level does not change rapidly enough to suggest change in treatment after one or two cycles if chemotherapy treatment is ineffective.
- Better tumour markers are required, and circulating tumour DNA (ctDNA) is a promising candidate.
- ctDNA is cell-free DNA derived from tumour cells that can be detected in the bloodstream; ctDNA can be used as a highly specific marker because it carries mutations unique to the cancer.

**What Did the Researchers Do and Find?:**

- HGSOC is an ideal cancer to test ctDNA as a biomarker because 99% of patients have a mutation in the *TP53* gene.
- We designed patient-specific *TP53* assays for a retrospective study of 40 patients with HGSOC, and these were used to quantify the amount of ctDNA in 318 plasma samples collected before, during, and after chemotherapy.
- We asked if ctDNA level was correlated with the amount of disease present before chemotherapy treatment measured using 3-D volume reconstruction from CT images taken as part of routine care.
- We also asked if the decrease in *TP53* ctDNA after one cycle of chemotherapy treatment could predict which patients would have progression of their cancer within six months.
- ctDNA level, but not CA-125 level, was strongly correlated with the total volume of disease.
- Patients whose ctDNA level exhibited a decrease of >60% after one cycle of chemotherapy had a significantly longer time to progression than those whose ctDNA level decreased by 60% or less.

**What Do These Findings Mean?:**

- *TP53* ctDNA has the potential to be a clinically useful blood test to assess prognosis and response to treatment in women with HGSOC.
- The response findings from this retrospective study should be confirmed in larger, prospective studies with uniform treatment. If these findings are confirmed, *TP53* ctDNA could be used in HGSOC clinical trials and routine practice to identify earlier whether treatment is effective.

## Introduction

The development of blood biomarkers that can be used for early detection of cancer or to measure tumour burden and response to treatment is a major goal of translational cancer research across all cancer types. Both tumour-derived proteins and DNA can be detected in circulating plasma and serum from cancer patients [1,2]. In epithelial ovarian cancer, particularly high-grade serous ovarian cancer (HGSOC), cancer antigen 125 (CA-125) is a serum glycoprotein biomarker used in standard clinical practice for the first assessment of pelvic masses [3] and for monitoring response to treatment [4,5]. However, CA-125 is limited by specificity, since it can also be expressed by normal tissues. Two large screening studies using CA-125 and ultrasound have failed to show an improvement in mortality on primary analysis [6,7]. CA-125 has heterogeneous intra- and inter-patient cellular expression and a long biological half-life in serum, resulting in utility for sequential clinical measurements to indicate the trend of treatment response, but not as a direct reflection of absolute tumour volume [8]. Whilst several studies have shown that pre-treatment CA-125 is prognostic and that early changes following chemotherapy are predictive [9,10], the positive and negative predictive values are not sufficient for use as a surrogate for radiological response or time to progression (TTP), or as the sole endpoint in registration trials. More recently, attention has turned to DNA-based biomarkers in blood as potentially superior measurements of response. In contrast to protein biomarkers, which typically are not specific to cancer cells, circulating tumour DNA (ctDNA) measures levels of mutations in plasma cell-free DNA and provides highly cancer-specific biomarkers. ctDNA fragments have a short half-life, and their levels have been shown in other cancer types to be related to tumour volume and response to treatment [11,12].

Although mutations in cell-free DNA have been studied for more than 20 years [13,14], recent improvements in assay sensitivity and in the ability to routinely identify patient-specific mutations have made it practical to accurately measure ctDNA levels in blood samples to monitor tumour response to treatment [12,15–18]. ctDNA may represent ~0.01%–90% of total circulating DNA and potentially offers greater specificity than protein biomarkers [19–21]. In initial studies performed on small numbers of patients, ctDNA has compared favourably with different serum tumour markers [16,20]. In a study of 18 metastatic colorectal cancer (CRC) patients following hepatic metastasectomy, Diehl and colleagues showed, using sensitive BEAMing assays, that ctDNA outperformed the serum marker carcinoembryonic antigen for the detection of microscopic disease [12]. Further studies in CRC have now been published correlating changes in ctDNA with response to chemotherapy [22]. Similarly, ctDNA outperformed the serum marker cancer antigen 15-3 for assessing tumour response to treatment in metastatic breast cancer [16].

HGSOC is an ideal cancer type in which to explore the clinical utility of ctDNA for response monitoring during treatment in comparison to a clinically accepted biomarker of response, as >99% of cases show mutations in *TP53* [23–25] and >90% of advanced HGSOC cases express the serum protein tumour marker CA-125 [26,27]. We and others have previously shown that *TP53* mutations can be detected in ctDNA from patients with advanced HGSOC and that, in a small number of patients studied, changes in ctDNA levels correlated with other clinical response measurements including CA-125 [21,28–30]. However, in HGSOC the relationship of ctDNA to tumour volume, dynamic ctDNA changes during chemotherapy, and the relationship of early changes to outcomes during chemotherapy have not been characterised.

The primary aim of this study was to define the distribution and dynamics of ctDNA in patients with recurrent HGSOC treated with standard of care chemotherapy, and to correlate ctDNA with the volume of disease. A secondary aim was to evaluate whether early change in ctDNA could predict TTP.

## Methods

### Ethics and Consent

Patients included in this report were enrolled in the prospective CTCR-OV04 clinical study, which collected blood and tissue samples for exploratory biomarker studies from patients treated at Addenbrooke’s Hospital, Cambridge University Hospitals NHS Foundation Trust. All patients provided written informed consent for participation in the study and for use of their donated tissue and blood specimens. The CTCR-OV04 study was approved by the Cambridgeshire Research Ethics Committee (reference 08/H0306/61).

### Study Design and Patients

**Fig 1:**
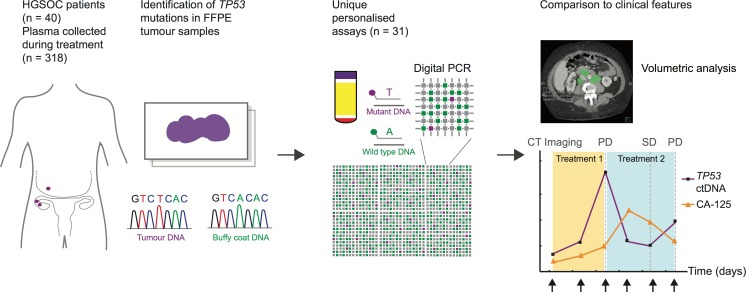
Schema of workflow for circulating tumour DNA analysis. ctDNA, circulating tumour DNA; FFPE, formalin-fixed paraffin-embedded; HGSOC, high-grade serous ovarian carcinoma; PD, progressive disease; SD, stable disease.

In order to quantify ctDNA levels, patient-specific *TP53* TaqMan assays were designed to target mutations identified in formalin-fixed paraffin-embedded (FFPE) tissue. Digital PCR was used to measure ctDNA levels in cell-free DNA from plasma samples collected during courses of chemotherapy, as shown in Fig 1.

This report has been written in accordance with REMARK and STROBE guidelines [31,32]. REMARK diagrams, the STROBE checklist, and a summary of the statistical analysis are found in S1 Checklist, S1 Text and S1 and S4 Figs. Patient selection criteria for this report were as follows: histological diagnosis of HGSOC of the ovary, primary peritoneum, or fallopian tube (hereafter HGSOC); ≥2 plasma samples from at least one course of chemotherapy including a pre-treatment sample collected before the start of the course; and *TP53* point mutation or short indel identified by sequencing of FFPE tumour DNA (S2 Fig).

The study was initiated as an exploratory retrospective analysis of samples collected as part of the CTCR-OV04 study protocol (see above). Further to initial findings, the cohort was expanded to 40 patients for whom missense mutations or short indels in *TP53* were identified by Sanger sequencing of tumour DNA. All patients were enrolled between 19 August 2009 and 13 June 2011. Patients were followed up during routine clinical practice. Follow-up was censored on 16 September 2015, with a median duration of 59 mo (range 43–70 mo). TTP for relapsed patients was defined as the interval from cycle 1 day 1 of chemotherapy to the date of progression measured by Response Evaluation Criteria in Solid Tumours (RECIST) 1.1 [33]. Patients with non-cancer-related/unknown cause of death were censored at the date of death and included in the TTP analysis. Clinical details, including CA-125, stage, residual disease after surgery, chemotherapy, and procedure dates, were abstracted from clinical records by research staff.

### *TP53* Mutation Identification

FFPE tissue blocks were cut as 8-μm sections and tumour-enriched regions were recovered by macrodissection based on regions marked on an adjacent haematoxylin-and-eosin-stained section by the study pathologist. DNA was extracted using the QIAamp DNA FFPE Tissue Kit (Qiagen) and quantified using a Qubit 2.0 Fluorometer (Invitrogen). Coding sequences of the *TP53* gene (exons 2–11) were PCR-amplified from FFPE DNA using primers and conditions as described previously [34] and sequenced using an ABI 3730 DNA Analyzer (Applied Biosystems), except that an additional forward instead of reverse primer (5′-CAGGTCTCCCCAAGGCGCAC-3′) was used for the sequencing of exon 7. Mutational analysis was performed using Mutation Surveyor software version 3.97 (SoftGenetics), and sequence data were aligned to *TP53* reference sequence NC_000017.10. In patients 127 and 200, mutations were identified in FFPE DNA by TAm-Seq (tagged-amplicon deep sequencing) using the 48.48 Access Array System (Fluidigm) and GAIIx Genome Analyzer (Illumina), as previously described [21].

### Plasma, Buffy Coat, and Serum Collection

Serial plasma samples were collected from patients, including at the appointment closest to the start of their chemotherapy course, which in most cases was on the first day of treatment. Peripheral blood samples were collected into EDTA tubes (Sarstedt) and centrifuged within 1 h of collection at 820*g* for 10 min to limit leukocyte lysis and degradation of cell-free DNA. Plasma aliquots of 1 ml were centrifuged in a benchtop microfuge at 14,000 rpm for 10 min, and the supernatant was transferred to sterile 1.5-ml tubes and stored at −80°C prior to extraction. Buffy coat samples were collected at the time of plasma collection, and stored at −80°C prior to DNA extraction using the QIAamp DNA Mini Kit (Qiagen). CA-125 assessments were carried out as part of routine clinical care in a clinically accredited laboratory. Where routine clinical CA-125 results were missing, CA-125 results were assessed using research serum samples taken at the same time point. For serum collections, 7.5-ml peripheral blood samples were collected into serum collection tubes (Sarstedt), gently inverted 5–10 times, and left upright at room temperature for 45–60 min to enable clot formation. Tubes were centrifuged at 3,000 rpm for 10 min, and 1-ml aliquots of serum were transferred to sterile 1.5-ml tubes and stored at −80°C.

### Extraction of Circulating DNA from Plasma

Circulating DNA was extracted from 0.85–2.8 ml (median 2.1 ml) of patient plasma using the QIAamp Circulating Nucleic Acid Kit (Qiagen), and a fraction of the extracted DNA was used for digital PCR analysis (5% of the extracted DNA for each panel). Carrier RNA was added to each sample prior to lysis, and eluant was passed twice through the QIAamp column to maximise yield. Control circulating DNA was extracted from a pool of plasma from five healthy female individuals (Sera Laboratories International).

### Assay Design and Validation

Dual-labelled patient-specific TaqMan assays were designed for mutated and wild-type *TP53* sequences, labelled with 6FAM, VIC, or HEX fluorophores (Applied Biosystems; Sigma-Aldrich). Sequences of all primers and probes are shown in S1 Table. Each assay was validated by digital PCR using matched FFPE tumour and buffy coat template DNA extracted from the same patient, and tumour DNA from individuals carrying non-matching *TP53* mutations. To test the performance of the assays, circulating DNA from a control mix of plasma samples from healthy volunteers was extracted 86 independent times, and *TP53* alleles (both wild-type and mutant alleles) measured by digital PCR a total of 141 times, using the 31 assays designed plus an additional similar assay designed for a patient who was excluded from this cohort (S1 and S2 Figs). Values of wild-type *TP53* concentrations fit a normal distribution, and 95% of values were within 1.5-fold of the mean value (S2 Fig). Each measurement included 765 independent real-time PCR amplifications. Of the 141 measurements (107,865 reactions in total), 19 false-positive amplifications of mutant alleles in the control samples were observed (0.018%), 13 of which were obtained in two of the 32 assays (for mutations g.12458G>A [p.R175H] and g.12460T>A [p.C176S]).

### Digital PCR

Digital PCR was performed using the Biomark microfluidic system (Fluidigm), as previously described [35]. Standard operating procedures were followed, and all assay setup and liquid handling were performed in a HEPA/UV sterilising PCR workstation (Ultra-Violet Products) in a PCR-free environment to prevent PCR contamination. Data analysis was performed using Digital PCR Analysis software version 3.02 (Fluidigm) and Matlab. For each assay, a threshold for positive amplification was determined by manual inspection of the PCR amplification curves in the patient and control circulating DNA samples, and this threshold was used to determine the number of observed amplifications. A Poisson correction was used to convert the number of observed amplifications to estimated targets assuming independent segregation of the DNA molecules into the multiple digital PCR reaction chambers. The measurement by microfluidic digital PCR was corrected for the relative fraction of the extracted DNA loaded on the microfluidic array (including correction for “dead volume” lost on the array) and was normalised to units of amplifiable copies per millilitre of plasma (AC/ml). In the procedure, 3.5 μl of the total volume of 70 μl of eluted DNA was loaded for each digital PCR panel, and with correction for dead volume (~54% lost), the total DNA assayed was equivalent to a median of 0.05 ml of plasma per sample. Correspondingly, we set a cutoff for ctDNA detection at 20 AC/ml.

Total circulating cell-free DNA was measured as the *TP53* total allele count (TP53TAC), the sum of estimated targets of mutated and wild-type copies of *TP53* amplified by the assay primers. The level of mutated *TP53* ctDNA was quantified in two ways: by the number of mutated *TP53* amplifiable copies (*TP53* mutant allele count [TP53MAC]), defined as the number of single-stranded fragments of DNA amplified by the assay primers and containing the mutation of interest, and by the *TP53* mutant allele fraction (TP53MAF), defined as TP53MAC divided by TP53TAC.

### CA-125

Serum CA-125 level was routinely monitored using a two-site sandwich immunoassay on a Siemens Centaur XP auto-analyser (upper limit of normal ≤ 30 IU/ml). CA-125 response was assessed in accordance with Gynecologic Cancer InterGroup criteria [36].

### Computed Tomography Imaging

Patients underwent computed tomography (CT) imaging as part of standard care. A subset of patients had PET/CT imaging data available. All scans were retrospectively evaluated according to RECIST 1.1 by consultant radiologists subspecialised in gynaecological oncology imaging. The measurement of tumour volume was performed by consultant radiologists who were blinded to the ctDNA variables. CT images were uploaded onto a dedicated workstation and retrospectively reviewed with syngo.via multi-modality software (Siemens). A region of interest was manually placed around each visible lesion (e.g., peritoneal deposits, subcapsular disease, omental disease, ovarian masses, nodal disease), and the total volume of disease was calculated. Cases with ill-defined stranding were assessed as non-measurable disease. The presence or absence of ascites on CT was recorded.

### Statistics

Pre-specified analyses were determined after sample collection but before statistical analysis was performed. Additional exploratory analyses were carried out based on the results obtained (S1 Text). Baseline characteristics were summarised using the standard descriptive statistics: mean ± standard deviation or median with interquartile range (IQR) for continuous variables and percentage for categorical variables. Comparisons of the ratio of TP53MAF to volume of disease and CA-125 at baseline between untreated and relapsed patients were assessed with the Wilcoxon rank-sum test). Where two pre-treatment ctDNA samples were available, the sample closest to the date of volumetric CT was chosen for volumetric correlation. For all other analyses, the sample closest to treatment start date was selected.

Correlations between TP53MAF and CA-125 values and tumour volumes were analysed by Pearson rank correlation using log10 values. For log correlation calculations, TP53MAF and TP53MAC values of zero were adjusted by the addition of 0.001 times the value of the lowest value in the series.

The Cox regression model was applied to investigate the value of TP53MAF pre-treatment, change after cycle 1, and change after cycle 2 in predicting TTP. Multivariable analysis was adjusted for the following factors: age (continuous), ECOG (Eastern Cooperative Oncology Group) performance status (PS) (continuous), platinum sensitivity (yes or no), number of previous lines of chemotherapy (2 versus ≥3), tumour volume (continuous), ascites (absent or present), and TP53TAC. Three courses from three separate patients had missing PS, and PS was imputed as the mean PS value for all courses in the model. Total cell-free DNA level (TP53TAC) was included in the multivariable model because it has been reported as a possible independent prognostic marker in ovarian and other cancers [37,38].

In analyses where treatment courses with recent ascitic or pleural drainage were excluded, we defined “recent” as within 28 d of the baseline ctDNA sample, or between baseline and the subsequent cycle of interest (cycle 2 or 3).

We defined ctDNA as being evaluable for analysis of response if baseline TP53MAC was ≥40 AC/ml (double the lower limit of detection of ≥20 AC/ml).

The optimum cut-points for determining 6-mo TTP were identified using receiver operating characteristic (ROC) curves. The standard log-rank test was applied for the comparisons on TTP. Statistical analyses were carried out using SAS version 9.4 and R [39]; confidence intervals for sensitivity and specificity were calculated using MedCalc (http://www.medcalc.org).

## Results

### Patients, Samples, and Treatments

A total of 318 plasma samples were collected from 40 patients with HGSOC. S1 Fig shows the REMARK diagram for selection of patients; Table 1 shows the summary clinical features for the 40 patients (see also S2 Table for demographic information by patient and S3 Table for further details of plasma samples). In all, 261 samples were collected during treatment of relapsed disease, and included 54 courses of chemotherapy for 32 individual patients (including multiple lines of treatment per patient). A further 57 samples were collected during first-line treatment with chemotherapy from 12 patients, including four patients who had samples taken during first-line treatment and again at relapse. The ECOG PS of patients was 0–2 for all courses, where known. The median number of courses of treatment per patient with ctDNA analysis was 2 (IQR 1–2), and the median number of cycles with ctDNA analysis per course of treatment was 5 (IQR 3–6) (S1 and S2 Data).

**Table 1:** Summary statistics.

Feature	Value
**Number of patients**	40
**Age at diagnosis, median (range) (years)**	63 (38–85)
**Cancer type (number of patients)**	
Ovarian	32
Primary peritoneal	5
Fallopian tube	3
**Stage at diagnosis (number of patients)**	
I	3
II	0
III	27
IV	10
**Newly diagnosed untreated (*n***_**courses**_ **= 7)**	
TP53MAF, median (IQR)	0.7% (0.3%–2.0%)
CA-125, median (IQR) (IU/ml)	964 (488–2,909)
Volume, median (IQR) (cm^3^)	418 (172–1,770)
**Relapsed disease (*n***_**courses**_ **= 51)**	
TP53MAF, median (IQR)	8% (1.2%–22%)
CA-125, median (IQR) (IU/ml)	422 (205–1,108)
Volume, median (IQR) (cm^3^)	93.5 (37.0–176.0)

IQR, interquartile range; TP53MAF, *TP53* mutant allele fraction.

### *TP53* Mutation Identification and Assay Validation

Patient-specific TaqMan assays were designed for the 40 patients, who had in total 31 unique somatic *TP53* mutations (Figs 1 and S1; S1 Table). The most common mutations were g.12458G>A (p.R175H) and g.13744G>A (p.R273H), each identified in four different patients. Dual-labelled assays were designed to measure copies of wild-type and mutated *TP53* alleles using digital PCR. The length of the amplified regions was limited to effectively amplify fragmented cell-free circulating DNA (median 84 base pairs, range 58–177; S1 Table). The false-positive rates for the ctDNA assays were estimated at one mutated allele per 3,010 wild-type alleles (<0.033%, at 95% confidence) for 29 assays, and one mutated allele per 621 wild-type alleles (<0.16%, at 95% confidence) for assays for g.12458G>A (p.R175H) and g.12460T>A (p.C176S).

### Mutated *TP53* Circulating Tumour DNA Is Frequently Detected Pre-treatment in Patients with High-Grade Serous Ovarian Carcinoma

For the 54 courses of chemotherapy at relapse, pre-treatment samples were available for 51/54 courses from 32 relapsed patients (S4 Fig). Digital PCR was used to measure both total and fractional concentration of ctDNA (TP53MAC and TP53MAF; see Methods). Mutated *TP53* alleles in plasma were detected at TP53MAC ≥ 20 AC/ml pre-treatment in 42/51 (82%) of courses from relapsed patients.

Mutated *TP53* alleles in plasma were detected at TP53MAC ≥ 20 AC/ml pre-treatment in 6/7 (86%) of newly diagnosed stage IIIC/IV patients. CA-125 was above the institutional upper limit of normal in 100% of relapsed and untreated patients.

Median TP53MAF pre-treatment was 8.0% in patients with recurrent disease, 0.7% in patients with newly diagnosed stage IIIC/IV disease, and 0.2% in four patients after primary surgery (see S4 Table for full description of statistics). TP53MAF correlated with CA-125 (*r =* 0.49, *p <* 0.001). (S5 Table shows all correlations.)

### Circulating Tumour DNA Detection and Volume of Disease

**Fig 2:**
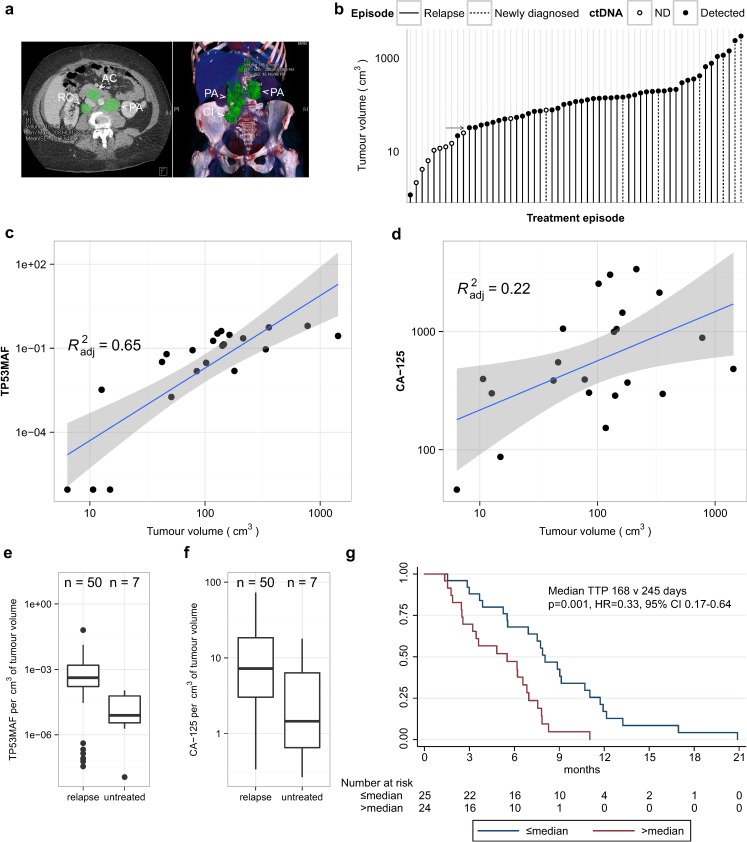
Comparison of *TP53* mutant allele fraction to tumour volume. (A) Example of volumetric analysis of high-grade serous ovarian cancer with relapsed disease in abdominal lymph nodes. Left panel shows cross-sectional view. Right panel shows 3-D reconstruction to show disease volume. Green shading indicates regions of interest for volume measurements. Lymph node masses are indicated by arrowheads and labelled as follows: AC, aorto-caval; CI, common iliac; PA, para-aortic; RC, retro-caval. (B) Ranked total volume of tumour at start of treatment course. Filled circles indicate cases with TP53MAC ≥ 20 AC/ml. Arrow indicates tumour volume of 32 cm^3^. (C and D) Linear regression analysis of TP53MAF and CA-125 with tumour volume in 22 relapsed events without ascites. Grey shading shows 95% confidence intervals. (E and F) Comparison of TP53MAF and CA-125 values adjusted for tumour volume between relapsed and newly diagnosed patients before treatment. (G) Time to progression analysis for relapsed patients with greater or less than the median pre-treatment TP53MAF. ctDNA, circulating tumour DNA; HR, hazard ratio; ND, not detected; TP53MAF, *TP53* mutant allele fraction.

We performed volumetric analysis of CT imaging performed prior to treatment for 51 relapsed courses (32 individual patients) and for seven newly diagnosed patients with stage IIIC/IV HGSOC (Fig 2A). One out of 51 relapsed treatment courses was excluded because CT imaging had non-measurable disease. Fig 2B shows the distribution of tumour volume across 57 treatment courses. All relapsed courses with tumour volume > 32 cm^3^ had ctDNA detected at TP53MAC ≥ 20 AC/ml except for one patient who had a TP53MAC of 15 AC/ml and disease volume of 50 cm^3^. All patients with tumour volume < 20 cm^3^ had TP53MAC < 20 AC/ml except for one patient with detected ctDNA with 1 cm^3^ of disease in the presence of large volume ascites (see below).

We next considered the possibility that the presence of ascites or pleural effusions could impact the accuracy of correlations between ctDNA and measured tumour volume because volumetric CT measurements were made only for solid disease, and the presence of ascites frequently alters CA-125 levels in clinical practice. We correlated levels of ctDNA with volume of disease in relapsed courses with and without ascites (S6 Table) in a subset of patients with closely matched dates of CT scans and ctDNA, and without drainage of ascites between these two time points. Of 50 relapsed pre-treatment ctDNA samples with matched volumetric CT data, ten were excluded because of a >14-d interval between CT imaging and ctDNA sample collection, and five because of a pleural or ascitic drain performed between the CT scan and plasma collection (S3 Data).

For the 35 remaining courses, TP53MAF showed a positive correlation with volume (Pearson *r =* 0.59, *p* < 0.001; S6 Table). Of these, 13/35 CT images showed the presence of ascites. When cases with ascites were excluded from analysis, the correlation of TP53MAF with volume increased (Pearson *r* = 0.82, *p* < 0.001; S6 Table), indicating that ctDNA in ascites may contribute to blood ctDNA levels. CA-125 was moderately correlated with tumour volume in all 35 cases and in those without ascites (Pearson *r* = 0.52, *p* = 0.001, and *r* = 0.51, *p* = 0.016, respectively; S6 Table). The linear regressions for TP53MAF and CA-125 in cases without ascites are shown in Fig 2C and 2D.

Median TP53MAF/volume in the patients without ascites was 0.08% per cm^3^ (IQR 0.02%–0.13% per cm^3^). TP53MAC showed lower correlation with tumour volume (S6 Table), and the median value of TP53MAC/volume was 6.0 AC/ml per cm^3^ (S3 Data).

### Relationship of *TP53* Mutant Allele Fraction to Volume in Recurrent Compared with Untreated Disease

The factors determining ctDNA blood levels are not well understood. Comparison of pre-treatment TP53MAF/volume values between relapsed and untreated patients showed a significant difference (*p =* 0.004, Wilcoxon rank-sum test; Fig 2E). CA-125/volume was not significantly different between these groups (*p =* 0.063; Wilcoxon rank-sum test; Fig 2F). The median TP53MAF/volume was 0.04% per cm^3^ in 50 relapsed courses compared with 0.0008% per cm^3^ in seven newly diagnosed untreated patients.

### Pre-treatment *TP53* Mutant Allele Fraction Is Associated with Time to Progression in Relapsed Patients

We next asked whether ctDNA measured prior to chemotherapy was associated with progression in patients with recurrent disease. We compared TP53MAF, CA-125, and total cell-free DNA (TP53TAC) to TTP estimates. Of the 50 relapse events with measurable disease, one treatment course was excluded because ctDNA was measured >14 d before start of treatment (S4 Fig). The median follow-up was 58 mo (range 43–70 mo), with all patients progressing during the follow-up period.

In univariable analysis, TP53MAF, CA-125, total cell-free DNA (TP53TAC), age, platinum sensitivity, the number of lines of chemotherapy, and volume of disease were all significant predictors of TTP (Table 2). When adjusted using the Cox proportional hazards model in multivariable analysis, only TP53MAF (hazard ratio [HR] 1.03, 95% CI 1.01–1.06, *p =* 0.019) and platinum sensitivity (HR 0.43, 95% CI 0.19–0.99, *p =* 0.048) remained significant. TTP was significantly longer for treatment courses with pre-treatment levels of TP53MAF below the median level than for treatment courses with TP53MAF above the median (*p =* 0.001 by log-rank test; Fig 2G). Pre-treatment TP53MAC was also tested as a continuous variable and was a significant predictor of TTP in univariable but not multivariable analysis (S10 Table).

**Table 2:** Univariable and multivariable analysis of pre-treatment values as a predictor of time to progression.

Variable (Units), *n*_courses_ = 49	Univariable			Multivariable		
	HR	CI	*p-*Value	HR	CI	*p-*Value
TP53MAF (percent)	1.04	1.02–1.06	<0.001***	1.03	1.01–1.06	0.019*
CA-125 (10^2^ IU/ml)	1.02	1.00–1.05	0.078	1.01	0.98–1.04	0.567
TP53TAC (10^3^ copies/ml)	1.03	1.01–1.04	0.009**	1.02	1.00–1.05	0.079
Age (years)	0.96	0.93–1.00	0.030*	0.96	0.92–1.01	0.089
Performance status (0–2)	0.72	0.44–1.18	0.192	0.64	0.36–1.17	0.146
Platinum sensitive (yes/no)	0.35	0.18–0.68	0.002**	0.43	0.19–0.99	0.048*
Number of lines chemotherapy (2 lines/≥3 lines)	0.43	0.22–0.83	0.013	0.75	0.35–1.64	0.478
Volume of disease (10 cm^3^)	1.02	1.01–1.03	0.002**	1.00	0.98–1.02	0.786
Ascites (no/yes)	0.95	0.53–1.71	0.858	0.82	0.42–1.58	0.548

For variables with HR > 1, an increase in the value is associated with a higher risk or number of events, and a decreased TTP. For binary variables, the HR listed is for the first option, with the second option being HR = 1 (if the HR listed is <1 then the first option is associated with lower risk and longer TTP).

* *p* < 0.05

** *p* < 0.01

*** *p* < 0.001.

CI, confidence interval; HR, hazard ratio; TP53MAF, *TP53* mutant allele fraction; TP53TAC, *TP53* total allele count; TTP, time to progression.

### Response Kinetics and Nadir of *TP53* Mutant Allele Fraction during Chemotherapy

**Fig 3:**
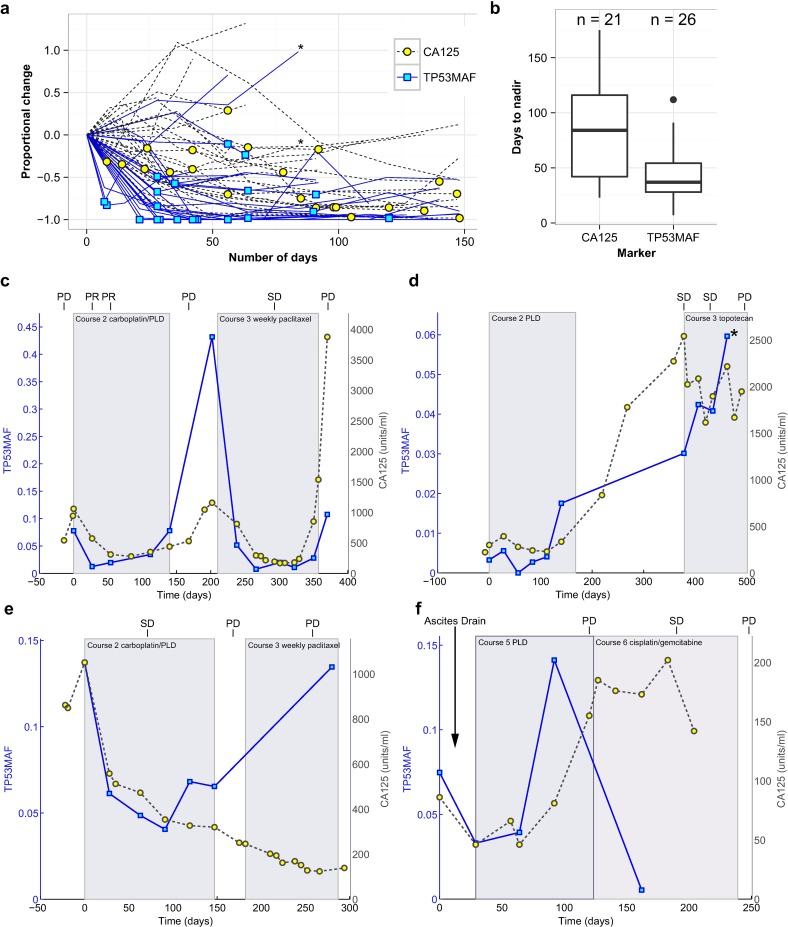
Circulating tumour DNA and CA-125 kinetics during chemotherapy. (A) TP53MAF and CA-125 kinetics from start of treatment, normalised to the pre-treatment levels. Asterisk denotes one treatment course where the patient developed new brain metastases. Yellow circles and blue boxes indicate nadir points. (B) Box plots show time to nadir following start of chemotherapy for CA-125 and TP53MAF. (C–F) Illustrative cases of TP53MAF and CA-125 kinetics. (C) Faster time to nadir and greater dynamic range of TP53MAF compared with CA-125. (D) Discrepant TP53MAF and CA-125 kinetics. This patient commenced on third-line chemotherapy and had a minor response on CT (stable disease by RECIST). CA-125 fell slightly whilst TP53MAF increased. After cycle 4, the patient developed new headaches, and a CT scan showed new brain metastases (marked by asterisk). (E) Discrepant TP53MAF and CA-125 kinetics. This patient commenced third-line chemotherapy, and the TP53MAF and CA-125 values diverged. CT scan showed progressive disease, in keeping with rise of TP53MAF. (F) The effect of ascitic drainage on plasma TP53MAF levels. This patient had an ascitic drain (at time = 4 d) before starting chemotherapy, with a ctDNA sample taken before (time = 0 d) and after (time = 29 d) the ascitic drain. Following drainage of 8 l of ascites, and before start of any further treatment, TP53MAF fell from 7.5% to 3.3%. CA-125 decreased from 86 IU/ml to 46 IU/ml. This patient had small-volume (1 cm^3^) solid disease and large-volume ascites. CA-125, cancer antigen 125; ctDNA, circulating tumour DNA; PD, progressive disease; PLD, pegylated liposomal doxorubicin; PR, partial response; SD, stable disease; TP53MAF, *TP53* mutant allele fraction.

We next analysed the kinetics of TP53MAF during treatment by measuring the time to achieve a nadir value following chemotherapy cycles. Only a subset of chemotherapy courses had consecutive plasma samples at each cycle and were assessable for nadir (see S7 Table for nadir assessment criteria). Of these, 26/27 courses reached a nadir for TP53MAF, compared with 21 for CA-125 (Fig 3A). The only course without a decrease in TP53MAF occurred in a patient who developed new brain metastases (patient 57). The median time to nadir was 37 d (IQR 28–54) for TP53MAF and 84 d (IQR 42–116) for CA-125. The median decrease at nadir was 98% for TP53MAF and 55% for CA-125 (Fig 3B).

We observed a more rapid decrease and greater dynamic range of TP53MAF measurements compared with CA-125 (Fig 3C). In most cases, TP53MAF and CA-125 trends with sequential treatment cycles were similar. In two cases, however, we observed discrepant kinetics, with rising TP53MAF and decreasing CA-125. In both cases, CT scans confirmed progressive disease, including in patient 57, who had progressive brain metastases (Fig 3D and 3E). The effect of ascitic drainage on plasma TP53MAF level is shown in Fig 3F. Drainage of ascites resulted in a decrease in TP53MAF, demonstrating that drainage may introduce rapid changes in plasma ctDNA level, potentially confounding comparison to response measures. The TP53MAF and CA-125 plots for all patients in the study are available in S3 Fig.

### Change in *TP53* Mutant Allele Fraction after One Cycle of Treatment Predicts Time to Progression

Early prediction of response or resistance to chemotherapy could have important implications for the clinical management of relapsed HGSOC patients. We therefore examined whether a decrease in TP53MAF after one cycle of chemotherapy could predict TTP, and compared this with CA-125. There were 32 courses of chemotherapy for relapsed patients that had matched samples at cycle 1 and cycle 2 and were evaluable for response (see S4 Fig for REMARK diagram). Of these, 22 were treated with platinum-based chemotherapy and ten with non-platinum chemotherapy. The median number of days from start of chemotherapy (cycle 1) to collection of plasma sample pre-cycle 2 was 28 d (IQR 21–28). The median TP53MAF decrease from pre-treatment to the pre-cycle-2 sample was 74% (IQR 55%–89%); median CA-125 decrease was 18% (IQR -46%-12%), and the median TTP was 189 d.

**Fig 4:**
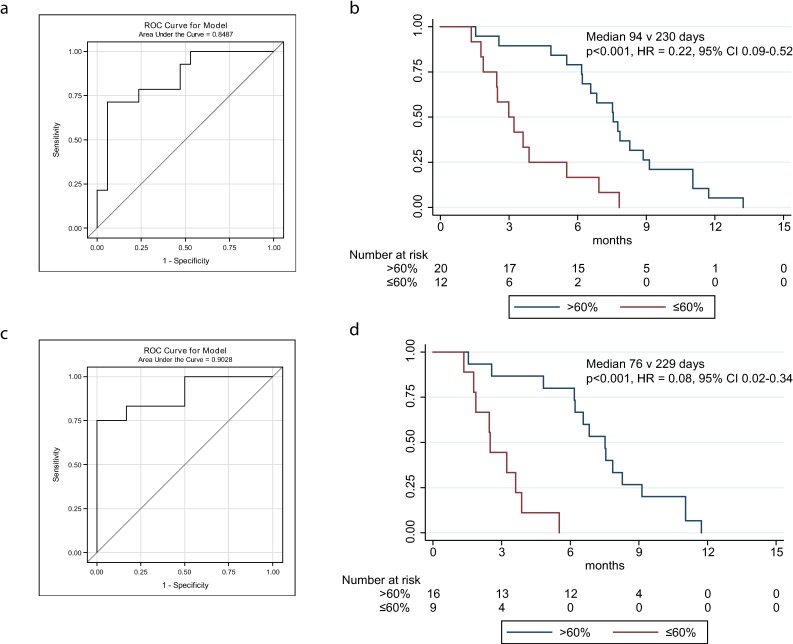
ROC curves and Kaplan-Meier plots for change in circulating tumour DNA after one cycle of chemotherapy, including and excluding courses with recent ascitic drains. (A) ROC plot identifies 60% decrease in TP53MAF as the most accurate threshold for predicting 6-mo TTP in all patients. (B) Kaplan-Meier curve showing TTP for patients with decrease of ≤60% or >60% after one cycle of chemotherapy. (C) ROC plot identifies a 60% decrease in TP53MAF as the most accurate threshold for predicting 6-mo TTP in patients without ascitic drains. (D) Kaplan-Meier curve for TP53MAF decrease after one cycle of chemotherapy to predict 6-mo progression-free survival in patients without ascitic drains. ctDNA, circulating tumour DNA; HR, hazard ratio; ROC, receiver operating characteristic; TP53MAF, *TP53* mutant allele fraction; TTP, time to progression.

To determine the optimal cut-point for predicting TTP from a decrease in TP53MAF, a ROC plot was generated, and 6-mo TTP was selected as a clinically significant endpoint. The ROC curve identified a 60% decrease in TP53MAF as the optimal cut-point for sensitivity and specificity (Fig 4A; S8 Table). This threshold was used in subsequent analyses. Median TTP was 94 d versus 230 d for a decrease of ≤60% and >60% in TP53MAF, respectively, with an HR of 0.22 (95% CI 0.09–0.52, *p* <0.001; Fig 4B).

In univariable analysis, volume of disease and a decrease in TP53MAF of >60% from pre-treatment to cycle 2 were significant predictors of TTP. In multivariable analysis, a TP53MAF decrease of >60% remained a significant predictive factor for 6-mo TTP (HR 0.22, 95% CI 0.07–0.67, *p =* 0.008; Table 3). CA-125 decrease was not significant.

**Table 3:** Univariable and multivariable analysis of 60% decrease in *TP53* mutant allele fraction after one cycle of chemotherapy as a predictor of time to progression.

Variable (Units), *n*_courses_ = 32	Univariable			Multivariable		
	HR	CI	*p-*Value	HR	CI	*p-*Value
TP53MAF decrease > 60% from C1 to C2 (yes/no)	0.22	0.09–0.52	<0.001***	0.22	0.07–0.67	0.008**
CA-125 decrease > 50% from C1 to C2 (yes/no)	0.58	0.23–1.43	0.234	0.86	0.28–2.71	0.802
Age (years)	1.00	0.95–1.04	0.841	0.97	0.91–1.03	0.276
Performance status (0–2)	0.78	0.35–1.76	0.549	0.74	0.29–1.87	0.522
Platinum sensitive (yes/no)	0.49	0.23–1.02	0.057	0.65	0.25–1.66	0.365
Number of lines chemotherapy (2 lines/≥3 lines)	0.53	0.24–1.16	0.114	0.92	0.30–2.81	0.888
Volume of disease (10 cm^3^)	1.02	1.00–1.03	0.031*	1.00	0.98–1.02	0.999
Ascites (no/yes)	1.36	0.66–2.82	0.409	1.76	0.75–4.16	0.197

For variables with HR > 1, an increase in the value is associated with a higher risk or number of events, and a decreased TTP. For binary variables, the HR listed is for the first option, with the second option being HR = 1 (if the HR listed is <1 then the first option is associated with lower risk and longer TTP).

* *p* < 0.05

** *p* < 0.01

*** *p* < 0.001.

C1, cycle 1; C2, cycle 2; CI, confidence interval; HR, hazard ratio; TP53MAF, *TP53* mutant allele fraction; TTP, time to progression.

TP53MAF decrease was also significant as a continuous variable in multivariable analysis (S9 Table). TP53MAC decrease after one cycle was also tested as a continuous variable and was not a significant predictor of TTP (S10 Table). Together with our observation that pre-treatment TP53MAC was not a significant predictor of TTP, these results indicate that TP53MAF was the most informative ctDNA parameter.

As recent ascitic drainage could interfere with response assessment, we carried out the same analysis excluding patients who had had a recent ascitic or pleural drain. When we excluded patients with recent ascitic drain, the ROC cut-point remained 60% (Fig 4C; S8 Table). Median TTP was 76 d versus 229 d for a decrease of ≤60% and >60% in TP53MAF, respectively, and the HR decreased from 0.22 to 0.08 (95% CI 0.02–0.34, *p <* 0.001; Fig 4D). The sensitivity and specificity of a TP53MAF decrease of ≤60% after one cycle of chemotherapy for predicting TTP < 6 mo was 71% and 88%, respectively, in the whole population, and 75% and 100% in patients without ascitic drains (Table 4; see also S8 and S12 Tables).

**Table 4:** Sensitivity and specificity of a decrease in *TP53* mutant allele fraction and CA-125 for predicting 6-mo time to progression following one cycle of chemotherapy.

Predictor	Sensitivity (95% CI)	Specificity (95% CI)	Negative Predictive Value (95% CI)	Positive Predictive Value (95% CI)
**TP53MAF: ≤60% decrease from cycle 1 to 2**				
All (*n*_courses_ = 31)*	71% (42%–92%)	88% (64%–99%)	79% (54%–94%)	83% (52%–98%)
Excluding drains (*n*_courses_ = 24)*	75% (43%–95%)	100% (74%–100%)	80% (52%–96%)	100% (66%–100%)
**CA-125: ≤50% decrease from cycle 1 to 2**				
All (*n*_courses_ = 31)*	93% (66%–100%)	29% (10%–56%)	83% (36%–100%)	52% (31%–72%)
Excluding drains (*n*_courses_ = 24)*	92% (62%–100%)	33% (9%–65%)	80% (28%–99%)	58% (34%–80%)

*One course of chemotherapy was excluded from the sensitivity/specificity analysis for 6-mo time to progression since it was censored before 6 mo.

CI, confidence interval; TP53MAF, *TP53* mutant allele fraction.

The predictive value of TP53MAF remained significant when we looked at changes from pre-treatment to pre-cycle 3 of chemotherapy (as compared with changes to pre-cycle 2, above). The optimal cut-point was selected at a decrease of 80% for this time point (S13–S17 Tables). Using an 80% decrease threshold, the HR for TTP after two cycles in multivariable analysis was 0.26 (95% CI 0.07–0.92, *p =* 0.037). Response classification after one cycle of chemotherapy and after two cycles of chemotherapy was consistent (S6 Fig).

## Discussion

We describe here our analysis of patient-specific *TP53* mutations in ctDNA in women with HGSOC. We used sequence-specific assays to detect predefined *TP53* mutations and to quantify the TP53MAF by digital PCR with sensitivity down to 0.15%. In plasma samples collected prior to treatment for relapsed disease, we were able to detect mutated alleles at ≥20 amplifiable copies/ml in 82% of treatment courses, and in 86% of newly diagnosed patients.

We compared ctDNA to tumour volume, using volumetric analysis of CT images. Although tumour volume appears likely to be an important prognostic factor, this has not been extensively studied [40–44]. In our limited sample set, 3-D tumour volume was significantly associated with TTP in univariable analysis, but did not emerge as significantly associated with TTP in multivariable analysis including TP53MAF, TP53MAC, and other data (Tables 2 and S10). Our data suggest that TP53MAF contains more information on prognosis than CT imaging. This finding agrees with findings from other studies using CT imaging to track metastatic cancer, for example in breast cancer, where a rising level of ctDNA was found to be an earlier indicator of disease relapse than CT imaging [16].

In our study, ctDNA (TP53MAF and TP53MAC) showed significant correlation with disease volume, particularly in patients without ascites. This is consistent with previous studies that showed that ctDNA level increases as stage increases across a range of different tumour types [15]. For example, in recurrent CRC, significant correlations have been demonstrated between pre-treatment ctDNA levels and both RECIST and carcinoembryonic antigen measurements [22]. In addition, in a study of untreated lung cancer, there was significant concordance between ctDNA level and tumour volume in nine patients [17]. The weaker correlation between tumour volume and TP53MAF and TP53MAC levels observed when including patients with ascites, and the rapid change in plasma TP53MAF observed after ascitic drainage (Fig 3F), suggests that ascites fluid may be a reservoir for cell-free tumour DNA. CA-125 was abnormal in all patients at baseline. However, CA-125 has low specificity and may be elevated by any malignant peritoneal process, which may explain its poorer correlation with tumour volume [45].

We found that the ratio of TP53MAF to disease volume was higher in relapsed patients than in newly diagnosed patients. Potential explanations for higher TP53MAF in patients with recurrent disease include disruption of the peritoneum after surgery, differences in tumour biology, different rates of ctDNA release from different metastatic sites, and differences in DNA lifetime in circulation as a result of other physiological changes. With a detection cut-off of 20 AC/ml, ctDNA was detected in all but one relapsed patient with more than 32 cm^3^ of disease, and in some patients with lower volume of disease. Previous analysis of tumour volume in lung cancer showed detection of ctDNA with 5–20 cm^3^ of disease [17]. These data support the notion that ctDNA has the potential for use in screening and earlier diagnosis of cancer. For earlier diagnosis in symptomatic women, it may be advantageous to combine ctDNA and CA-125 assays to increase specificity as well as sensitivity.

We examined ctDNA (TP53MAF and TP53MAC) levels prior to the start of treatment as a possible marker for prognosis. Platinum sensitivity, as defined by disease-free or treatment-free interval, is currently the most clinically useful prognostic factor for TTP in relapsed patients [46–50]. We compared TP53MAF and TP53MAC to platinum sensitivity and to established prognostic markers for relapsed ovarian cancer including CA-125 and disease volume. In multivariable analysis, TP53MAF and platinum sensitivity remained significant predictive factors. At present, interventional trials in relapsed ovarian cancer stratify by platinum sensitivity. If our results are confirmed in larger studies, then additional stratification by ctDNA level could increase the accuracy of outcome prediction.

There is also a clinical need for rapid detection of early response to therapy in HGSOC. CA-125, which has been previously evaluated for this purpose, is not sufficiently predictive to be used as a primary endpoint in clinical trials. A large retrospective analysis of the CALYPSO phase III trial in relapsed HGSOC unexpectedly showed that early decline in CA-125 was more likely in the inferior arm, leading the authors to conclude that early change in CA-125 is a poor surrogate for progression-free survival [51]. More recently, alternative criteria for CA-125 response in clinical trials have been explored [52]; however, no reliable model has yet been identified for biomarker-driven trials.

We therefore analysed the dynamics of *TP53* ctDNA during treatment with standard of care chemotherapy, to explore its potential as an early response marker. We found that the mean time to nadir was shorter for TP53MAF than for CA-125 (37 d versus 84 d). This may be explained by the longer half-life of CA-125 of 10 d [53] compared with 1–2 h for ctDNA [11,12]. In relapsed treatment courses, a decrease of ≤60% in TP53MAF after one cycle identified a group with progression within 6 mo with high specificity (88% overall; 100% of courses when patients with recent ascitic drains were excluded). In multivariable analysis, TP53MAF was an independent predictor of 6-mo TTP after one and two cycles of chemotherapy. Importantly, patients in the group with poorer prognosis after one cycle did not change group after the second cycle. However, this is a small retrospective study, and variability in the study individuals and their treatments may account for these effects. Future studies are needed to validate a 60% decrease in TP53MAF after one cycle as the optimum clinically useful threshold and to validate other findings from this study.

A similar study in metastatic colon cancer with a prospective design analysed the predictive value of changes in ctDNA after one cycle of chemotherapy in 53 patients [22]. The single ctDNA response marker for each patient was chosen from a panel of genes that are commonly mutated in CRC, and an association with increased progression-free survival was observed in patients with a ≥90% decrease in ctDNA, although this outcome did not reach significance (HR = 1.87, *p =* 0.266). A potential advantage of quantifying *TP53* mutations for response in HGSOC is that *TP53* mutation is the earliest known driver event in HGSOC and is detectable in all metastatic disease [54,55]. By contrast, in other cancers, intratumoural heterogeneity and clonal diversity may reduce the accuracy of using any single mutation in ctDNA as a quantitative measure of tumour burden and as a predictor of response [56,57]. We observed rapid increase in TP53MAF in a patient who developed new cerebral metastasis, suggesting that, in HGSOC, ctDNA changes are not limited to abdominal disease.

These findings need replication but may have a significant impact for patients with HGSOC, particularly if ctDNA can be developed as an early predictor of treatment efficacy. The phase III SWOG S0500 trial tested whether circulating tumour cells could be used as an early predictor of response in metastatic breast cancer, but early switching of therapy did not show improved overall survival [58]. However, the negative results may reflect a lack of active drugs for these patients, or methods of insufficient sensitivity, rather than the usefulness of the predictor. In metastatic breast cancer, ctDNA was shown to have >100-fold higher copy numbers in plasma compared to the number of circulating tumour cells detected by the CELLSEARCH system, which was the method used in the SWOG S0500 study [16].

The major limitations of this study are its retrospective design, analysis of multiple courses from the same patient, limited sample size and sampling times, and the heterogeneity of treatment within the cohort. In addition, as this was a proof of concept study, we analysed DNA from only a small volume of plasma (median of approximately 0.1 ml per sample) to conserve research material. Even in such a limited volume of plasma, we detected ctDNA at ≥20 AC/ml in >80% of pre-treatment plasma samples and were able to show the association of TP53MAF with TTP. Future studies aiming to validate this approach could further enhance the sensitivity and accuracy of ctDNA measurements by using larger volumes of plasma. The technology for assessment of ctDNA that was used in this study is based on fluorescently labelled patient-specific probes. Such assays can be expensive and time-consuming to design and validate. Since this study was initiated, we and others have demonstrated that suitably designed next generation sequencing assays can be used with high sensitivity both for monitoring ctDNA levels and for direct identification of mutations for genotyping of tumour via plasma sampling [16,21]. The use of such panel assays can obviate the need to design patient-specific assays targeting individual mutations, simplifying the deployment of such approaches for clinical use, and allowing multiple mutations to be monitored simultaneously [17,21].

Recent studies have demonstrated the potential of ctDNA as a tool for minimally invasive real-time molecular profiling, to identify risk of progression based on residual disease, and to identify disease recurrence earlier. In this study, we showed the potential of ctDNA to identify, after 1–2 cycles of treatment, ovarian cancer patients with an expected poor response to chemotherapy. These findings have strong potential for clinical utility owing to the ease of assaying DNA in plasma and the low cost and speed of ctDNA testing. There is therefore a strong rationale for including ctDNA collection in current clinical trials as exploratory endpoints to support clinical validation of ctDNA as a potential early marker of response and prognosis. Having very early information on response would empower patients and physicians to test alternative treatment options and would have high utility in trials that link biomarkers to targeted therapy [59].

## Supporting Information

S1 Checklist STROBE statement—checklist of items that should be included in reports of observational studies. (DOC)

S1 Data Circulating tumour DNA CA-125 chemotherapy RECIST and procedures. (XLSX)

S2 Data Survival data. (XLSX)

S3 Data Volumetric data. (XLSX)

S1 Fig REMARK diagram of patient selection for circulating tumour DNA quantitation. (PPTX)

S2 Fig Assessment of assay performance in control experiments. (DOCX)

S3 Fig Graphs of *TP53* mutant fraction and CA-125 changes by patient. (PDF)

S4 Fig REMARK diagram of course selection for analysis of circulating tumour DNA pre-treatment and for circulating tumour DNA change after the first and second cycle of chemotherapy. (PPTX)

S5 Fig Receiver operating characteristic curve for circulating tumour DNA decrease after two cycles of chemotherapy to predict 6-mo time to progression and Kaplan-Meier curves including and excluding patients with ascitic drains. (DOCX)

S6 Fig Consistency of predictive category by *TP53* mutant allele fraction decrease after one and two cycles in all patients and excluding patients with recent ascitic drains. (DOCX)

S1 Table Sequence *TP53* mutations, primer sequences, and amplicon size. (XLSX)

S2 Table Demographic information by patient. (XLSX)

S3 Table Context of circulating tumour DNA samples collected in study relative to chemotherapy cycle. (A) Samples from chemotherapy at relapse. (B) Samples collected during first-line treatment.
(PDF)

S4 Table Pre-treatment descriptive statistics for *TP53* mutant allele fraction, *TP53* mutant allele count, *TP53* total allele count, and CA-125. (DOCX)

S5 Table Pearson correlation of pre-treatment *TP53* mutant allele fraction, *TP53* mutant allele count, *TP53* total allele count, and CA-125 in relapsed patients. (DOCX)

S6 Table Pearson correlation of blood parameters with volume of disease in relapsed patients. (DOCX)

S7 Table Inclusion criteria for courses of treatment eligible for nadir analysis. (DOCX)

S8 Table Sensitivity and specificity by *TP53* mutant allele fraction decrease after one cycle including and excluding courses with recent ascites drains. (DOCX)

S9 Table Univariable and multivariable analysis of decrease in *TP53* mutant allele fraction as a continuous variable to predict time to progression after one cycle of chemotherapy. (DOCX)

S10 Table Univariable and multivariable analysis of pre-treatment *TP53* mutant allele count as a continuous variable to predict time to progression. (DOCX)

S11 Table Univariable and multivariable analysis of decrease in *TP53* mutant allele count as continuous variable to predict time to progression after one cycle of chemotherapy. (DOCX)

S12 Table Sensitivity and specificity of CA-125 decrease after one cycle including and excluding courses with recent ascites drains. (DOCX)

S13 Table Univariable and multivariable analysis of decrease in *TP53* mutant allele fraction as a continuous variable to predict time to progression after two cycles of chemotherapy. (DOCX)

S14 Table Sensitivity and specificity of *TP53* mutant allele fraction decrease after two cycles including and excluding courses with recent ascites drains. (DOCX)

S15 Table Univariable and multivariable analysis of *TP53* mutant allele fraction decrease of >80% after two cycles of chemotherapy as a predictor of time to progression. (DOCX)

S16 Table Sensitivity and specificity of CA-125 decrease after two cycles including and excluding courses with recent ascites drains. (DOCX)

S17 Table Sensitivity and specificity of decrease in *TP53* mutant allele fraction and CA-125 for predicting 6-mo time to progression following one and two cycles of chemotherapy. (DOCX)

S1 Text Statistical analysis plan, September 2015. (DOCX)

## Abbreviations

AC/ml: amplifiable copies per millilitre of plasma
CRC: colorectal cancer
CT: computed tomography
ctDNA: circulating tumour DNA
FFPE: formalin-fixed paraffin-embedded
HGSOC: high-grade serous ovarian carcinoma
HR: hazard ratio
IQR: interquartile range
PS: performance status
RECIST: Response Evaluation Criteria in Solid Tumours
ROC: receiver operating characteristic
TP53MAC: *TP53* mutant allele count
TP53MAF: *TP53* mutant allele fraction
TP53TAC: *TP53* total allele count
TTP: time to progression
