# Supplementary material for: Exploratory Analysis of TP53 Mutations in Circulating Tumour DNA as Biomarkers of Treatment Response for Patients with Relapsed High-Grade Serous Ovarian Carcinoma: A Retrospective Study
Source: PLoS Med. 2016 Dec 20;13(12):e1002198. doi: 10.1371/journal.pmed.1002198 (PMC5172526; doi:10.1371/journal.pmed.1002198)
Supplement: S1 Fig — (PPTX) [file pmed.1002198.s005.pptx]

## Slide 1
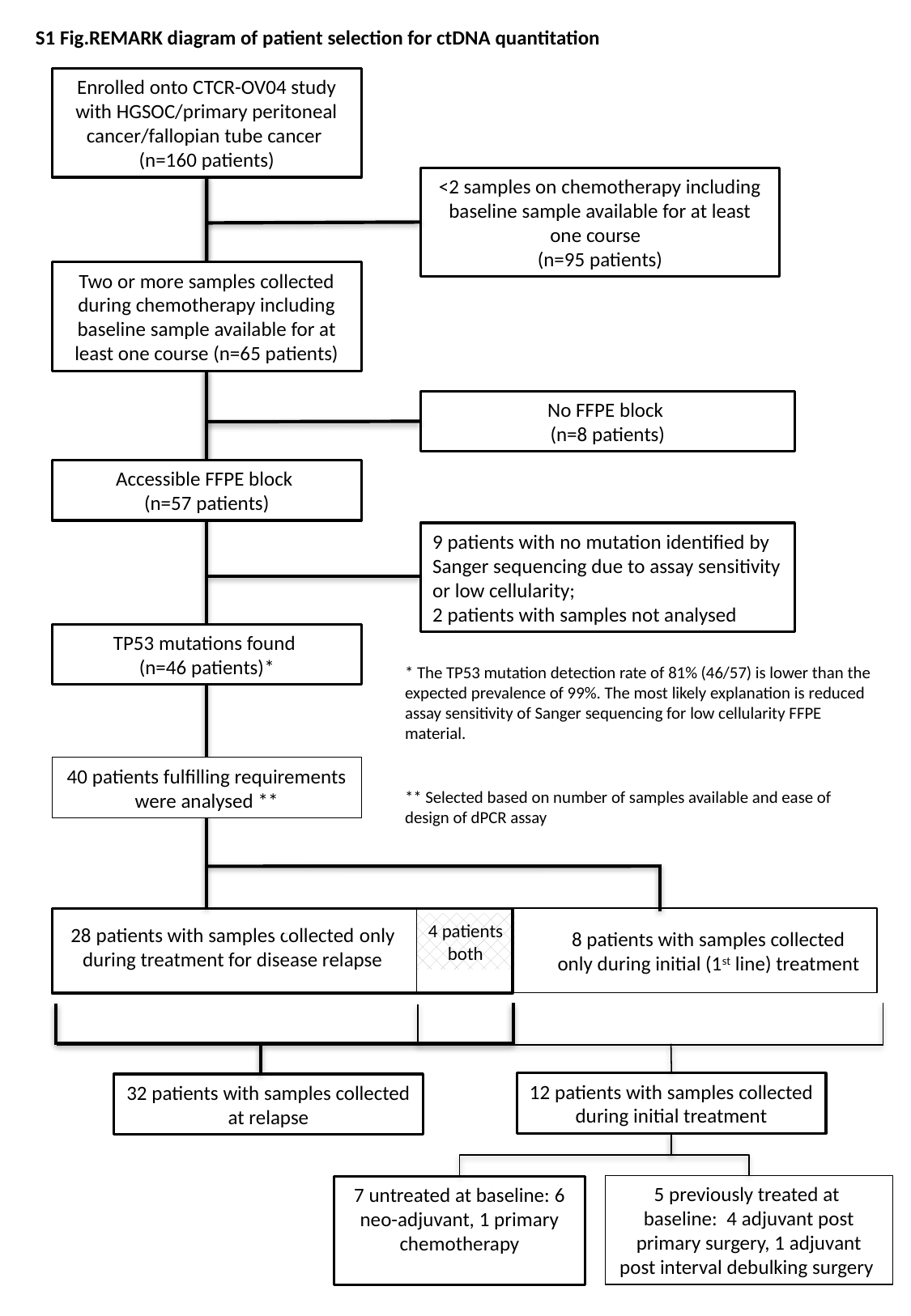

S1 Fig.REMARK diagram of patient selection for ctDNA quantitation
Enrolled onto CTCR-OV04 study with HGSOC/primary peritoneal cancer/fallopian tube cancer
(n=160 patients)
<2 samples on chemotherapy including baseline sample available for at least one course
(n=95 patients)
Two or more samples collected during chemotherapy including baseline sample available for at least one course (n=65 patients)
No FFPE block
(n=8 patients)
Accessible FFPE block
(n=57 patients)
9 patients with no mutation identified by Sanger sequencing due to assay sensitivity or low cellularity;
2 patients with samples not analysed
TP53 mutations found
(n=46 patients)*
* The TP53 mutation detection rate of 81% (46/57) is lower than the expected prevalence of 99%. The most likely explanation is reduced assay sensitivity of Sanger sequencing for low cellularity FFPE material.
40 patients fulfilling requirements were analysed **
** Selected based on number of samples available and ease of design of dPCR assay
28 patients with
28
4 patients both
28 patients with samples collected only during treatment for disease relapse
8 patients with samples collected only during initial (1st line) treatment
12 patients with samples collected during initial treatment
32 patients with samples collected at relapse
5 previously treated at baseline: 4 adjuvant post primary surgery, 1 adjuvant post interval debulking surgery
7 untreated at baseline: 6 neo-adjuvant, 1 primary chemotherapy
