# Supplementary material for: Exploratory Analysis of TP53 Mutations in Circulating Tumour DNA as Biomarkers of Treatment Response for Patients with Relapsed High-Grade Serous Ovarian Carcinoma: A Retrospective Study
Source: PLoS Med. 2016 Dec 20;13(12):e1002198. doi: 10.1371/journal.pmed.1002198 (PMC5172526; doi:10.1371/journal.pmed.1002198)

S2 Fig. Assessment of assay performance in control experiments. Circulating tumour DNA from a control mix of plasma samples from healthy volunteers was extracted 86 independent times, and *TP53* alleles measured by digital PCR a total of 141 times, using 32 different assays. The 141 measured values of wild-type *TP53* fit a normal distribution (Chi-square probability 0.64, Kolmogorov-Smirnov probability 0.92), with a coefficient of variation of 18%.


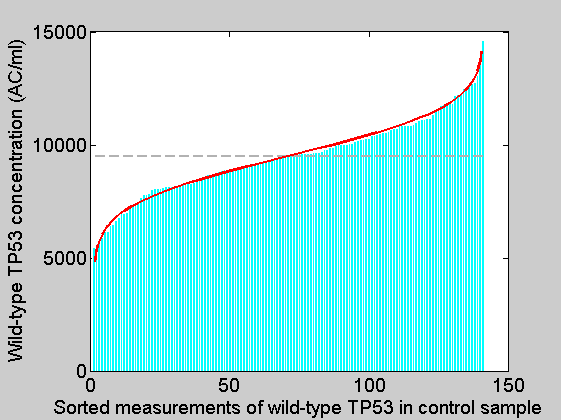

Supplement: S2 Fig — (DOCX) [file pmed.1002198.s006.docx]
