# Supplementary material for: Exploratory Analysis of TP53 Mutations in Circulating Tumour DNA as Biomarkers of Treatment Response for Patients with Relapsed High-Grade Serous Ovarian Carcinoma: A Retrospective Study
Source: PLoS Med. 2016 Dec 20;13(12):e1002198. doi: 10.1371/journal.pmed.1002198 (PMC5172526; doi:10.1371/journal.pmed.1002198)

S3 Fig. Graphs of TP53MAF and CA-125 changes by patient.

Table of Contents

OV04-21	2
OV04-30	3
OV04-33	4
OV04-36	5
OV04-39	6
OV04-47	7
OV04-49	8
OV04-52	9
OV04-57	10
OV04-63	11
OV04-64	12
OV04-65	13
OV04-68	14
OV04-70	15
OV04-72	16
OV04-73	17
OV04-74	18
OV04-75	19
OV04-76	20
OV04-79	21
OV04-81	22
OV04-90	23
OV04-95	24
OV04-97	25
OV04-98	26
OV04-102	27
OV04-105	28
OV04-112	29
OV04-114	30
OV04-116	31
OV04-127	32
OV04-129	33
OV04-133	34
OV04-135	35
OV04-142	36
OV04-144	37
OV04-145	38
OV04-150	39
OV04-161	40
OV04-200	41

OV04-21

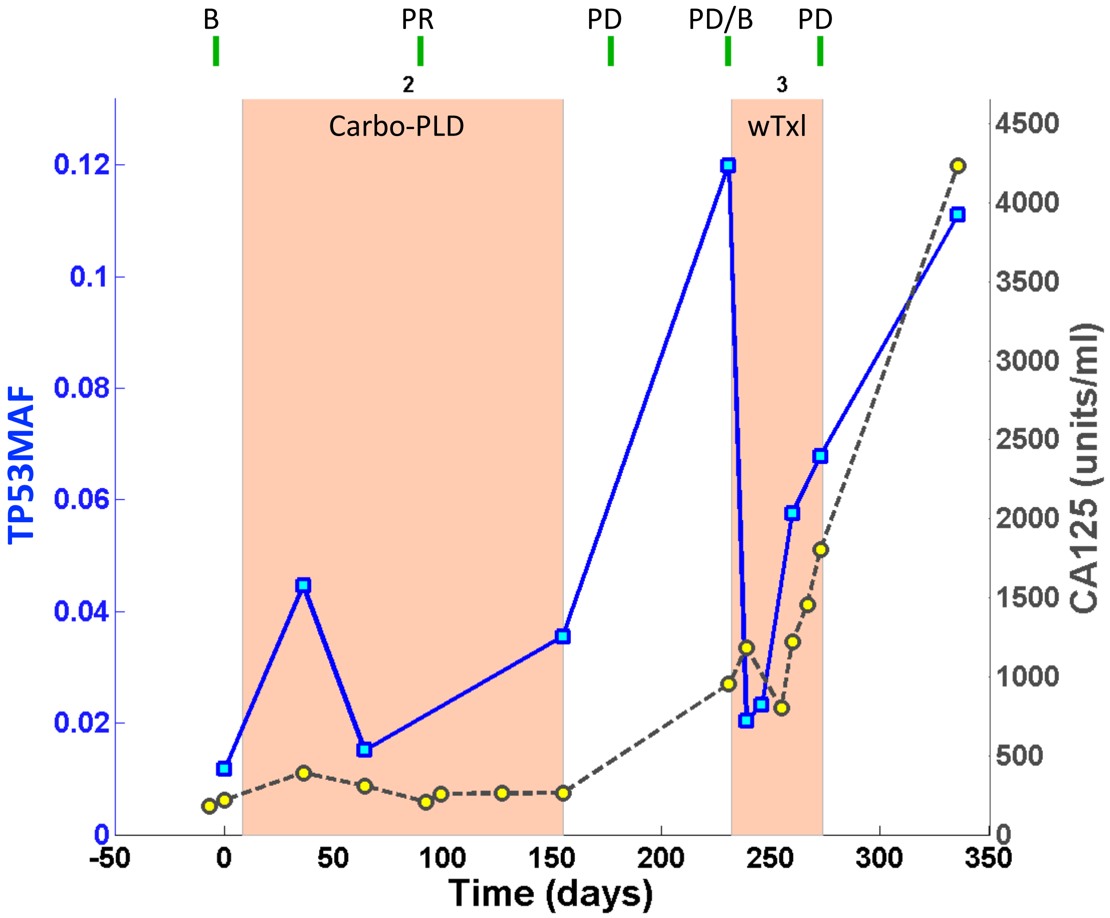

Key:

, ctDNA; , CA-125; , course of chemotherapy; , CT scan; , clinical progression; , ascitic or pleural drain. Number above the orange block refers to the course (line) of treatment. B, baseline CT; CR, complete response on CT; PR, partial response on CT; PD, progressive disease on CT; SD, stable disease on CT. Carbo, carboplatin; Carbo-Txl, carboplatin and paclitaxel; Cis-PLD, cisplatin and liposomal doxorubicin ECX, epirubicin, cisplatin and capecitabine; Gem, gemcitabine; PLD, pegylated liposomal doxorubicin; wTxl, weekly paclitaxel; xTxl+/-sara, weekly paclitaxel +/- saracatinib (on SaPPrOC trial); Topo, topotcan; RT, radiotherapy

OV04-30

Stage at diagnosis: IIIC
Residual disease: no residual

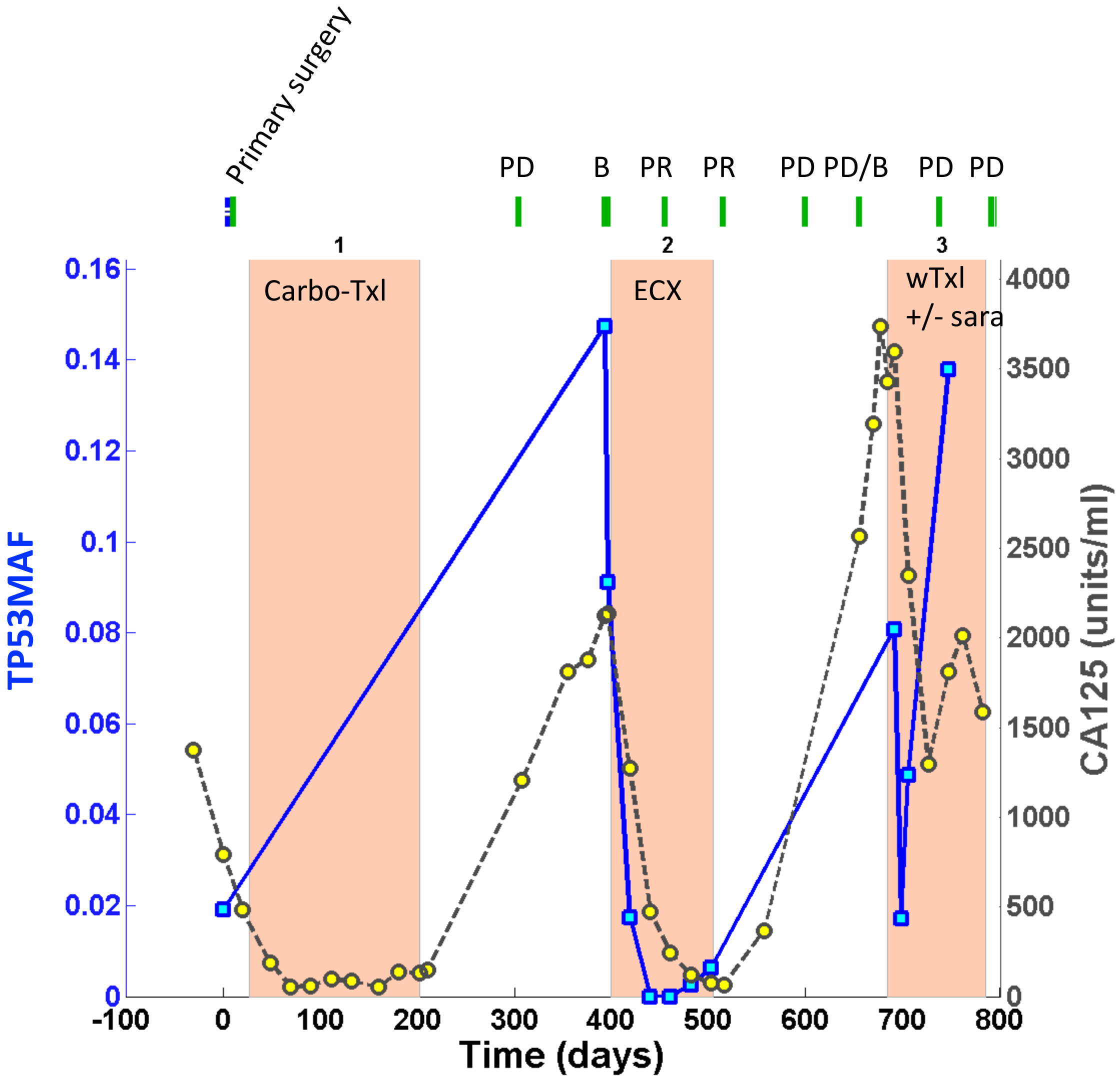

OV04-33

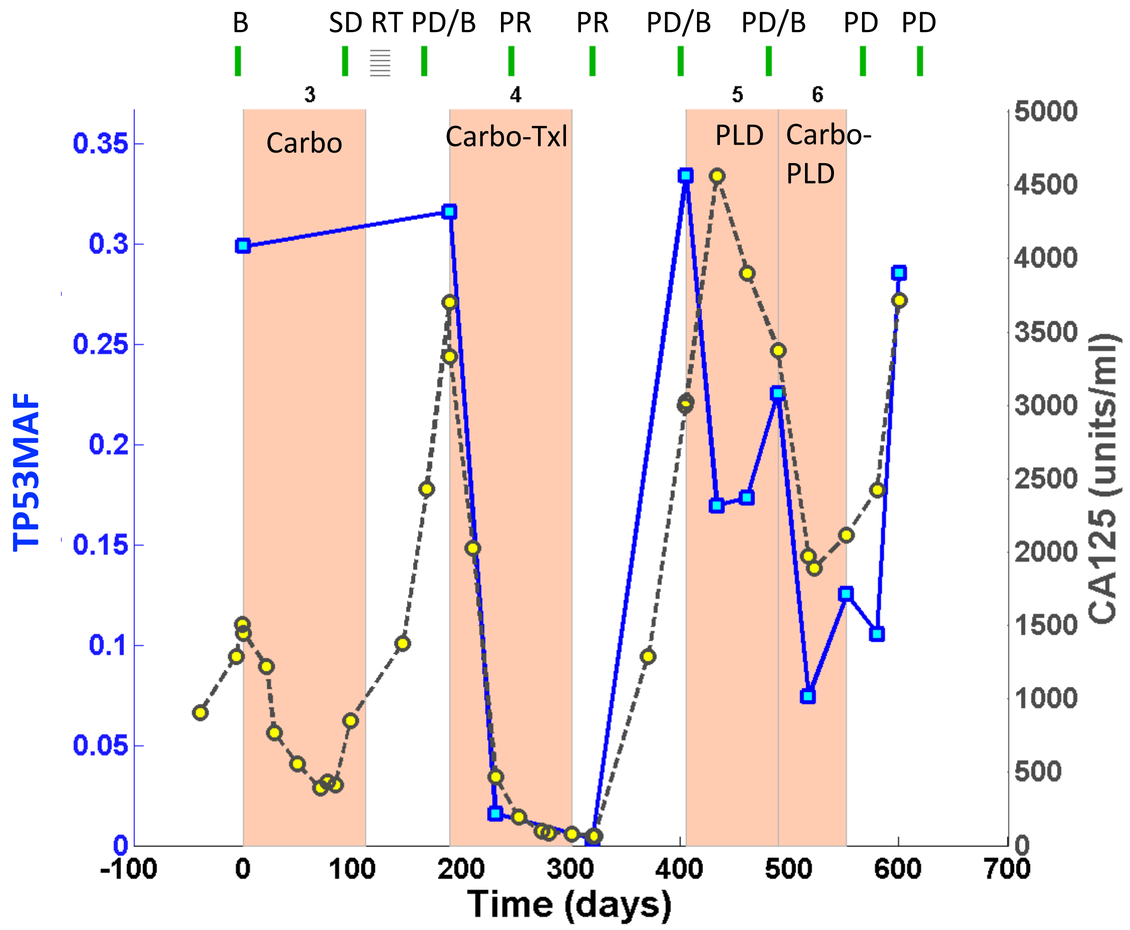

B
SD
RT
PD/B
PR
PR
PD/B
PD/B
PD
PD
3
4
5
6
Carbo
Carbo-Txl
PLD
Carbo-PLD
TP53MAF
CA125 (units/ml)
Time (days)
0.35
0.3
0.25
0.2
0.15
0.1
0.05
0
5000
4500
4000
3500
3000
2500
2000
1500
1000
500
0
-100
0
100
200
300
400
500
600
700

OV04-36

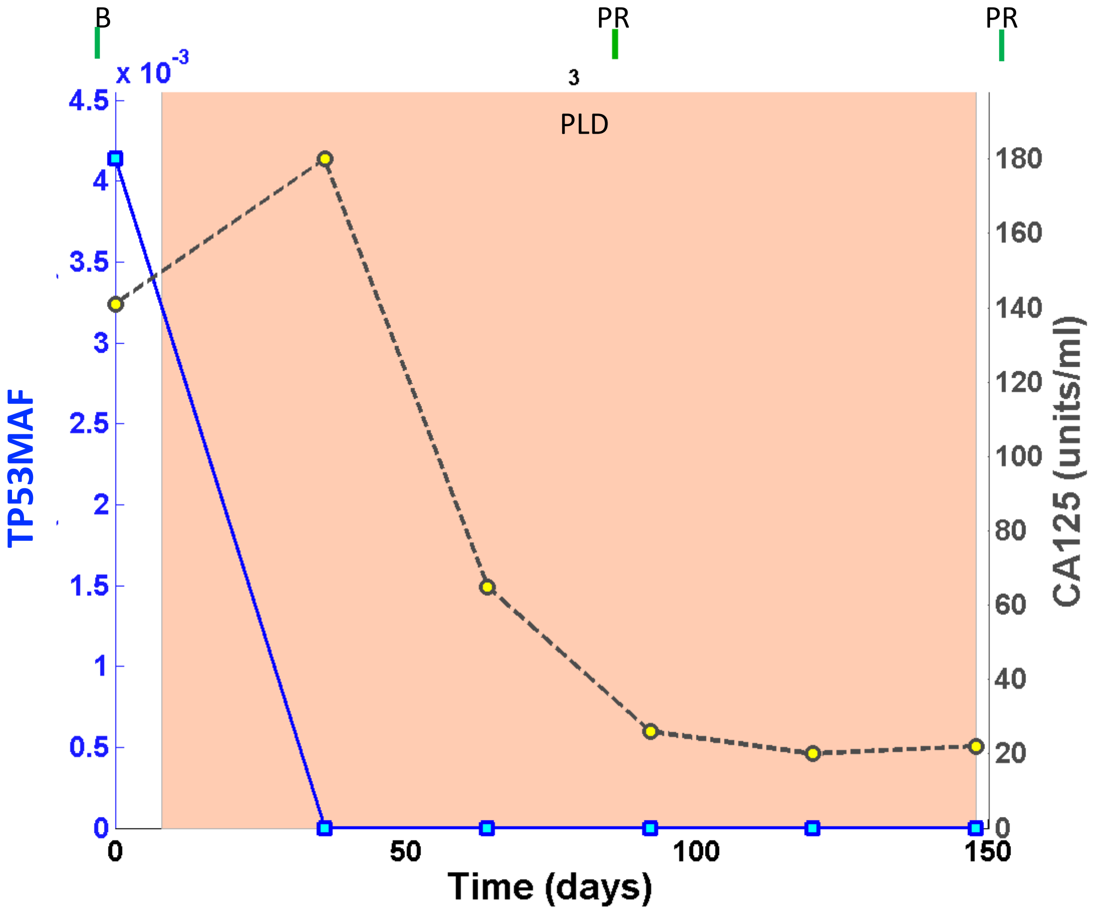
B
PR
PR
x 10^-3
3
PLD
TP53MAF
CA125 (units/ml)
Time (days)
4.5
4
3.5
3
2.5
2
1.5
1
0.5
0
0
50
100
150
180
160
140
120
100
80
60
40
20
0

OV04-39

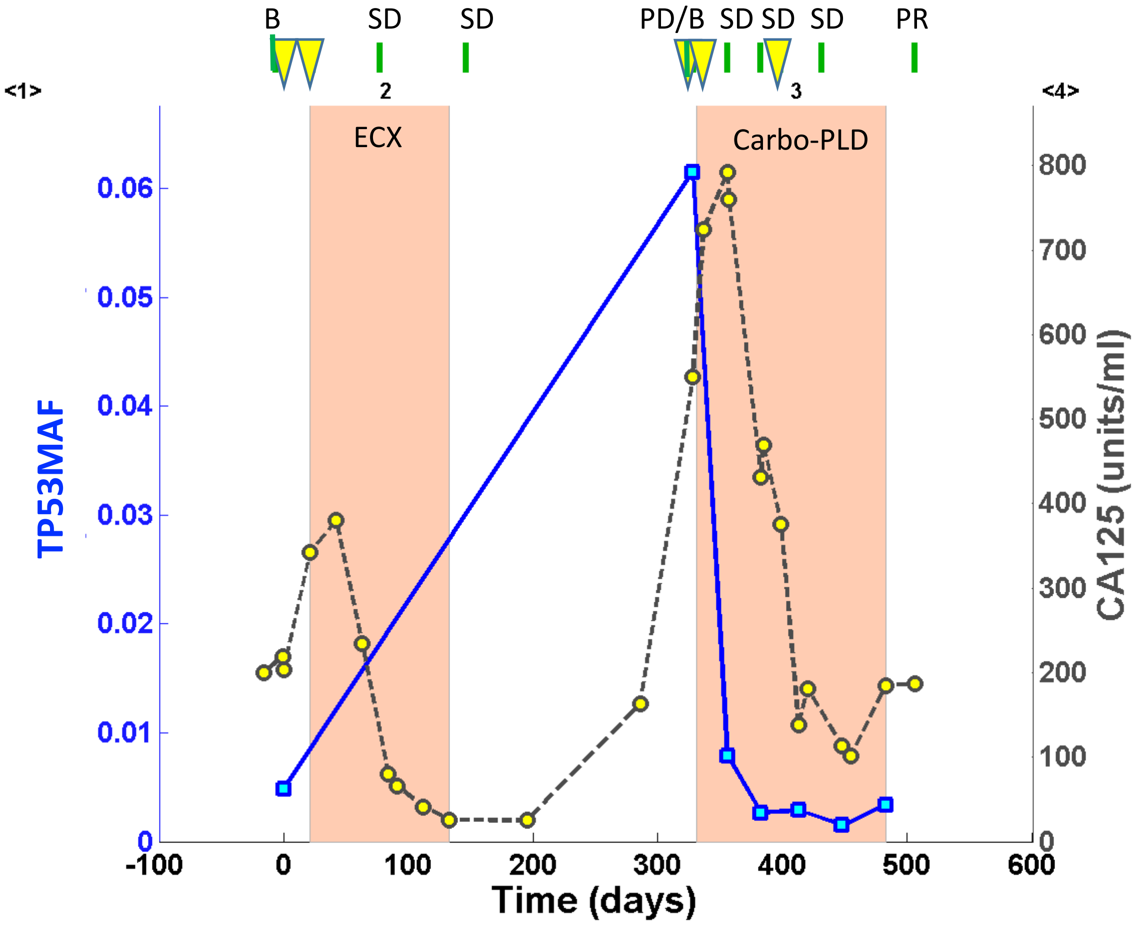
B
SD
SD
PD/B
SD
SD
SD
PR
<1>
2
3
<4>
ECX
Carbo-PLD
TP53MAF
0.06
0.05
0.04
0.03
0.02
0.01
0
800
700
600
500
400
300
200
100
0
CA125 (units/ml)
-100
0
100
200
300
400
500
600
Time (days)

OV04-47

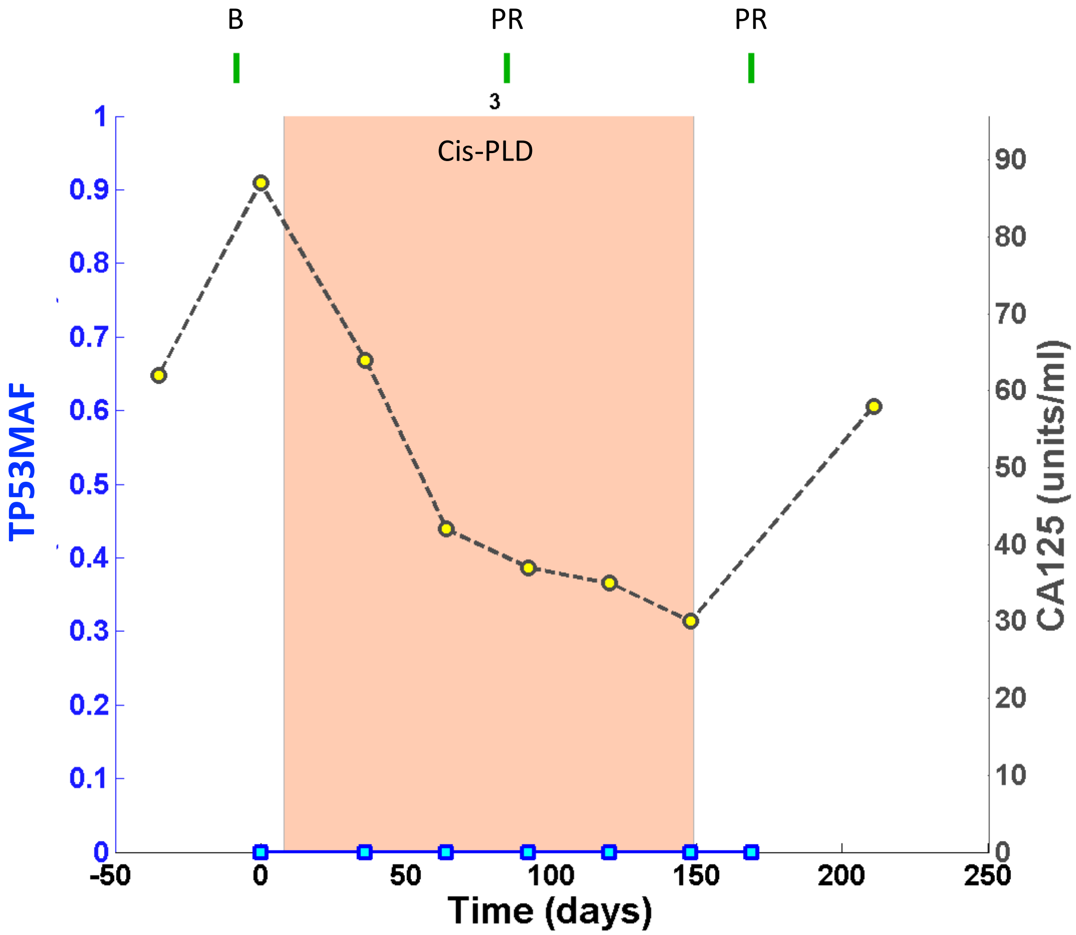

B
PR
PR
3
Cis-PLD
TP53MAF
CA125 (units/ml)
Time (days)
1
0.9
0.8
0.7
0.6
0.5
0.4
0.3
0.2
0.1
0
90
80
70
60
50
40
30
20
10
0
-50
0
50
100
150
200
250

OV04-49

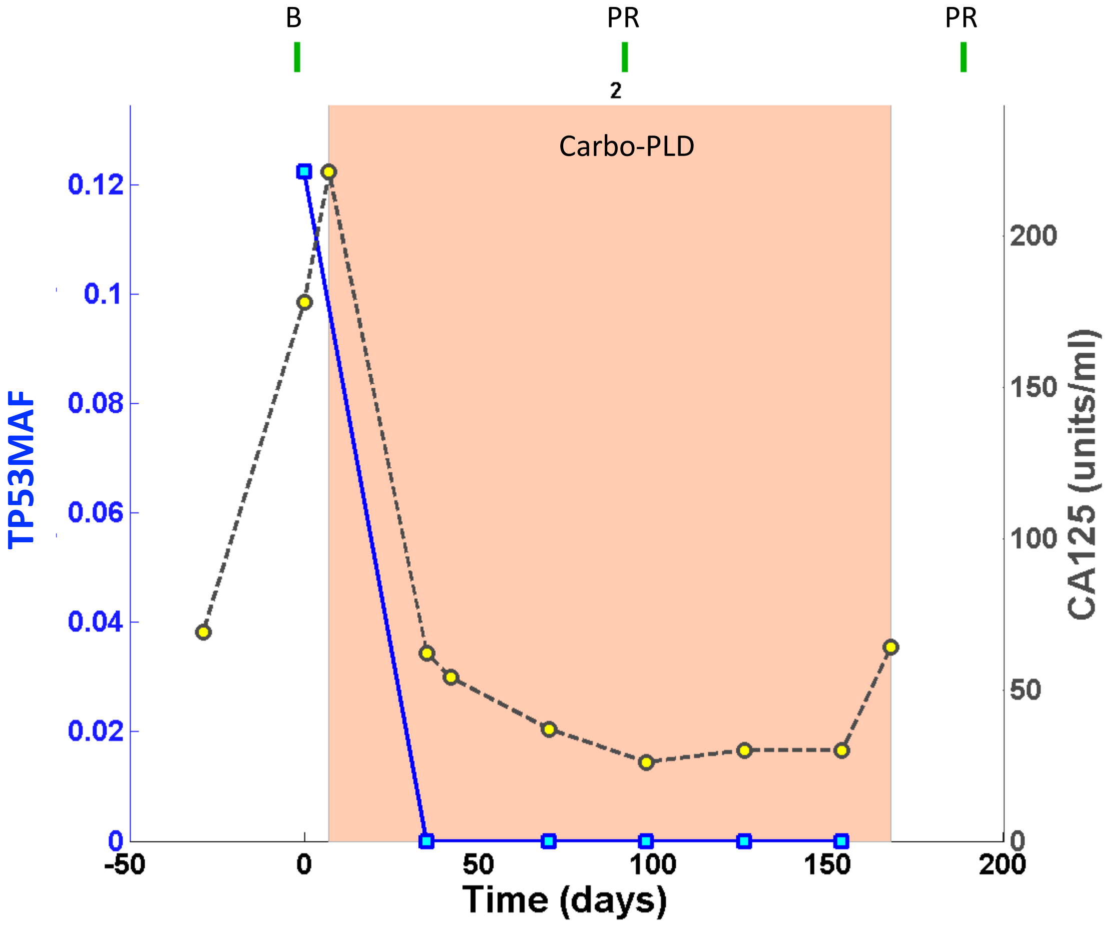

B
PR
PR
2
Carbo-PLD
TP53MAF
0.12
0.1
0.08
0.06
0.04
0.02
0
CA125 (units/ml)
200
150
100
50
0
-50
0
50
100
150
200
Time (days)

OV04-52

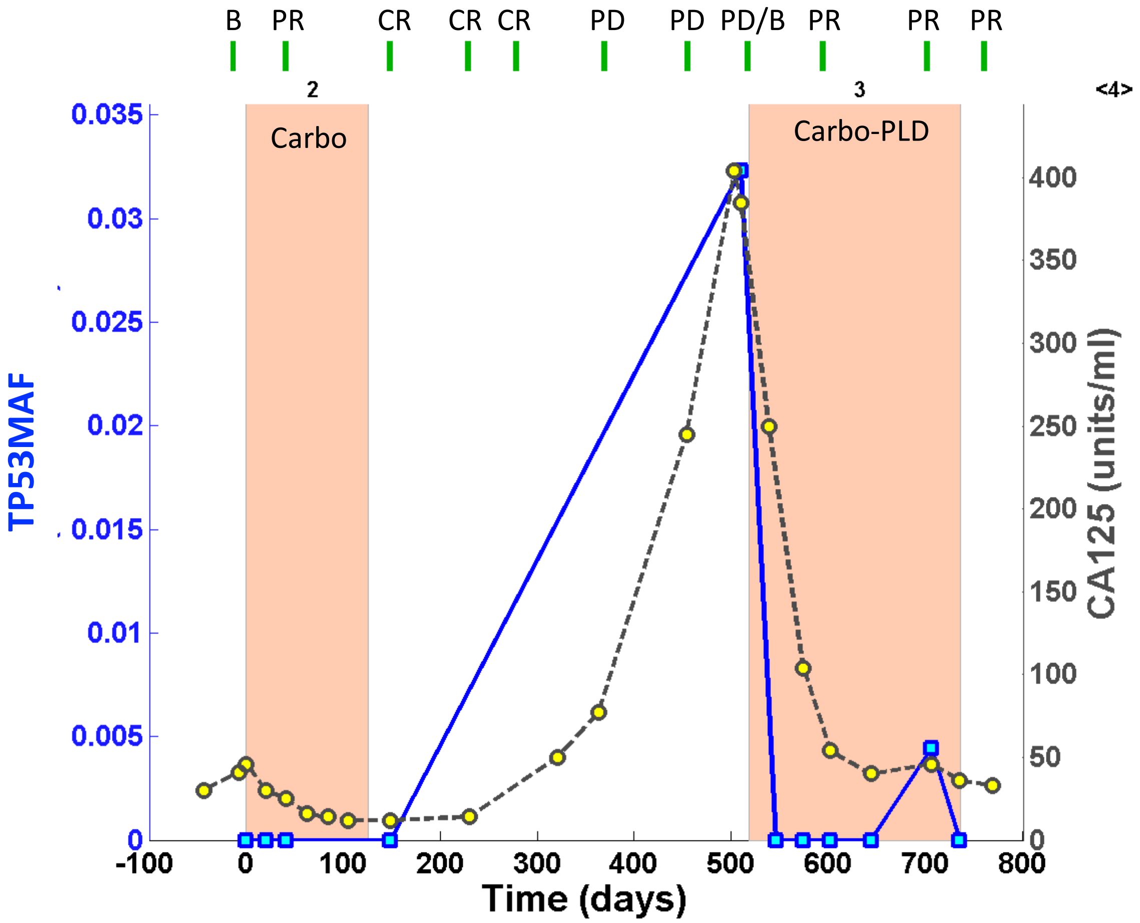

B
PR
CR
CR
CR
PD
PD
PD/B
PR
PR
PR
2
3
<4>
Carbo
Carbo-PLD
TP53MAF
0.035
0.03
0.025
0.02
0.015
0.01
0.005
0
400
350
300
250
200
150
100
50
0
CA125 (units/ml)
-100
0
100
200
300
400
500
600
700
800
Time (days)

OV04-57

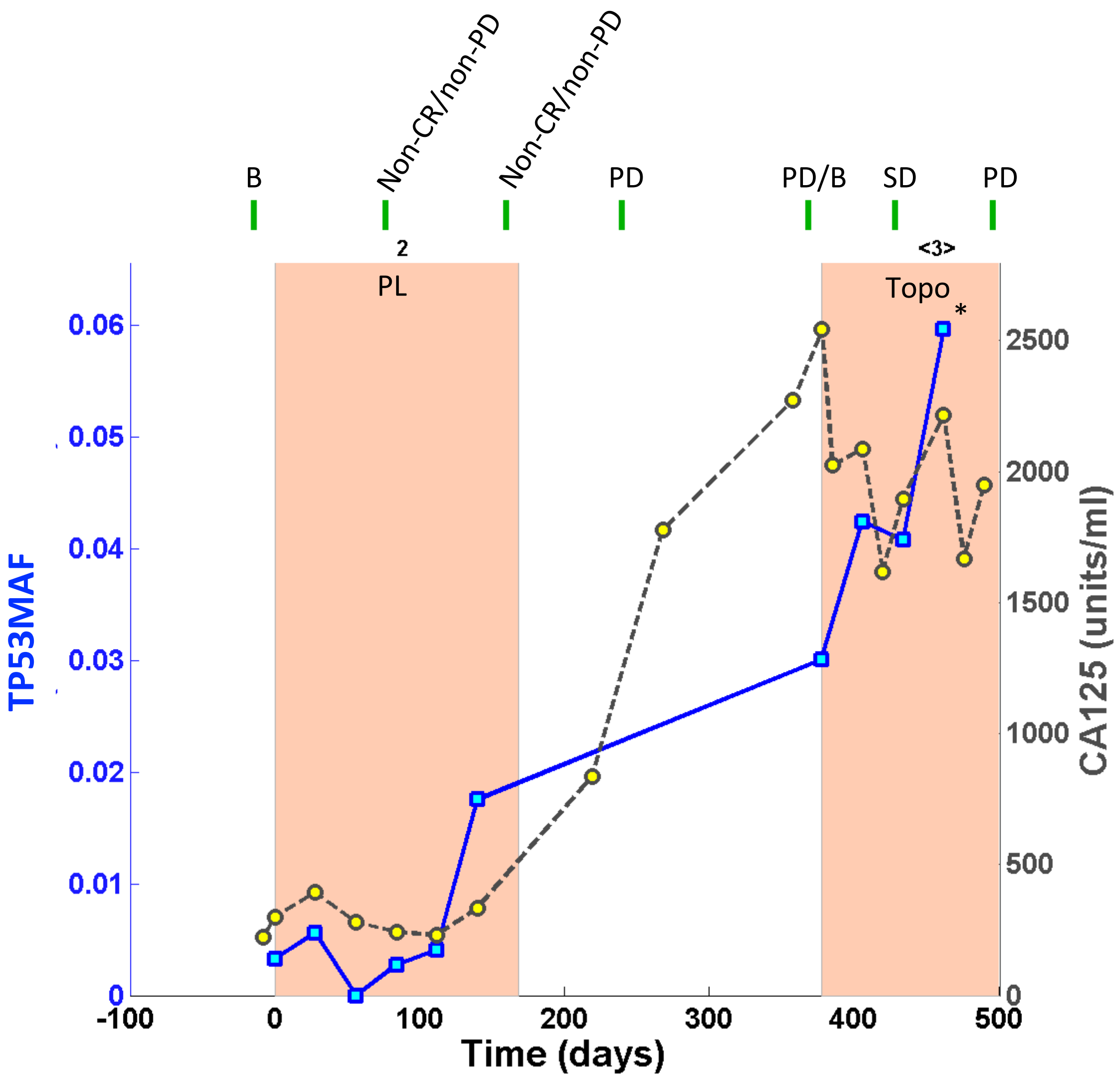

*new onset of headaches. CT shows new brain metastases (PD)

OV04-63

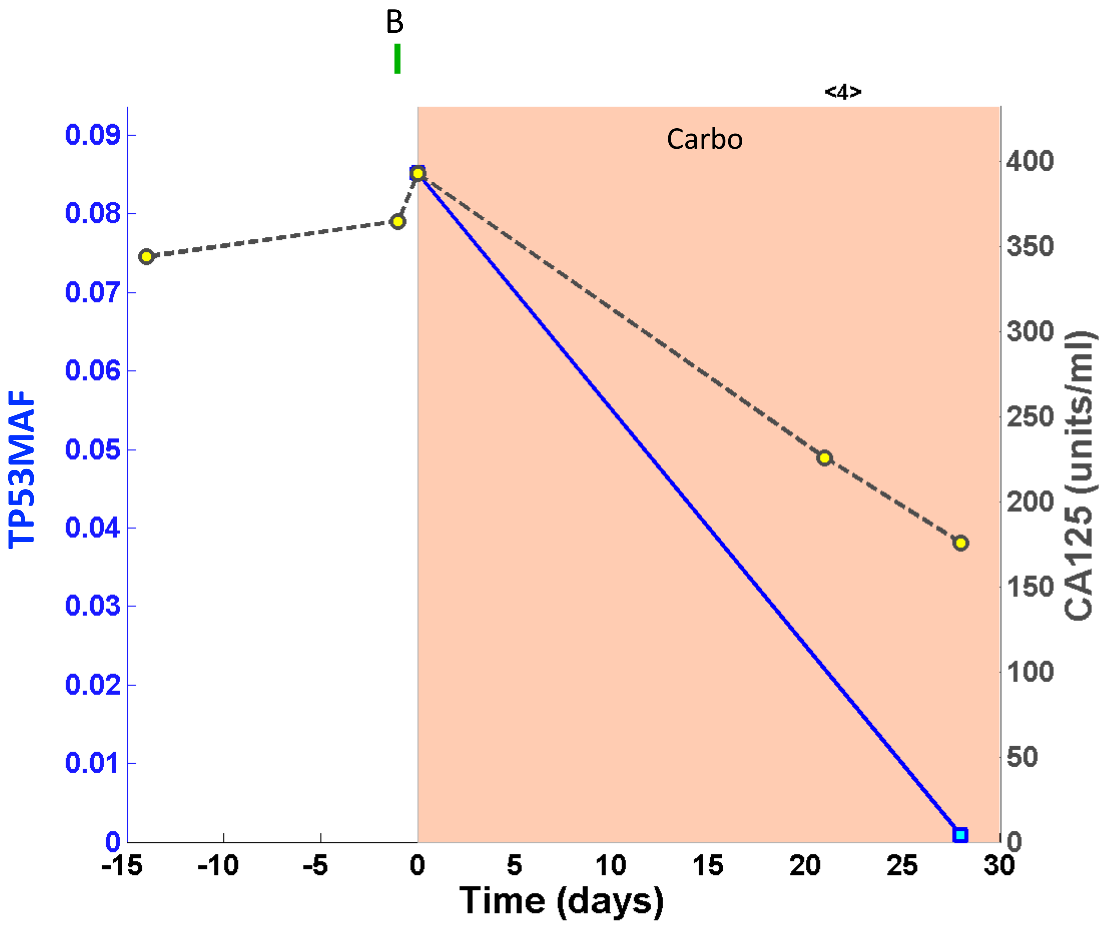

B
<4>
Carbo
0.09
0.08
0.07
0.06
0.05
0.04
0.03
0.02
0.01
0
TP53MAF
400
350
300
250
200
150
100
50
0
CA125 (units/ml)
-15
-10
-5
0
5
10
15
20
25
30
Time (days)

OV04-64

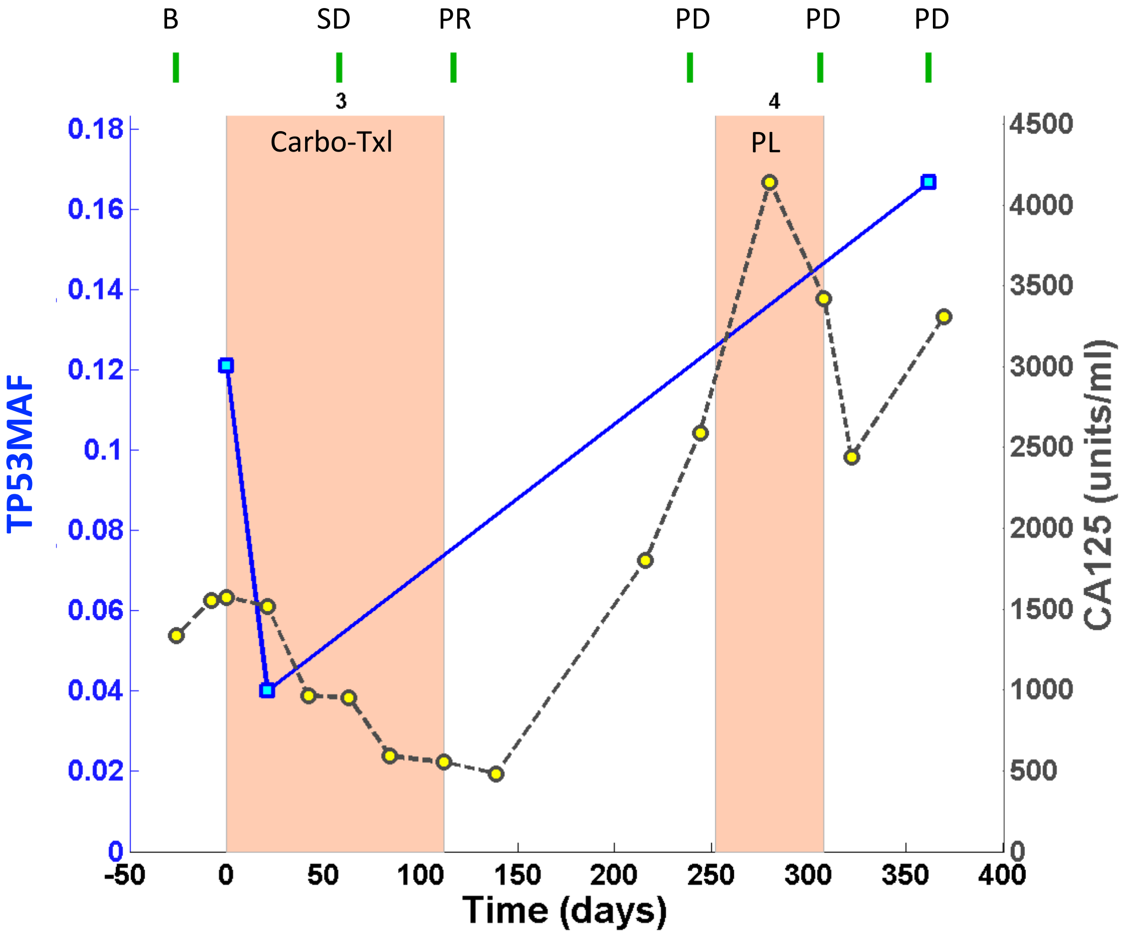

B
SD
PR
PD
PD
PD
3
4
Carbo-Txl
PL
TP53MAF
CA125 (units/ml)
Time (days)
0.18
0.16
0.14
0.12
0.1
0.08
0.06
0.04
0.02
0
4500
4000
3500
3000
2500
2000
1500
1000
500
0
-50
0
50
100
150
200
250
300
350
400

OV04-65

Stage at diagnosis: IV
Residual disease: no residual

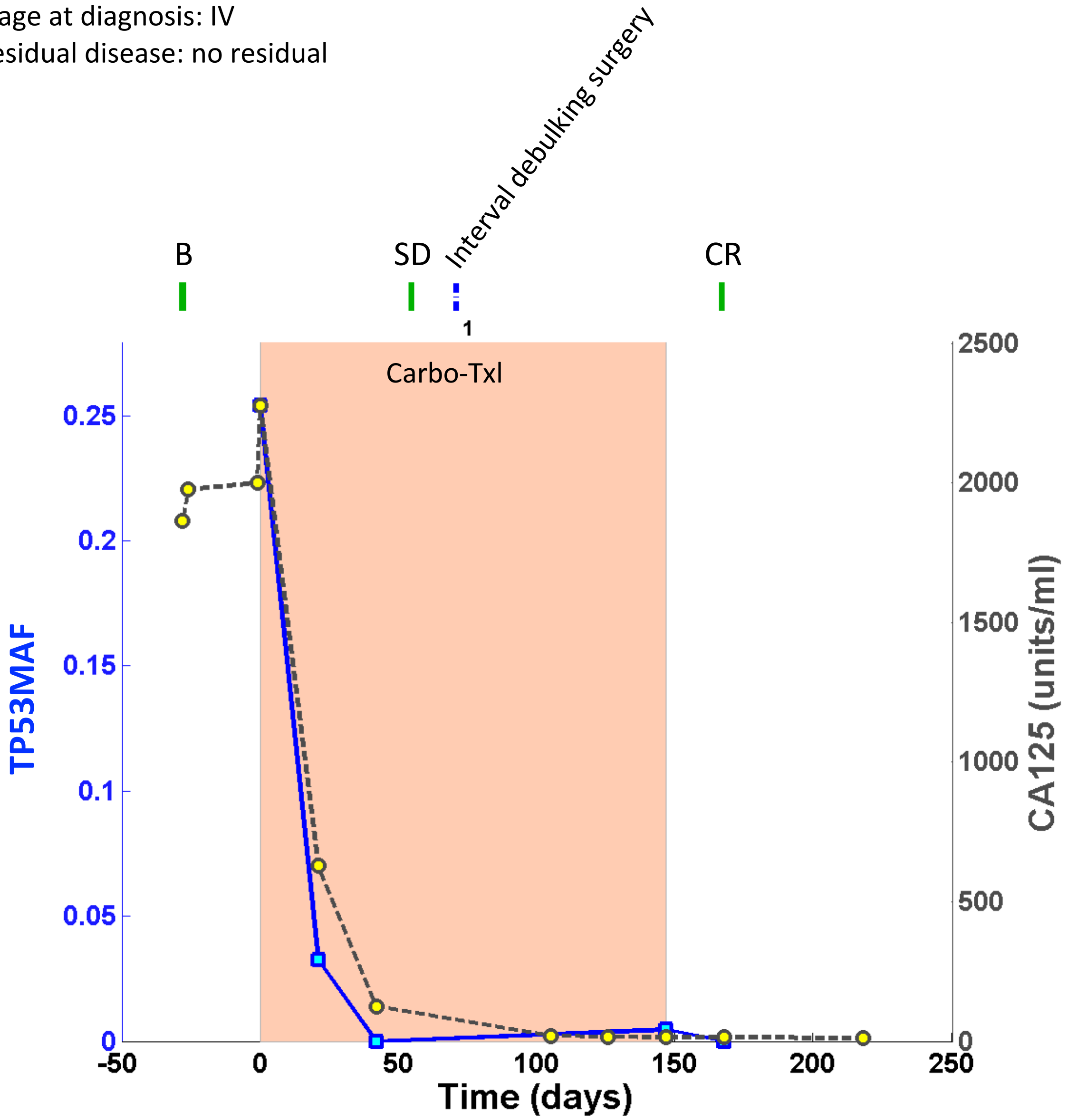

OV04-68

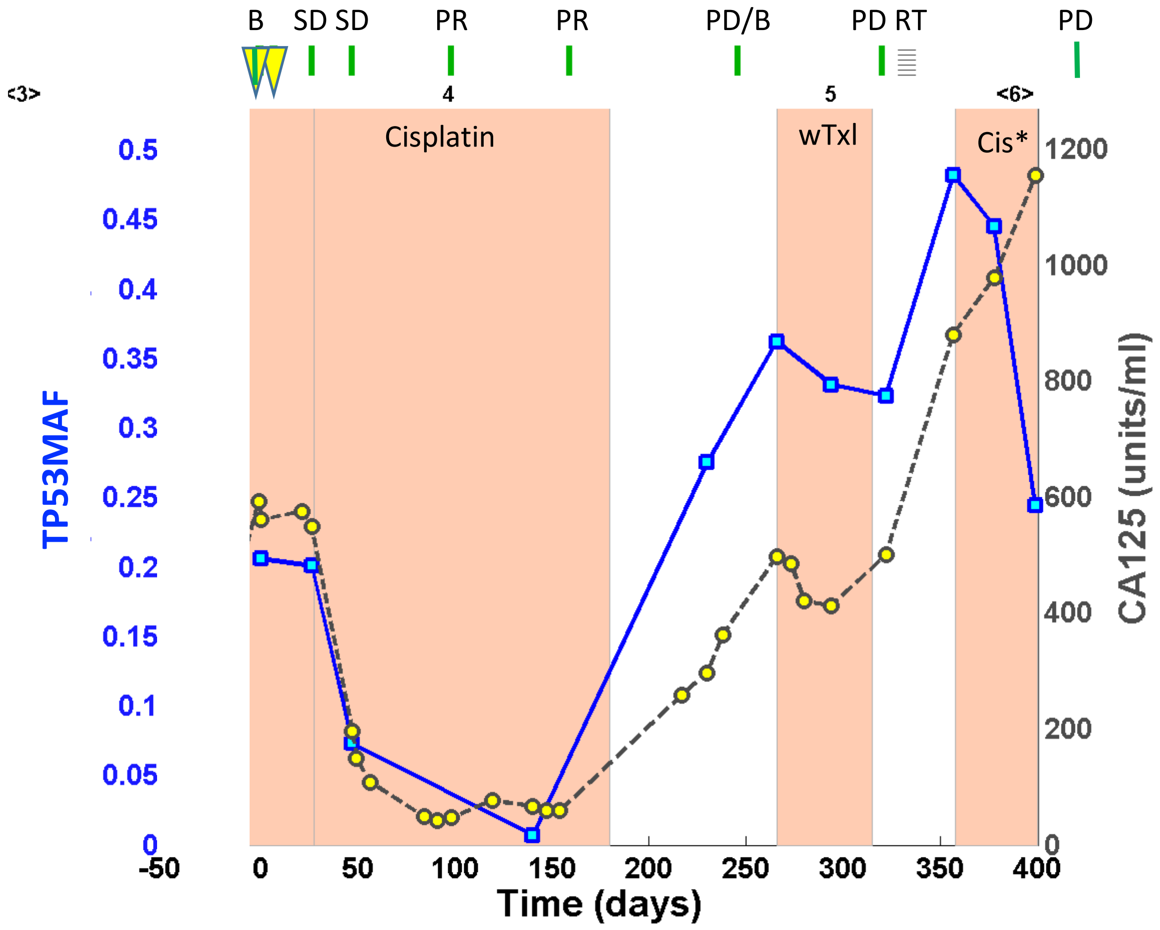

*This patient had discordant ctDNA and CA-125 on her 6th course of treatment. She stopped chemotherapy permanently after 2 cycles of cisplatin, since her CT scan showed progression and her CA-125 was rising (thought ctDNA falling). She was discharged from clinic with best supportive care. She went on to live 238 days (7 months) without any further treatment, indicating that the fall in ctDNA may have been a better predictor of outcome that the CT or CA-125. Of note her pre-treatment CT scan was 5 weeks before the start of treatment, and therefore the re-assessment CT scan carried out 8 weeks after the first cycle of chemotherapy could have overestimated progression on treatment.

OV04-70

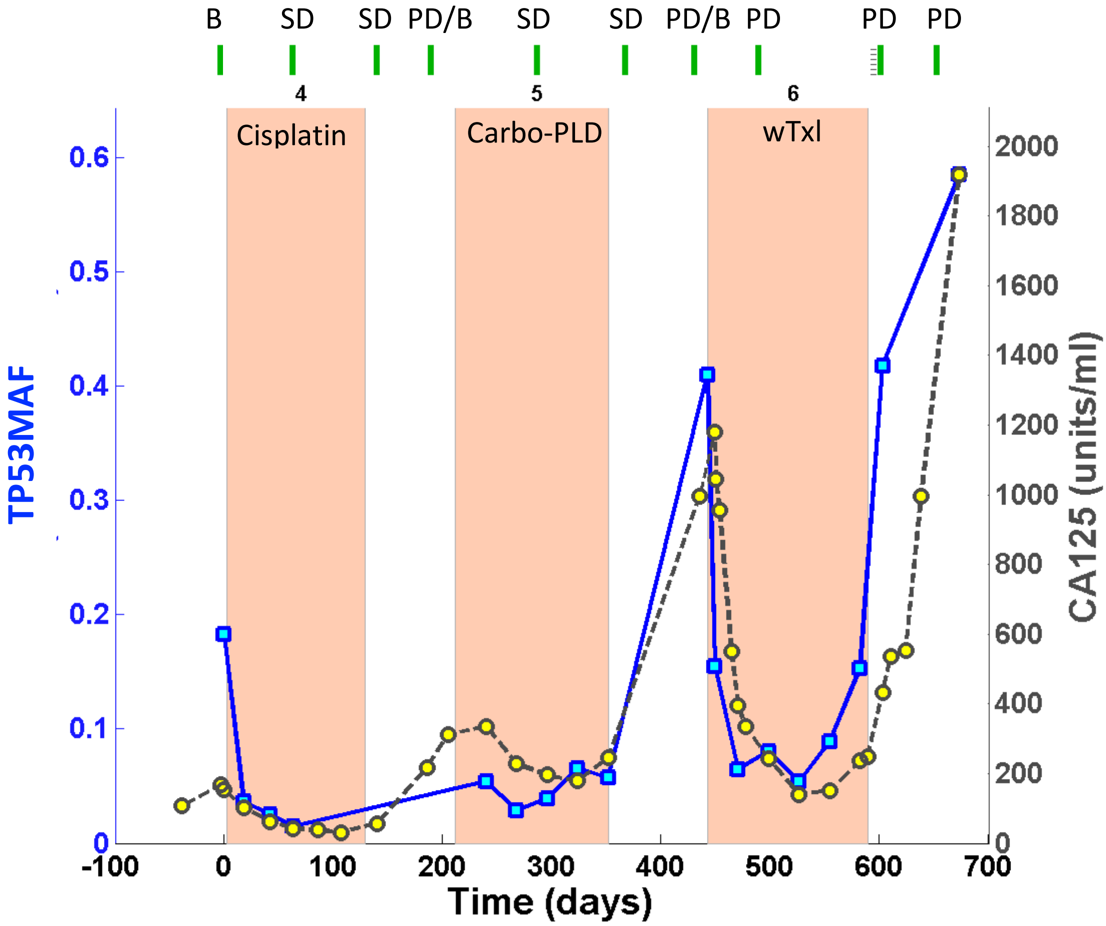

B
SD
SD
PD/B
SD
SD
PD/B
PD
PD
PD
4
5
6
Cisplatin
Carbo-PLD
wTxl
TP53MAF
CA125 (units/ml)
Time (days)
0
0.1
0.2
0.3
0.4
0.5
0.6
0
200
400
600
800
1000
1200
1400
1600
1800
2000
-100
0
100
200
300
400
500
600
700

OV04-72

Stage at diagnosis: IV
Residual disease: no residual

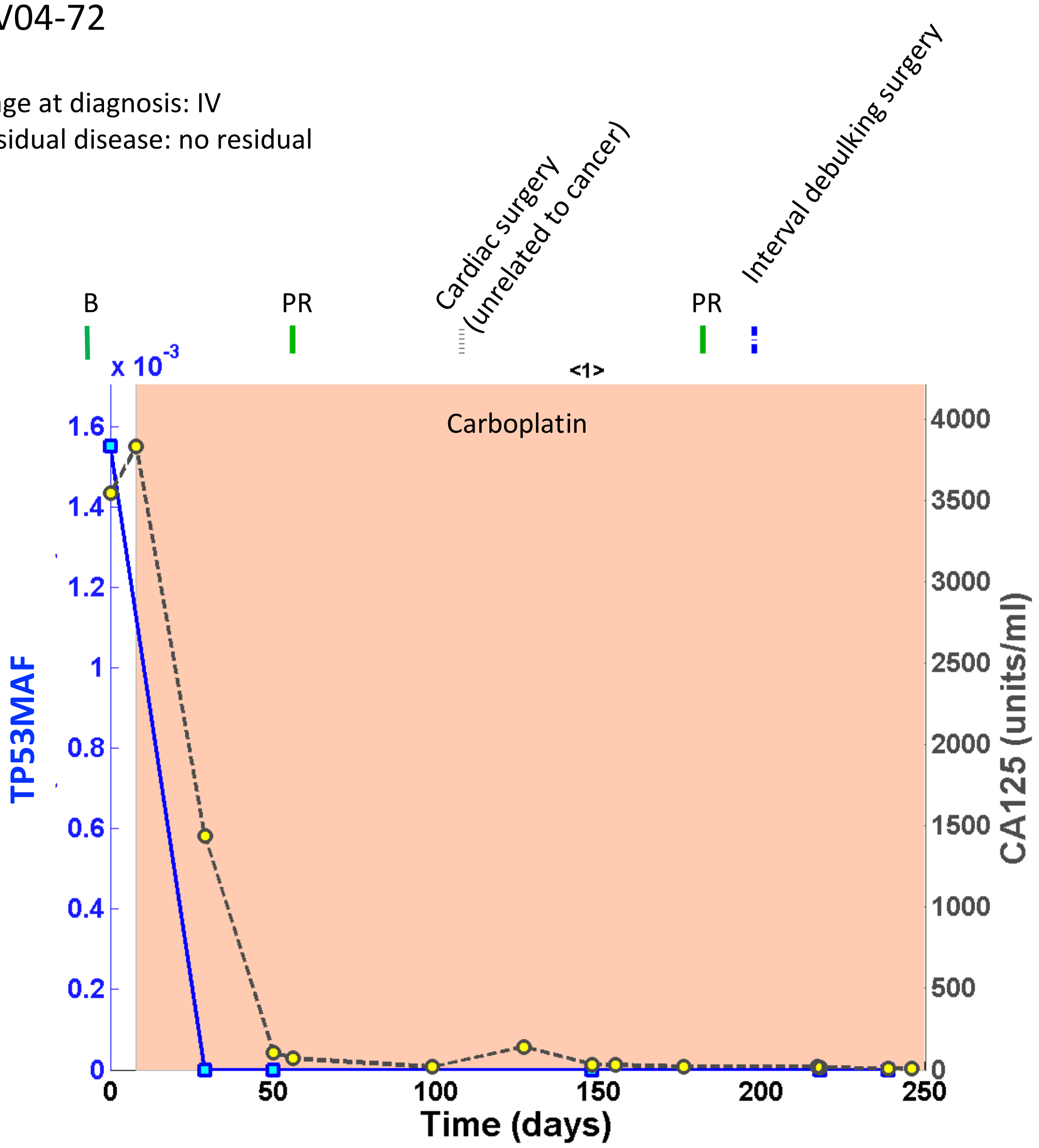

OV04-73

Stage at diagnosis: IIIC
Residual disease: NA (primary chemotherapy)

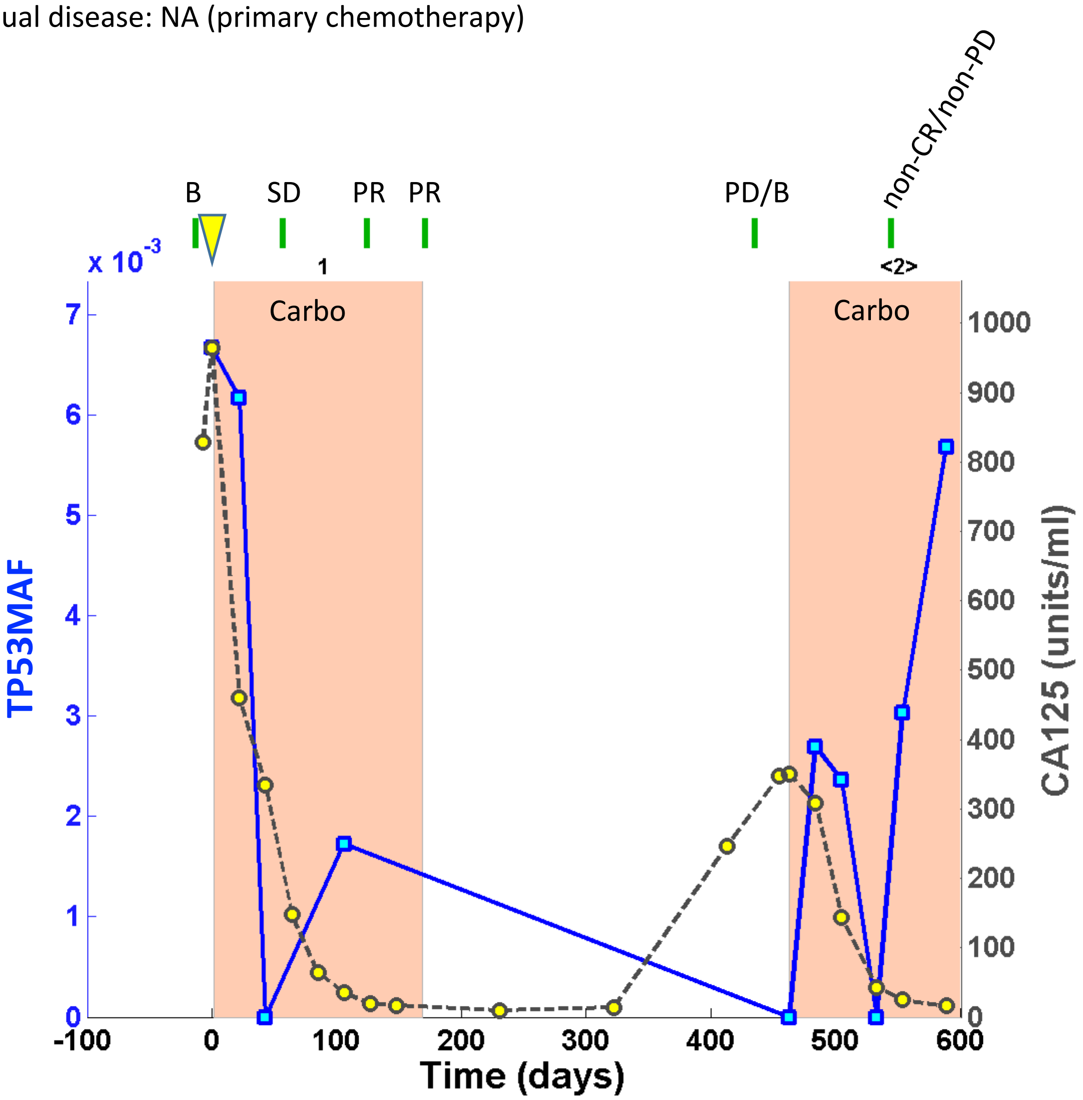

OV04-74

Stage at diagnosis: IIIC
Residual disease: no residual

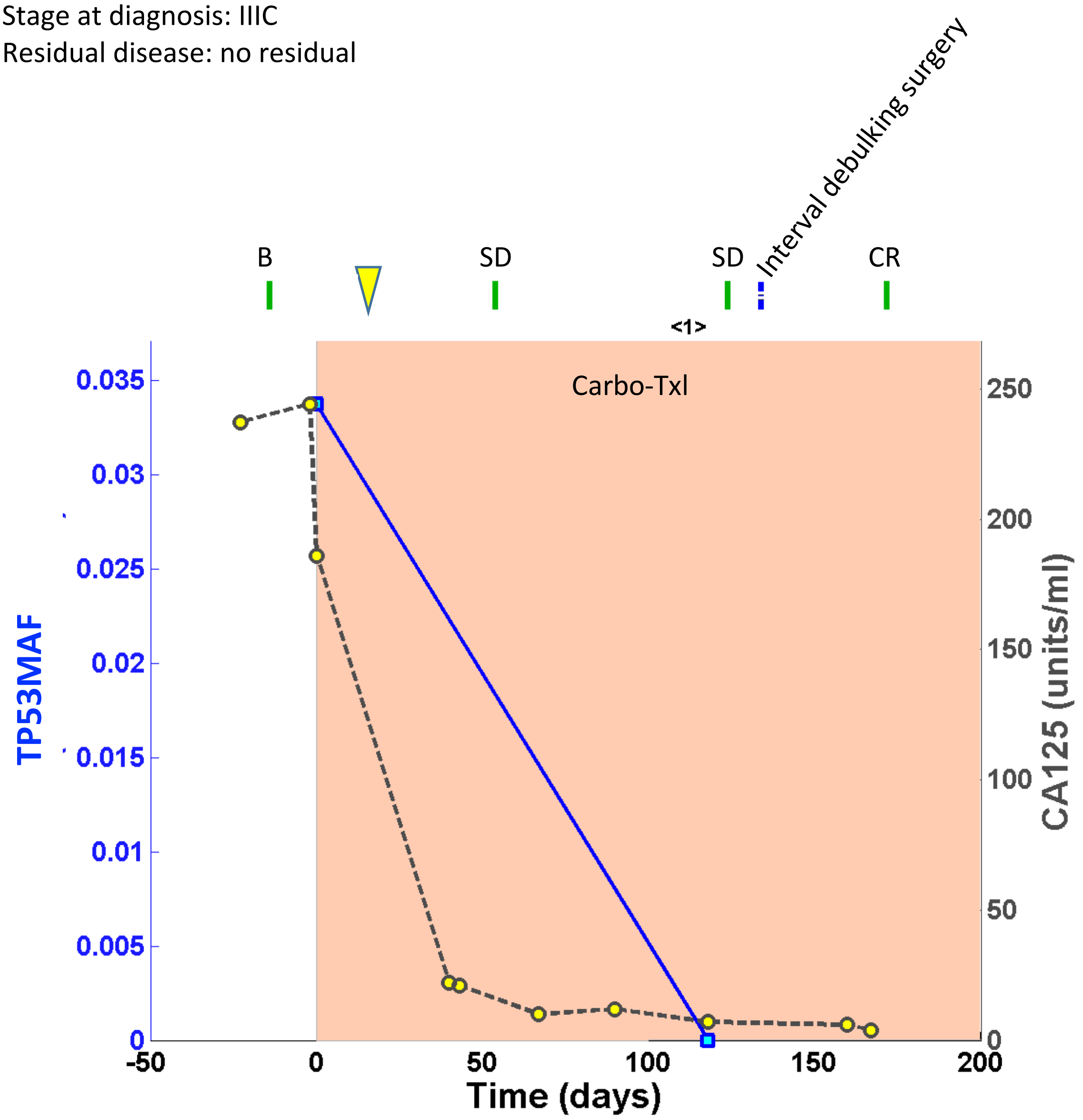

OV04-75

Stage at diagnosis: IIIC
Residual disease: no residual

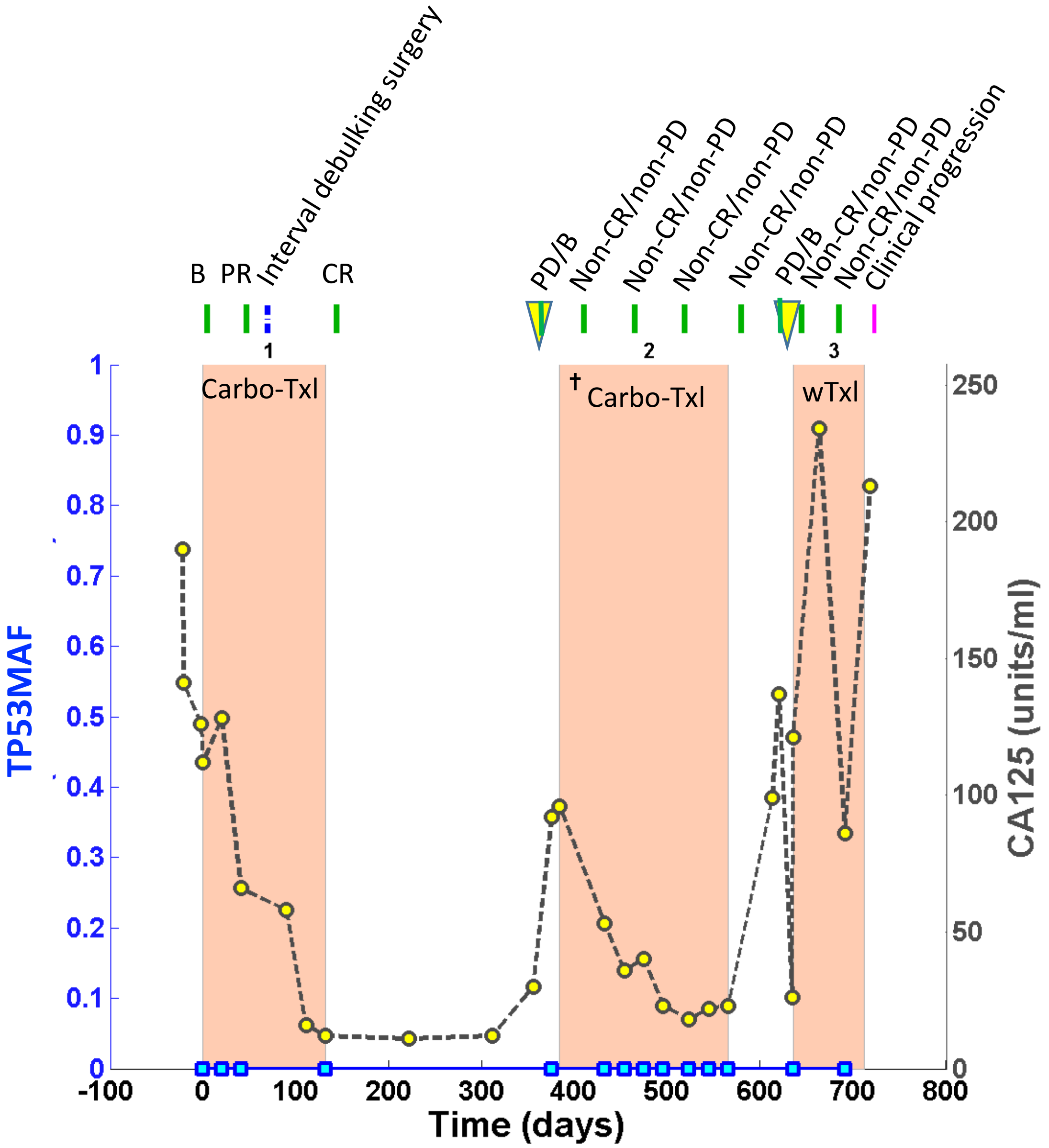

† 1st cycle with Cediranib/placebo on ICON6 trial (Cedirinib/placebo stopped due to toxicity).

OV04-76

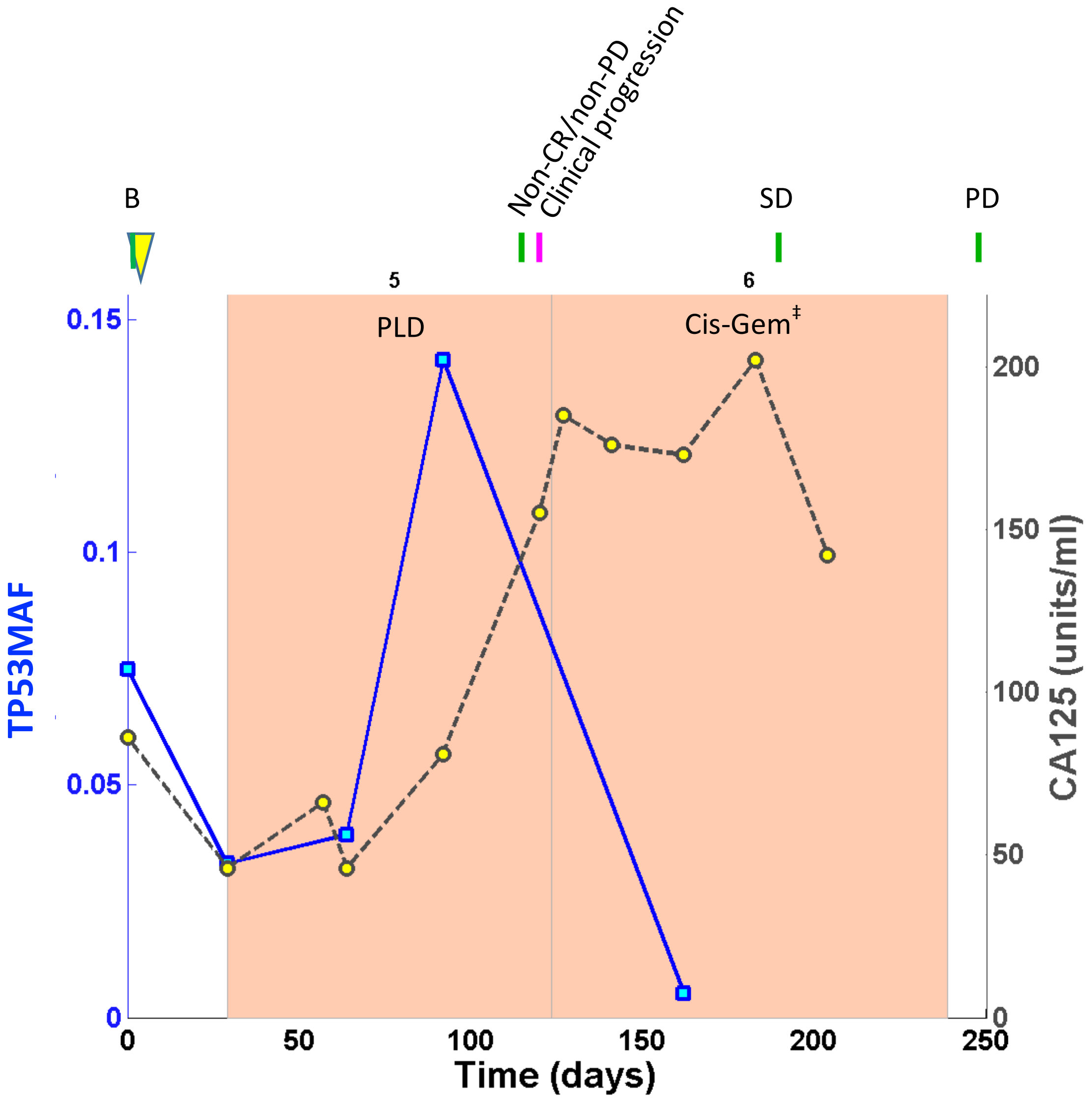

‡ Gemcitabine stopped for last 4 cycles (of total of 6 cycles)

OV04-79

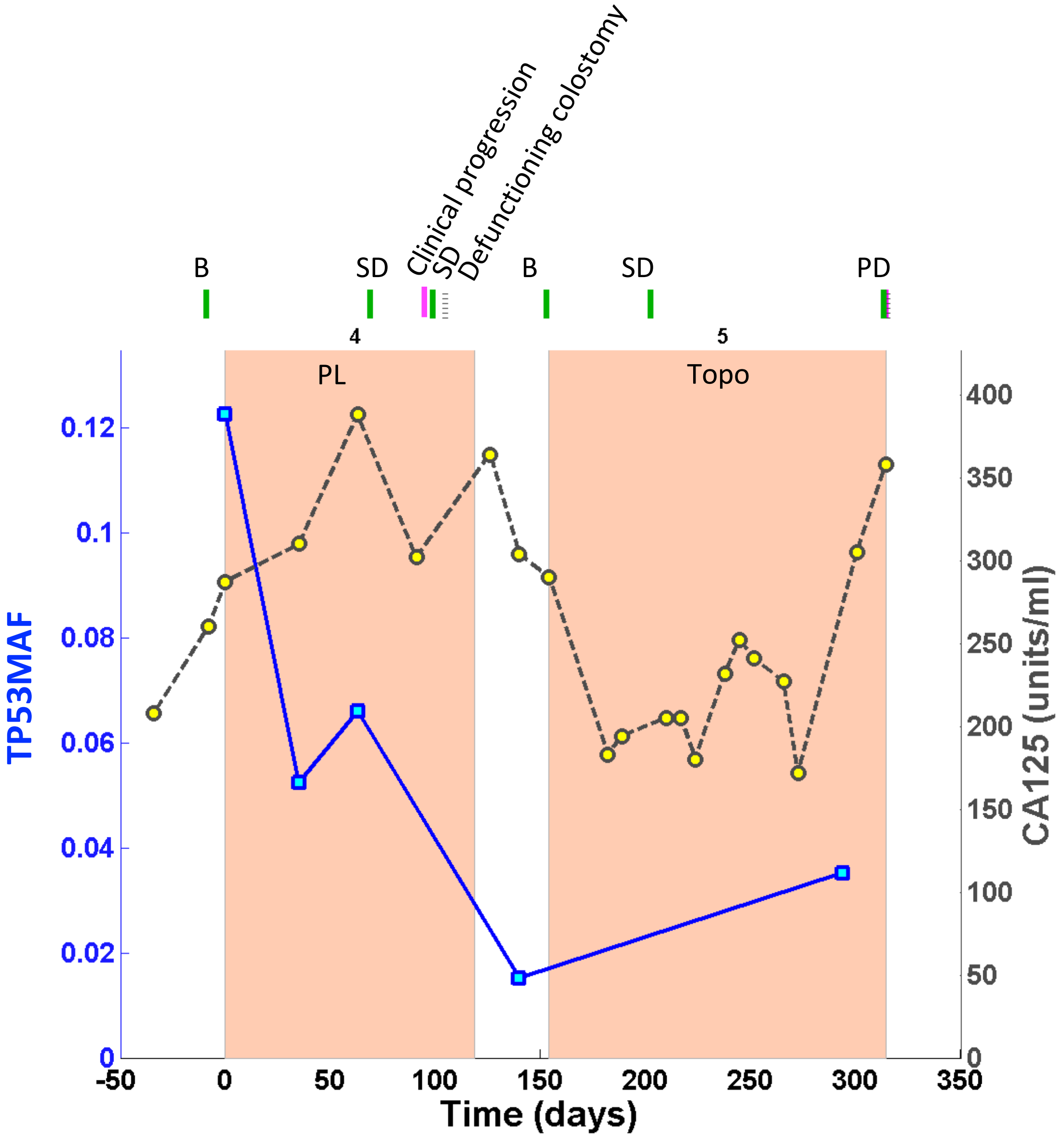

Clinical progression
SD
Defunctioning colostomy
B
SD
B
SD
PD
4
5
PL
Topo
TP53MAF
CA125 (units/ml)
Time (days)
0.12
0.1
0.08
0.06
0.04
0.02
0
400
350
300
250
200
150
100
50
0
-50
0
50
100
150
200
250
300
350

OV04-81

Stage at diagnosis: IIIC
Residual disease: >1 cm

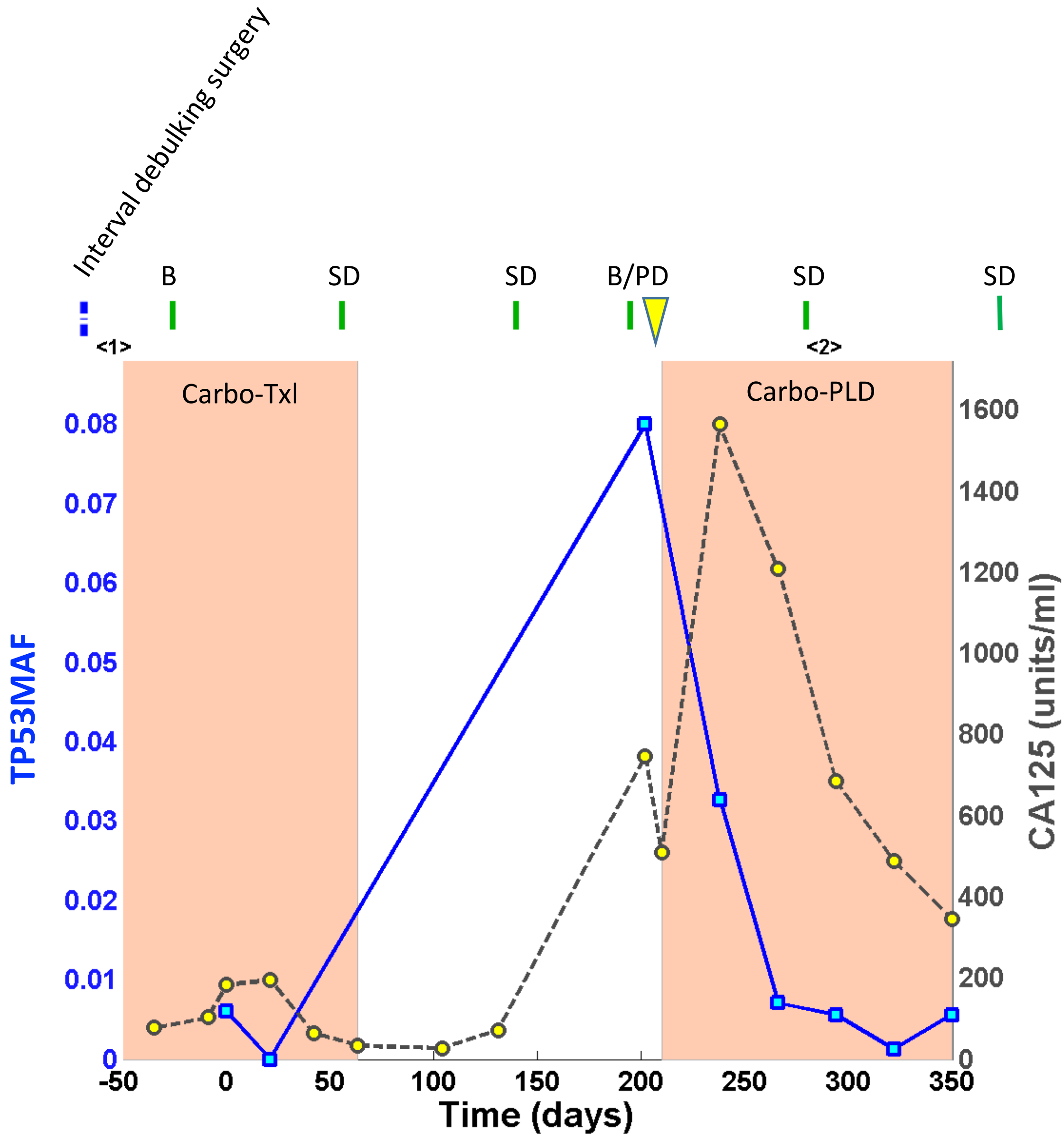

OV04-90

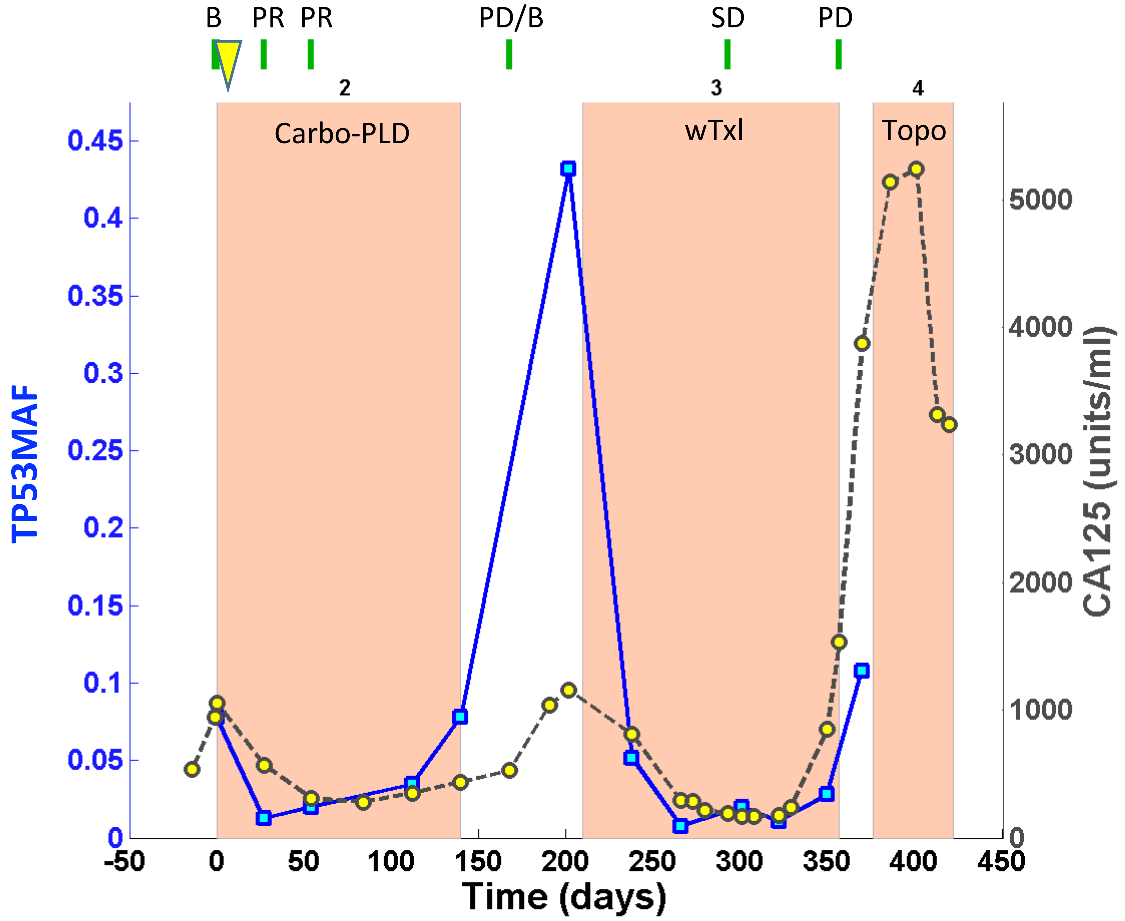

B
PR
PR
PD/B
SD
PD
2
3
4
Carbo-PLD
wTxl
Topo
TP53MAF
0.45
0.4
0.35
0.3
0.25
0.2
0.15
0.1
0.05
0
CA125 (units/ml)
5000
4000
3000
2000
1000
0
-50
0
50
100
150
200
250
300
350
400
450
Time (days)

OV04-95

Stage at diagnosis: IC
Residual disease: no residual

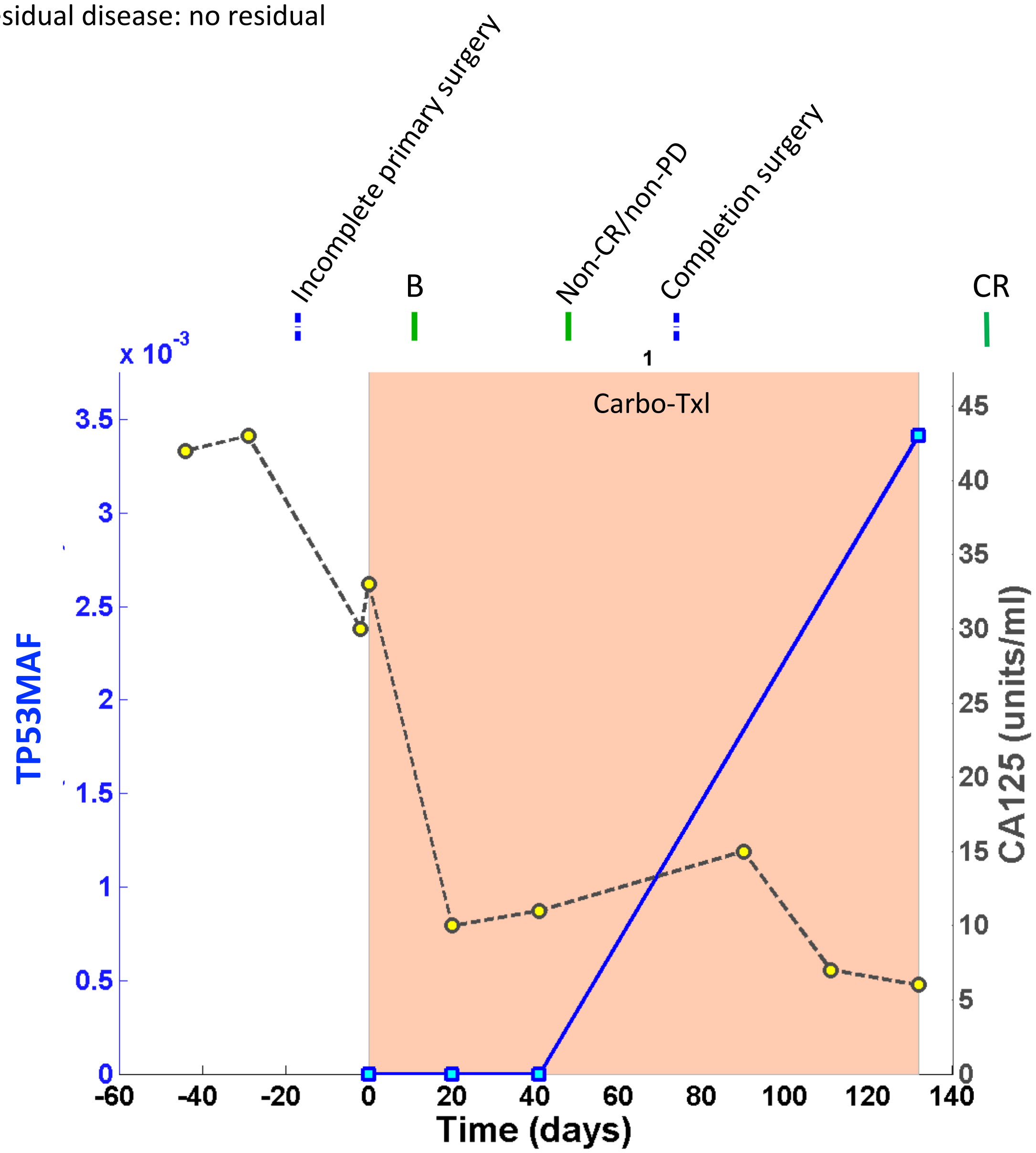

OV04-97

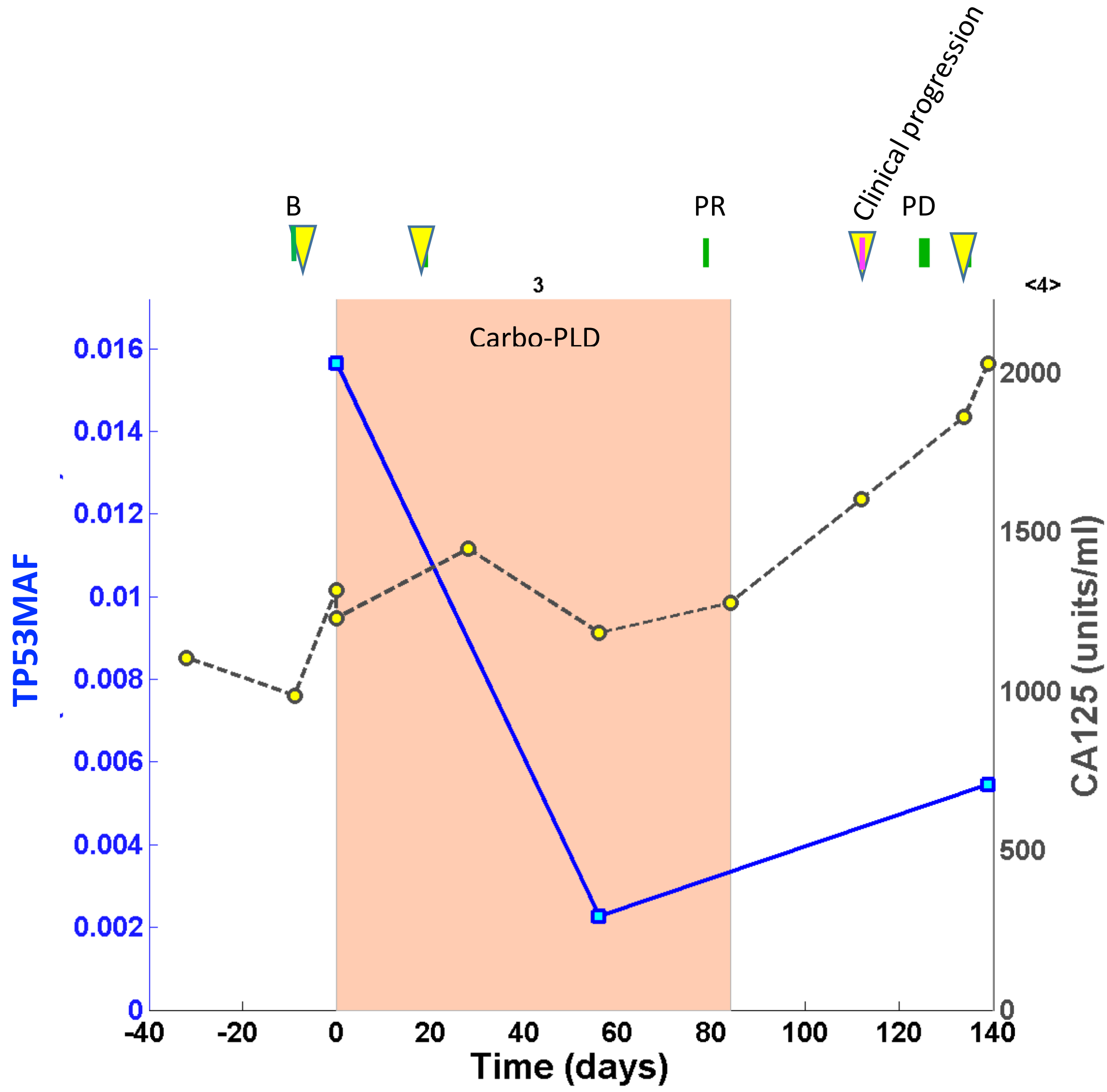

Clinical progression
B
PR
PD
3
<4>
Carbo-PLD
TP53MAF
0.016
0.014
0.012
0.01
0.008
0.006
0.004
0.002
0
2000
1500
1000
500
0
CA125 (units/ml)
-40
-20
0
20
40
60
80
100
120
140
Time (days)

OV04-98

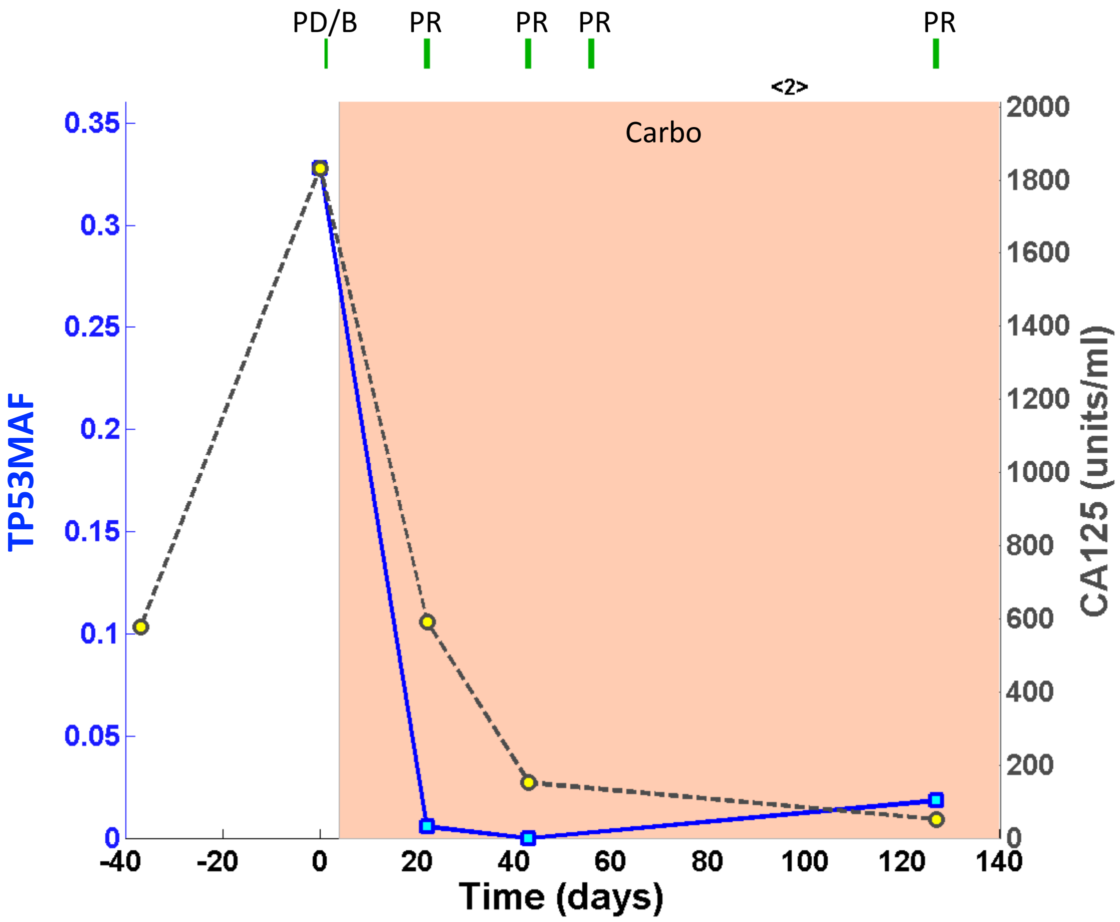
PD/B
PR
PR
PR
PR
<2>
Carbo
TP53MAF
0.35
0.3
0.25
0.2
0.15
0.1
0.05
0
-40
-20
0
20
40
60
80
100
120
140
Time (days)
2000
1800
1600
1400
1200
1000
800
600
400
200
0
CA125 (units/ml)

OV04-102

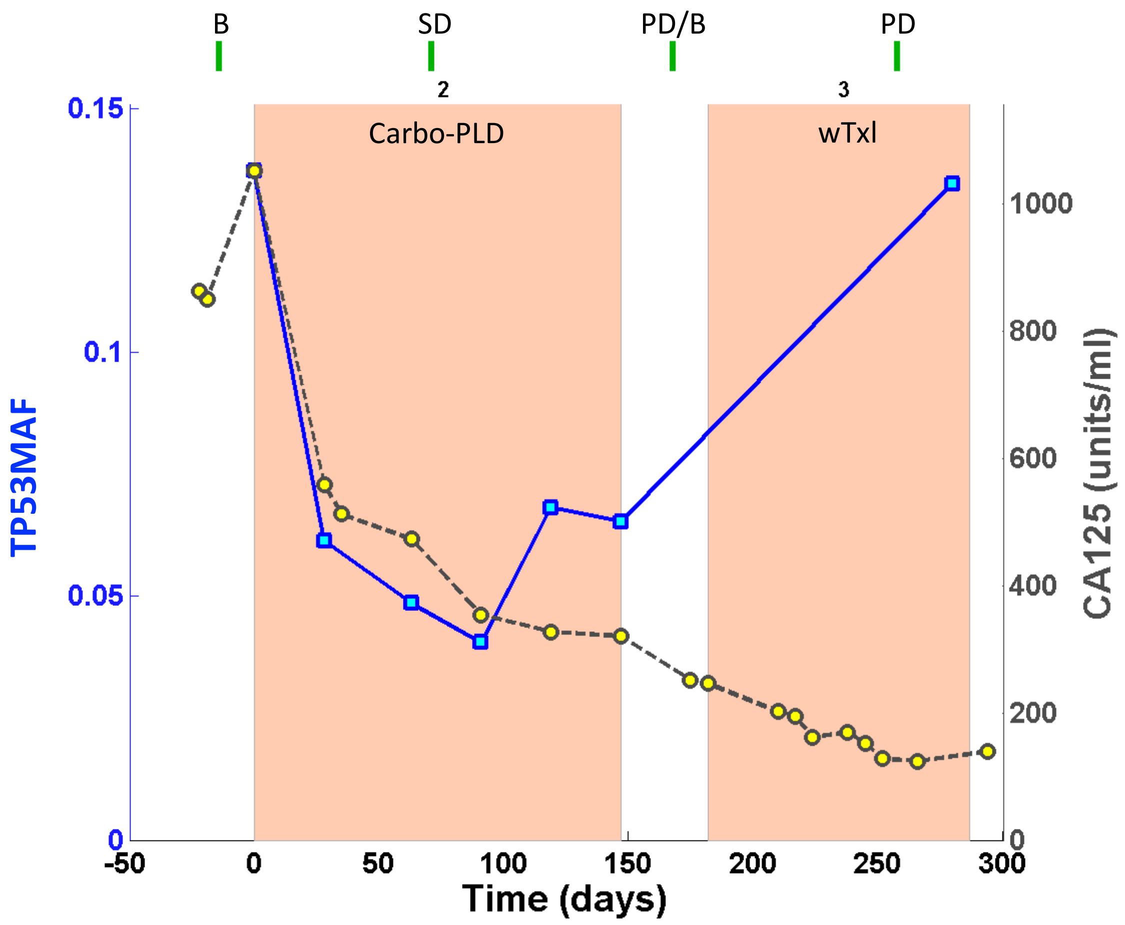

B
SD
PD/B
PD
2
3
Carbo-PLD
wTxl
0.15
0.1
0.05
0
TP53MAF
1000
800
600
400
200
0
CA125 (units/ml)
-50
0
50
100
150
200
250
300
Time (days)

OV04-105

Stage at diagnosis: IIIC
Residual disease: >1 cm

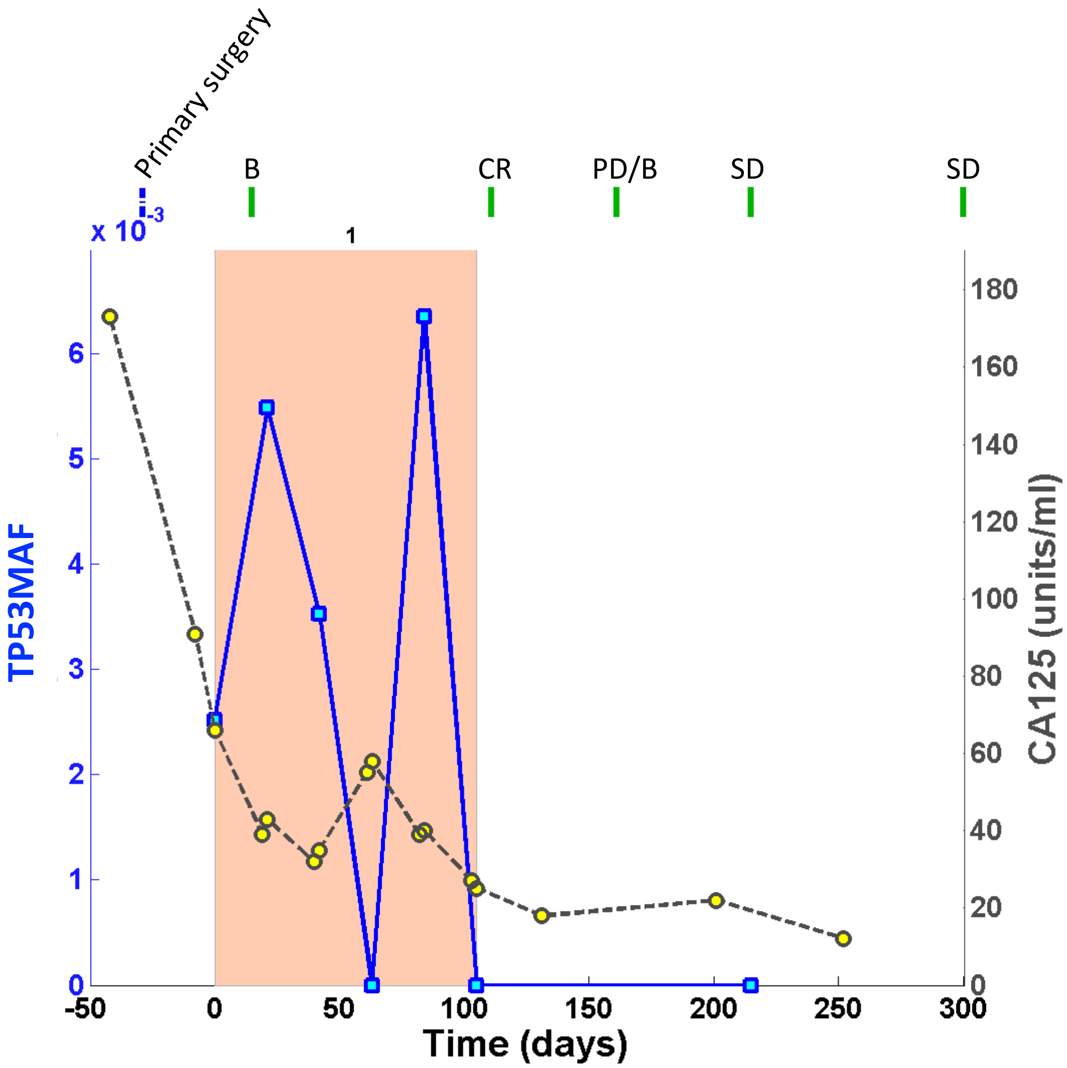

OV04-112

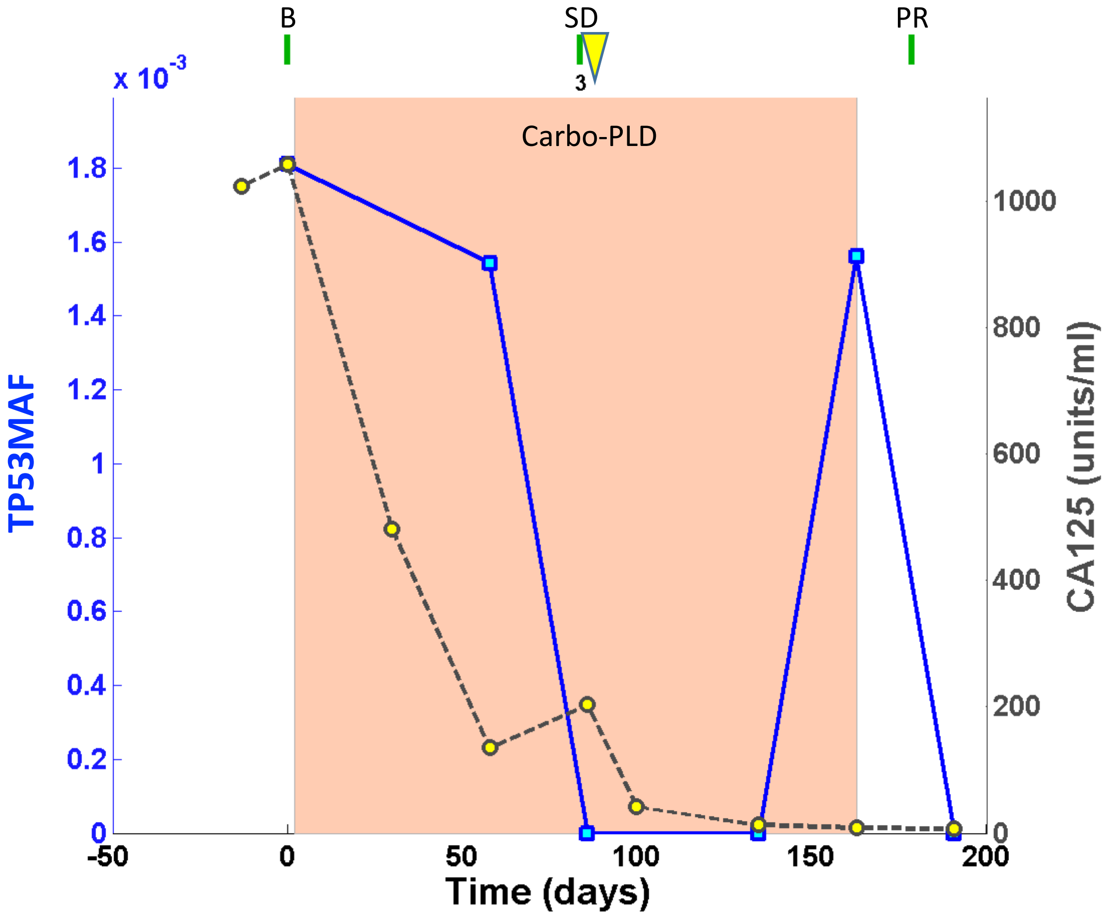

B
SD
3
PR
x 10^-3
Carbo-PLD
TP53MAF
CA125 (units/ml)
Time (days)
1.8
1.6
1.4
1.2
1
0.8
0.6
0.4
0.2
0
1000
800
600
400
200
0
-50
0
50
100
150
200

OV04-114

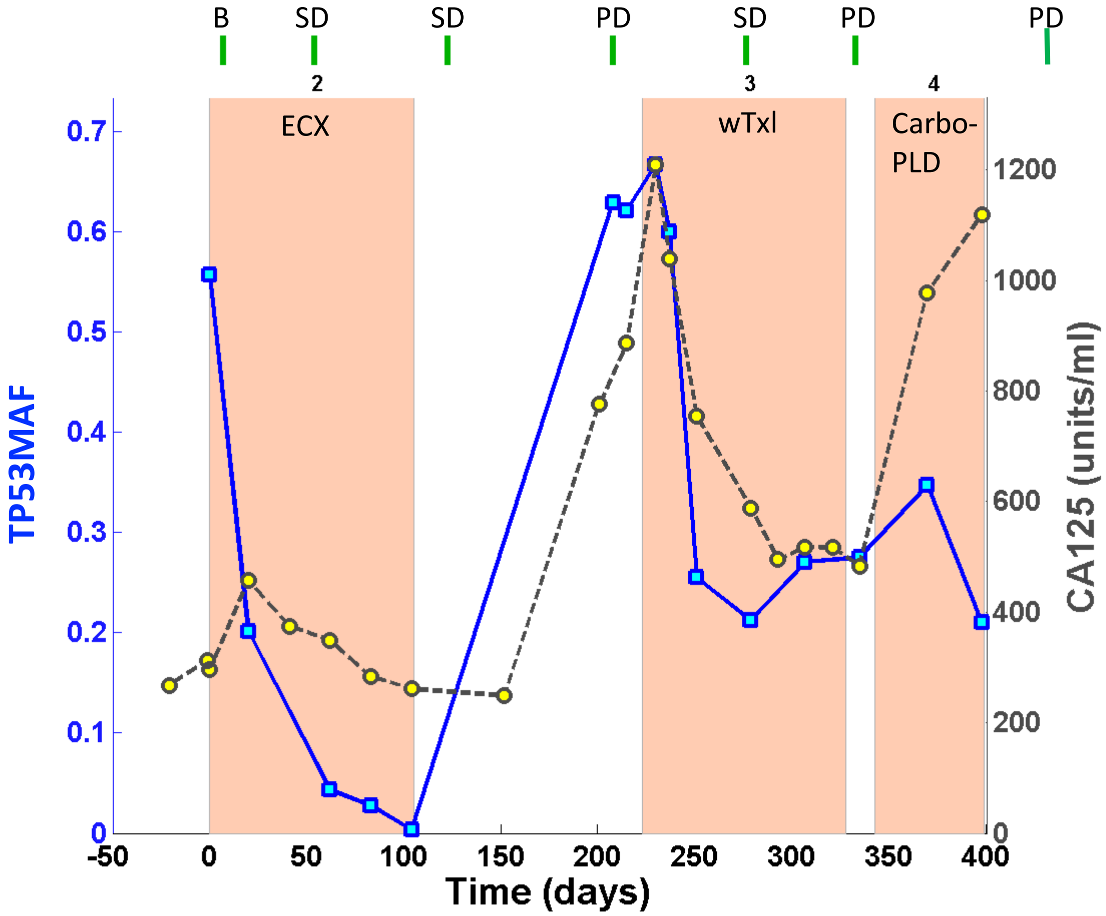
B
SD
SD
PD
SD
PD
PD
2
3
4
ECX
wTxl
Carbo-
PLD
TP53MAF
CA125 (units/ml)
Time (days)
0.7
0.6
0.5
0.4
0.3
0.2
0.1
0
1200
1000
800
600
400
200
0
-50
0
50
100
150
200
250
300
350
400

OV04-116

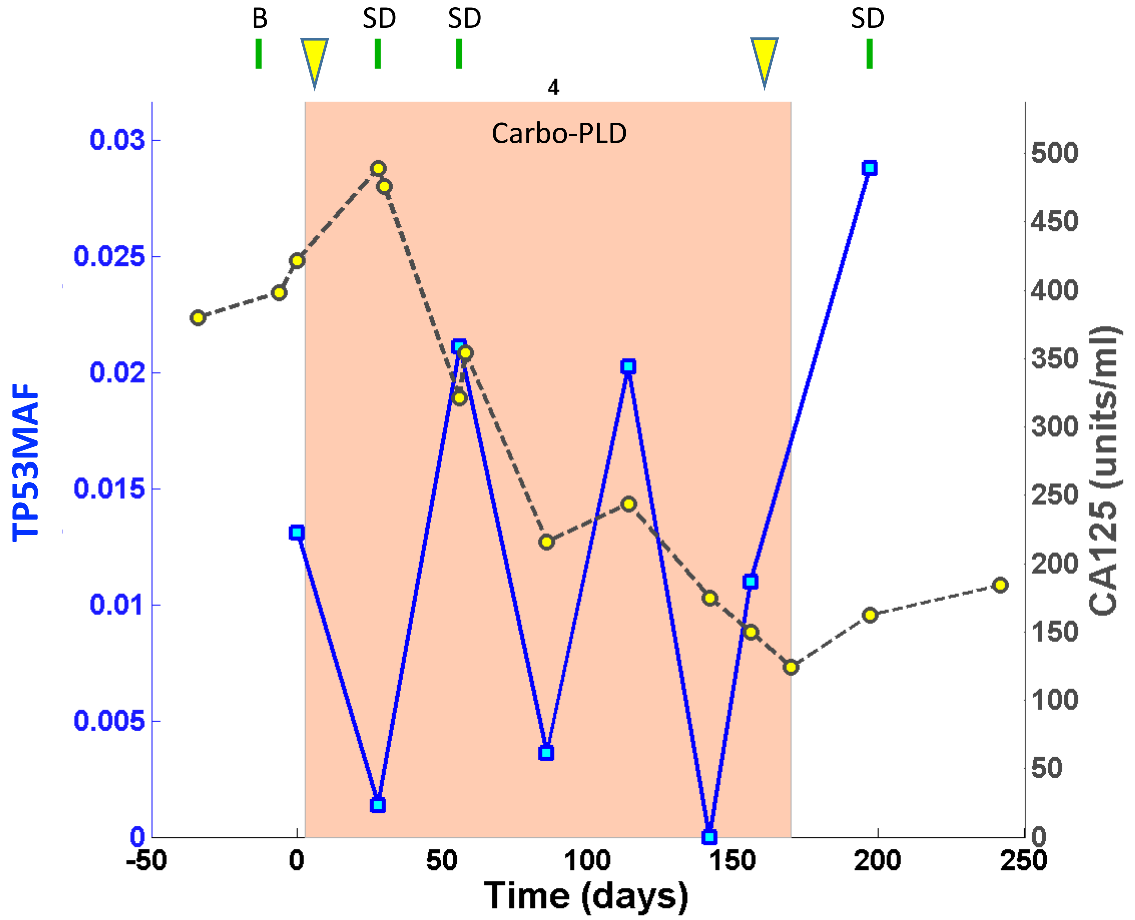

B
SD
SD
SD
4
Carbo-PLD
TP53MAF
CA125 (units/ml)
Time (days)
0.03
0.025
0.02
0.015
0.01
0.005
0
-50
0
50
100
150
200
250
500
450
400
350
300
250
200
150
100
50
0

OV04-127

Stage at diagnosis: IIIC
Residual disease: no residual

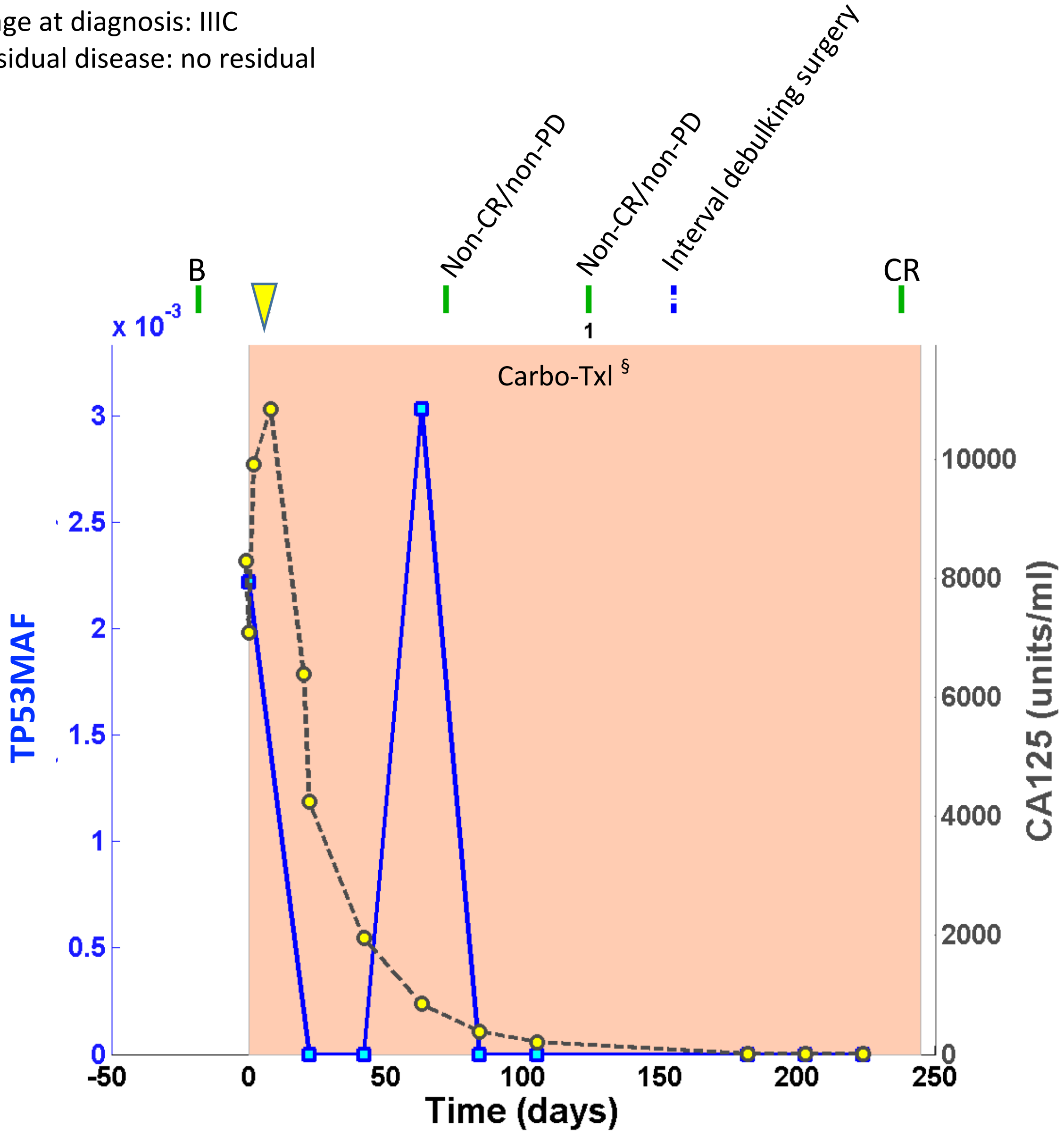

§ 1st 4 cycles carboplatin, last 5 cycles carboplatin and paclitaxel

OV04-129

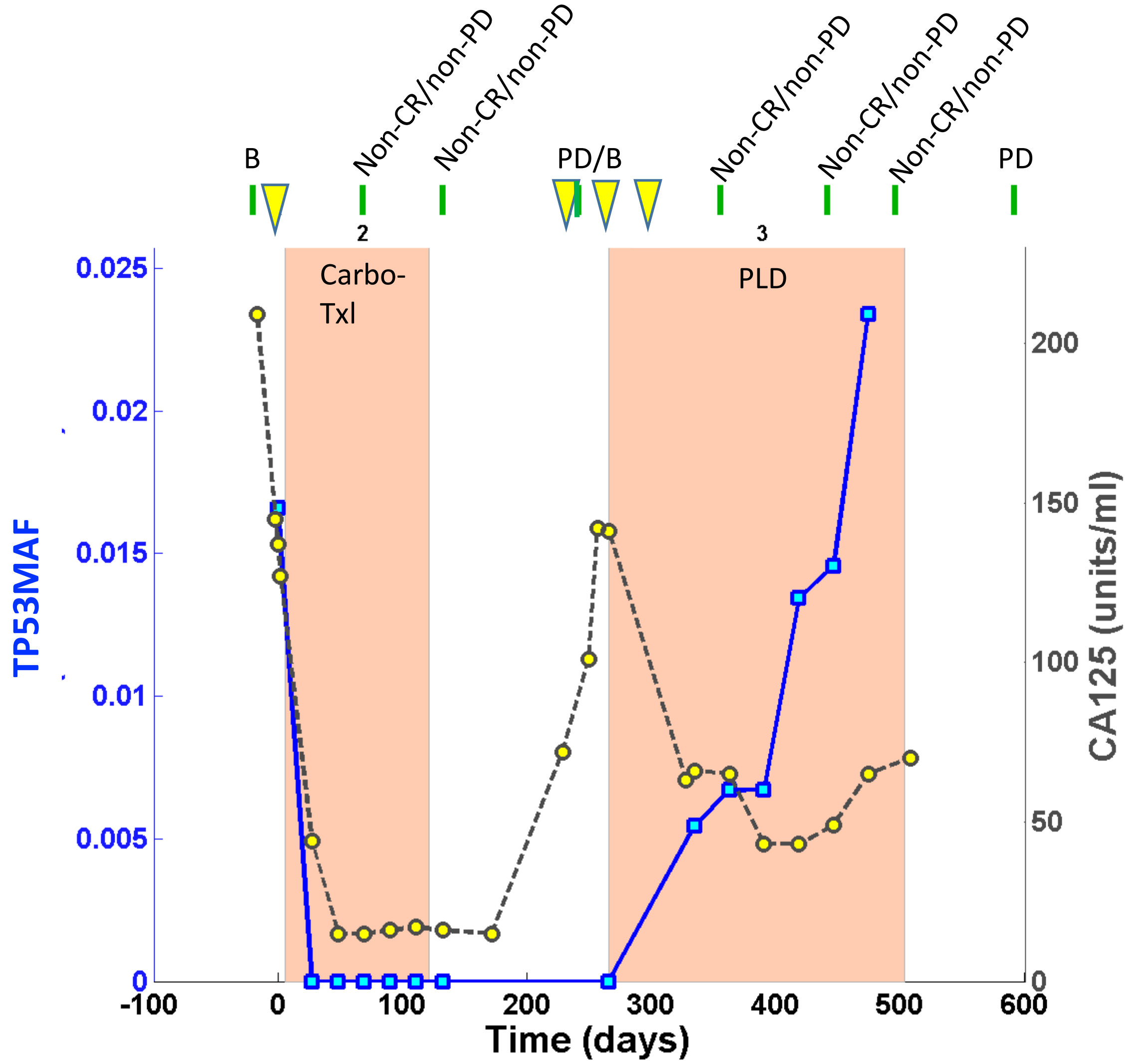

B
Non-CR/non-PD
Non-CR/non-PD
PD/B
Non-CR/non-PD
Non-CR/non-PD
Non-CR/non-PD
PD
2
3
Carbo-
Txl
PLD
TP53MAF
0.025
0.02
0.015
0.01
0.005
0
200
150
100
50
0
CA125 (units/ml)
-100
0
100
200
300
400
500
600
Time (days)

OV04-133

Stage at diagnosis: IC
Residual disease: no residual

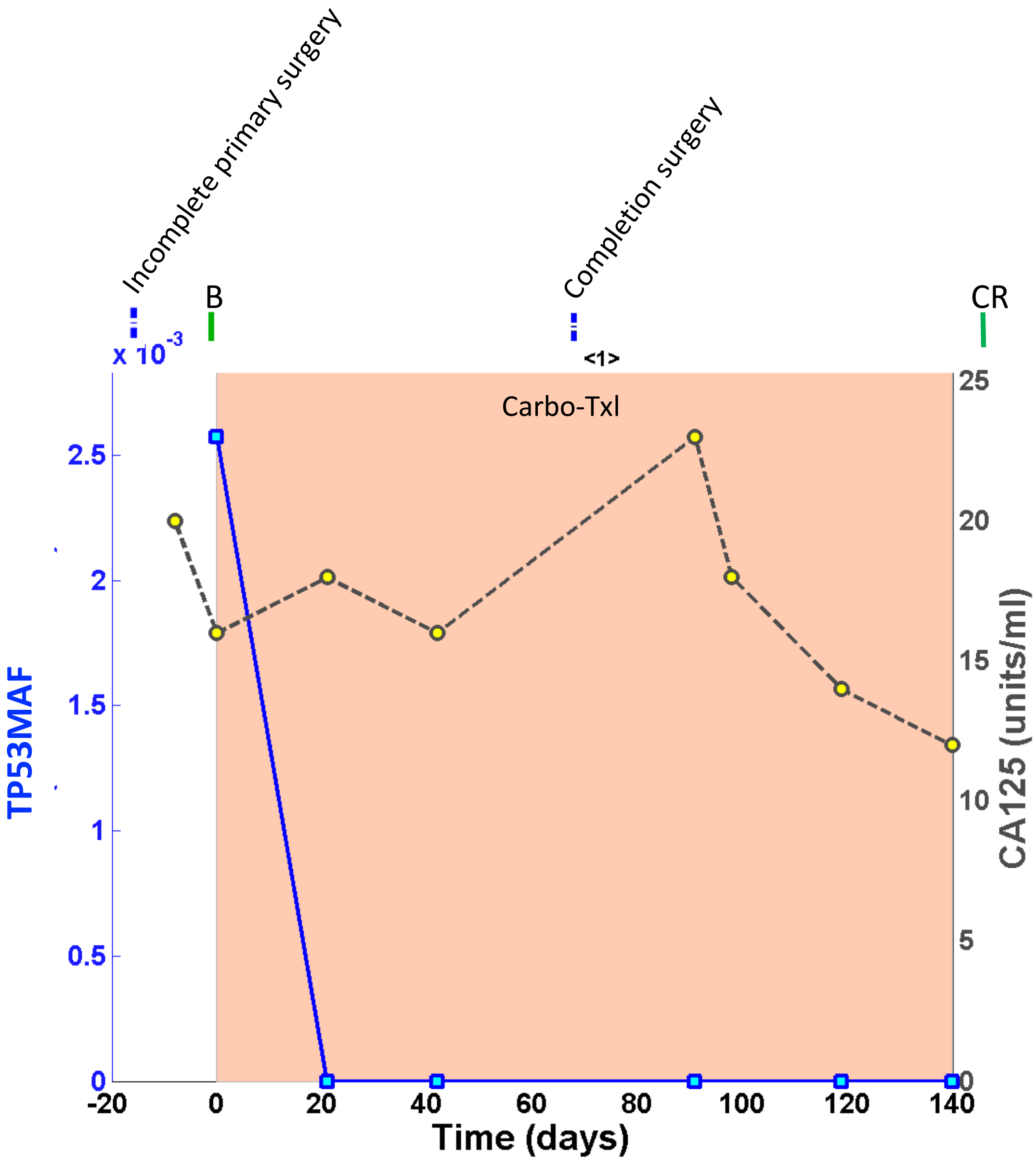

OV04-135

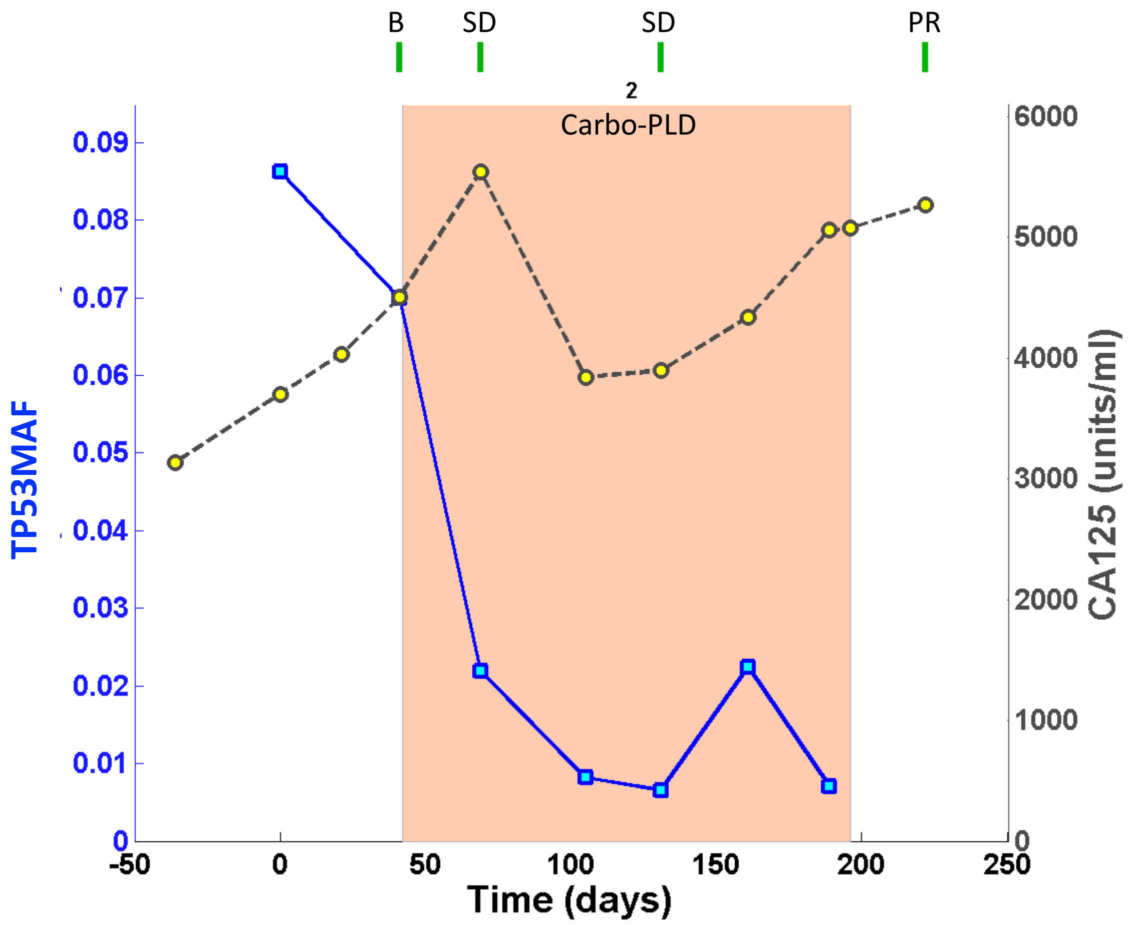
B
SD
SD
PR
2
Carbo-PLD
TP53MAF
0.09
0.08
0.07
0.06
0.05
0.04
0.03
0.02
0.01
0
6000
5000
4000
3000
2000
1000
0
CA125 (units/ml)
-50
0
50
100
150
200
250
Time (days)

OV04-142

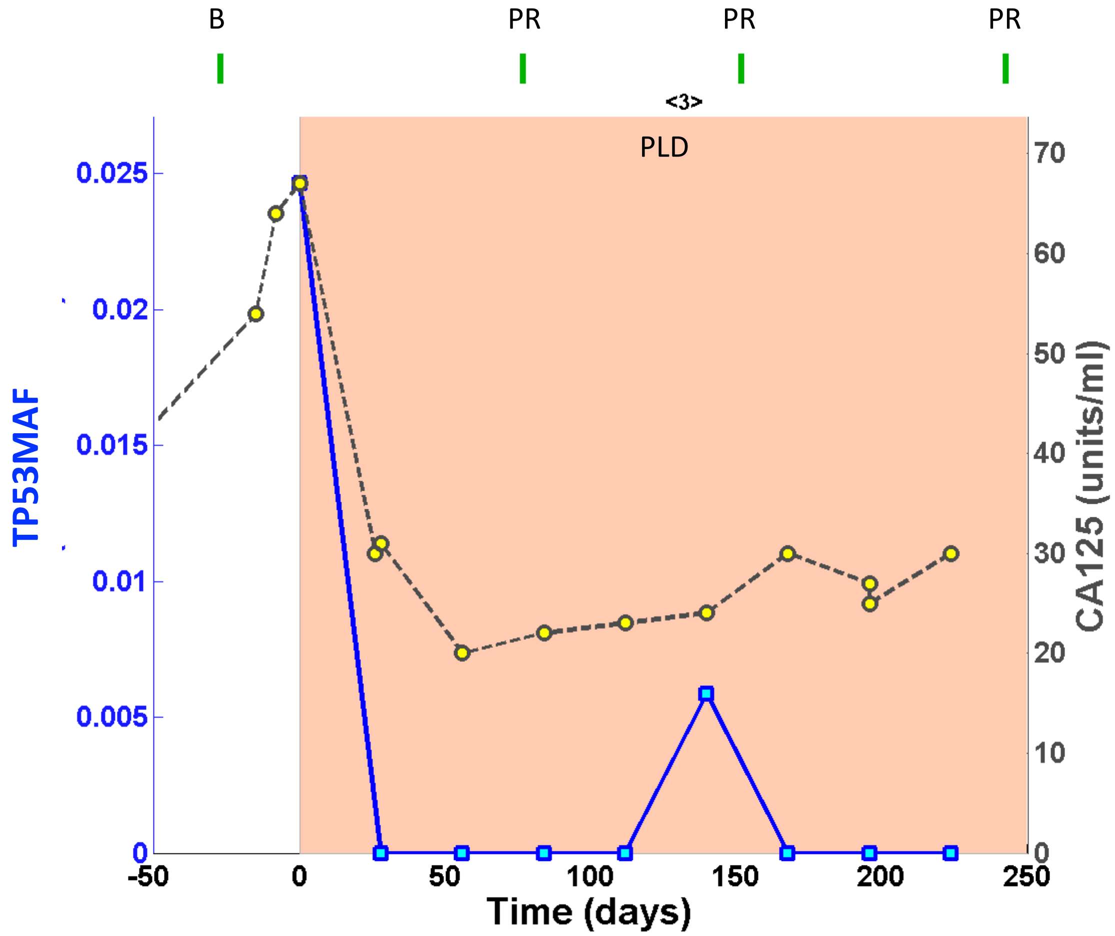
B
PR
PR
PR
<3>
PLD
TP53MAF
0.025
0.02
0.015
0.01
0.005
0
70
60
50
40
30
20
10
0
CA125 (units/ml)
-50
0
50
100
150
200
250
Time (days)

OV04-144

Stage at diagnosis: IIIC
Residual disease: <1 cm

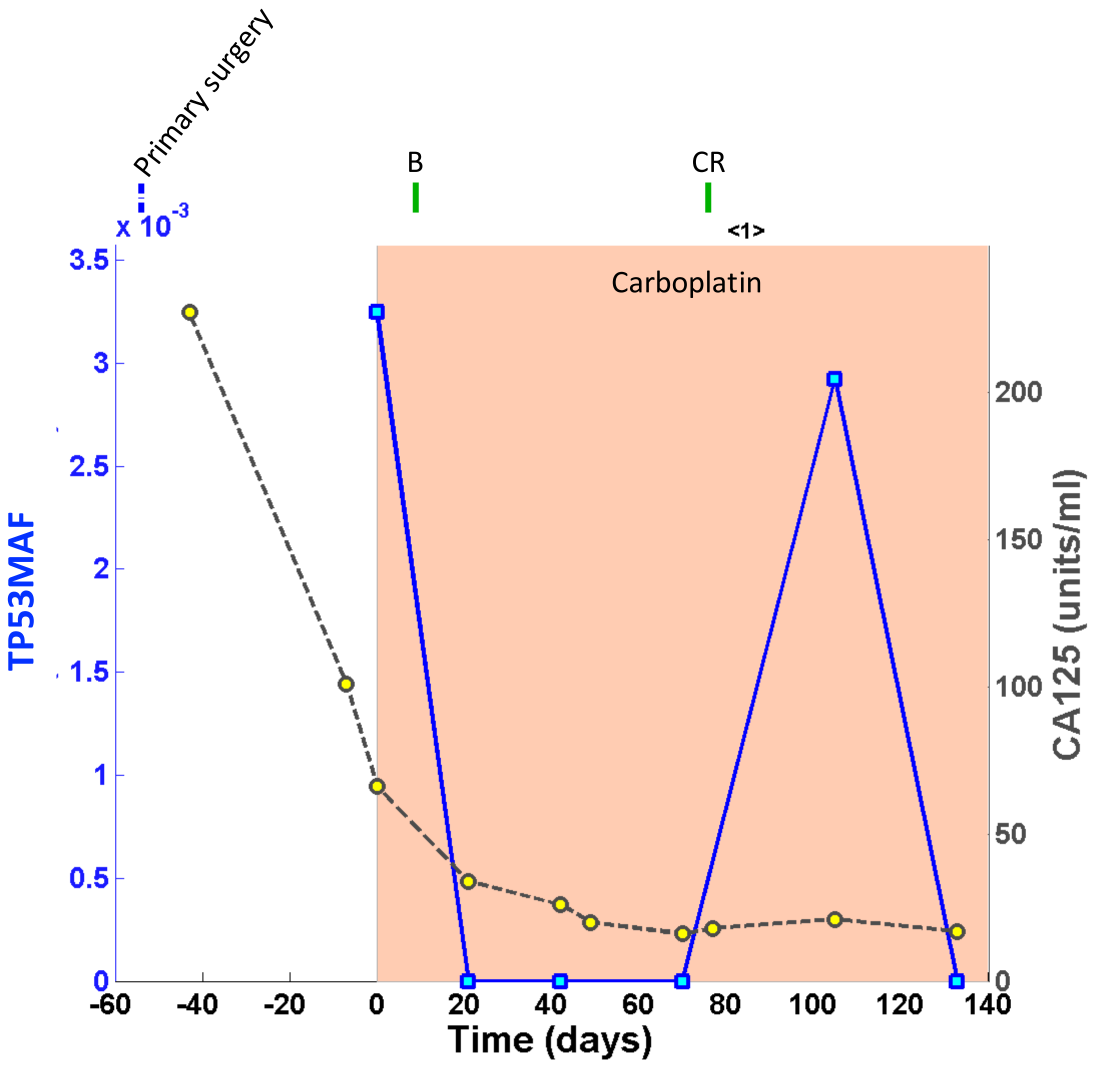

OV04-145

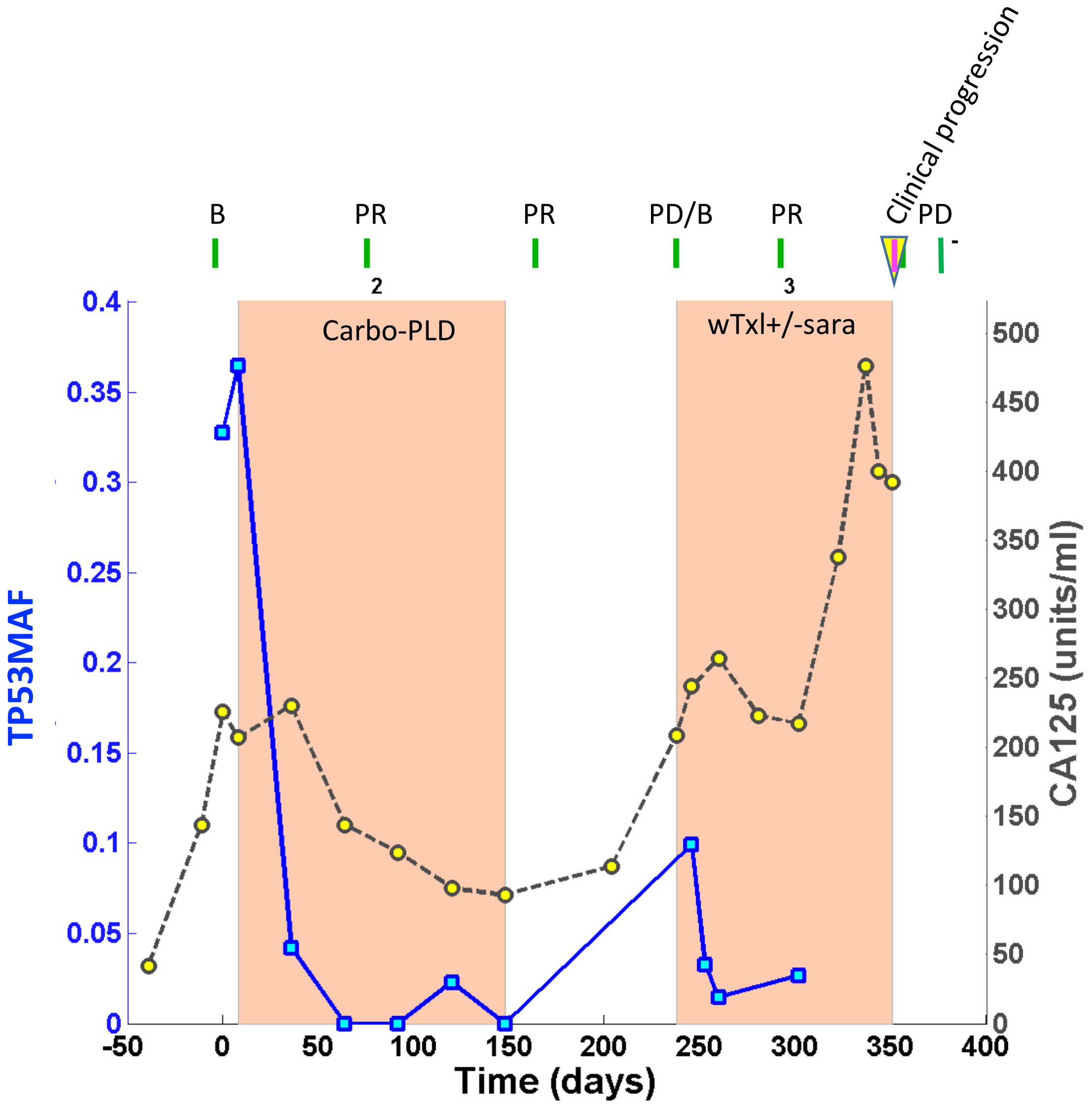

Clinical progression
B
PR
PR
PD/B
PR
PD
2
3
Carbo-PLD
wTxl+/-sara
TP53MAF
CA125 (units/ml)
Time (days)
0.4
0.35
0.3
0.25
0.2
0.15
0.1
0.05
0
-50
0
50
100
150
200
250
300
350
400
500
450
400
350
300
250
200
150
100
50
0

OV04-150

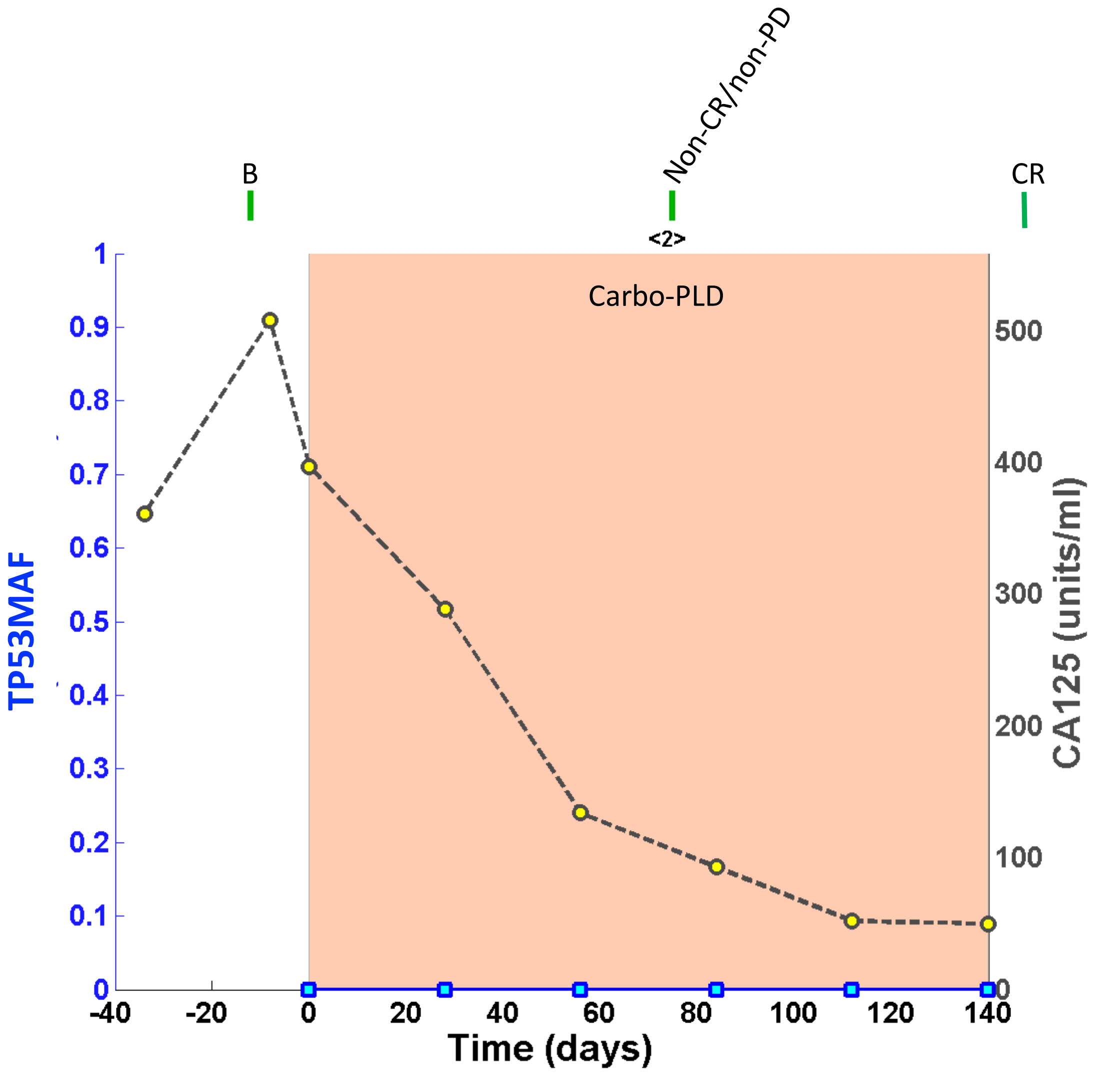
B
Non-CR/non-PD
CR
<2>
Carbo-PLD
TP53MAF
CA125 (units/ml)
Time (days)
1
0.9
0.8
0.7
0.6
0.5
0.4
0.3
0.2
0.1
0
-40
-20
0
20
40
60
80
100
120
140
500
400
300
200
100
0

OV04-161

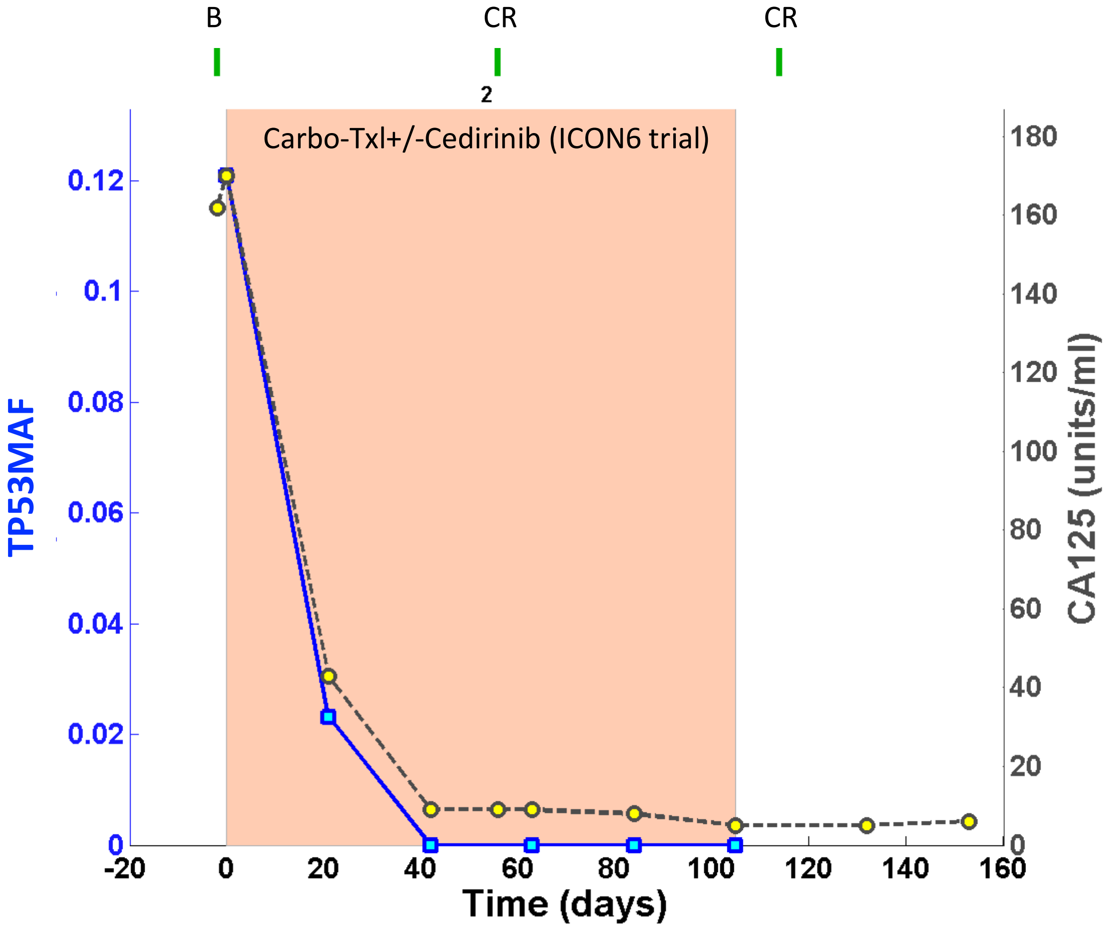
B
CR
CR
2
Carbo-Txl+/-Cedirinib (ICON6 trial)
TP53MAF
0.12
0.1
0.08
0.06
0.04
0.02
0
CA125 (units/ml)
180
160
140
120
100
80
60
40
20
0
-20
0
20
40
60
80
100
120
140
160
Time (days)

OV04-200

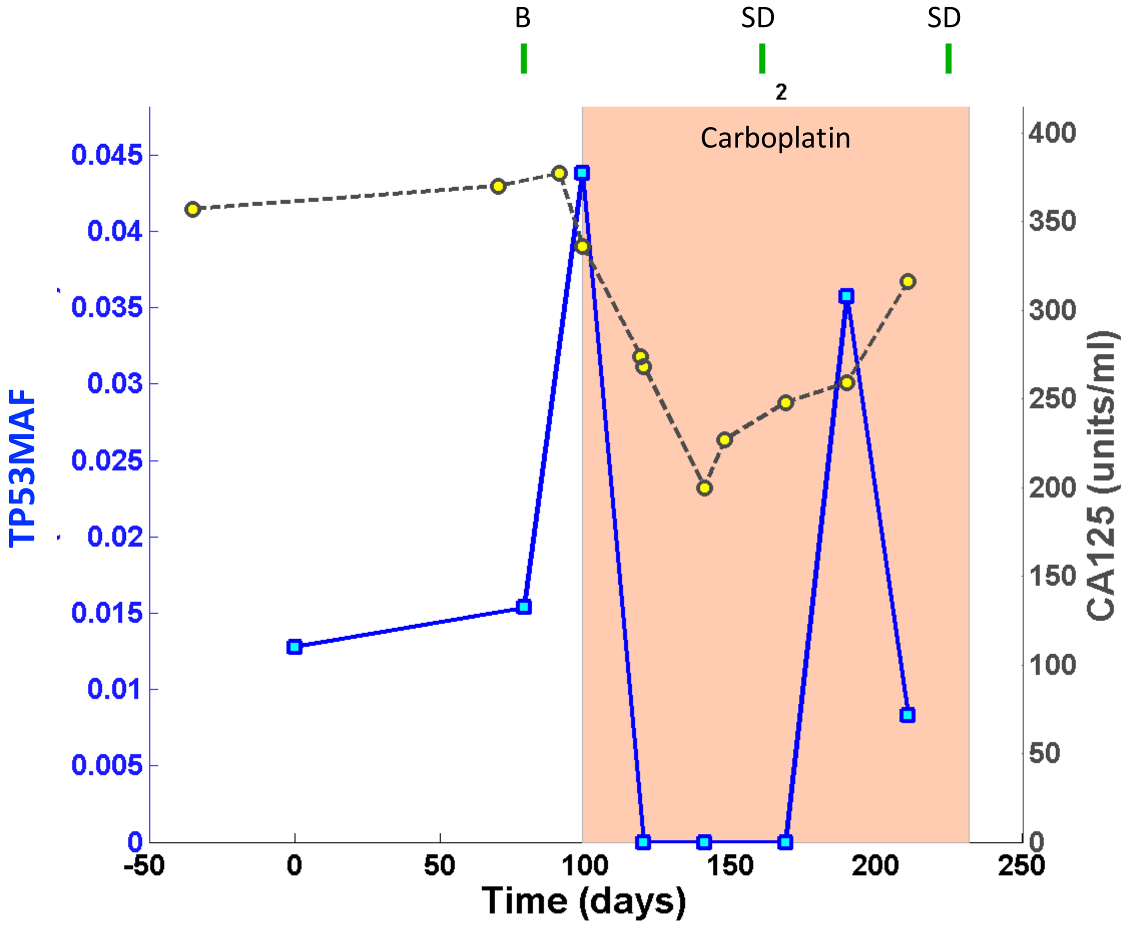

Supplement: S3 Fig — (PDF) [file pmed.1002198.s007.pdf]
