# Supplementary material for: Exploratory Analysis of TP53 Mutations in Circulating Tumour DNA as Biomarkers of Treatment Response for Patients with Relapsed High-Grade Serous Ovarian Carcinoma: A Retrospective Study
Source: PLoS Med. 2016 Dec 20;13(12):e1002198. doi: 10.1371/journal.pmed.1002198 (PMC5172526; doi:10.1371/journal.pmed.1002198)
Supplement: S4 Fig — (PPTX) [file pmed.1002198.s008.pptx]

## Slide 1
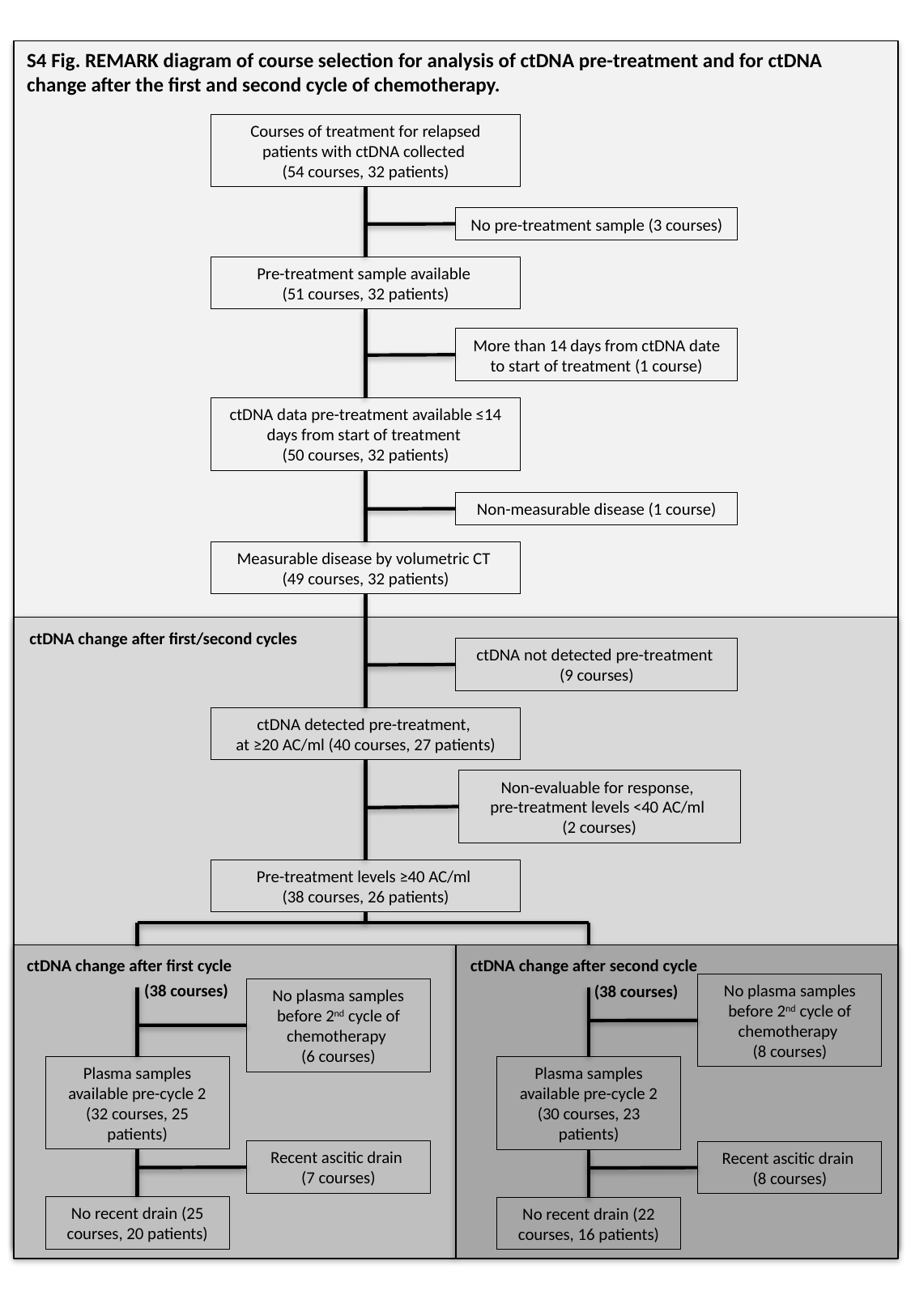

S4 Fig. REMARK diagram of course selection for analysis of ctDNA pre-treatment and for ctDNA change after the first and second cycle of chemotherapy.
Courses of treatment for relapsed patients with ctDNA collected
(54 courses, 32 patients)
No pre-treatment sample (3 courses)
Pre-treatment sample available
(51 courses, 32 patients)
More than 14 days from ctDNA date to start of treatment (1 course)
ctDNA data pre-treatment available ≤14 days from start of treatment
(50 courses, 32 patients)
Non-measurable disease (1 course)
Measurable disease by volumetric CT
(49 courses, 32 patients)
ctDNA change after first/second cycles
ctDNA not detected pre-treatment
(9 courses)
ctDNA detected pre-treatment,
at ≥20 AC/ml (40 courses, 27 patients)
Non-evaluable for response,
pre-treatment levels <40 AC/ml
(2 courses)
Pre-treatment levels ≥40 AC/ml
(38 courses, 26 patients)
ctDNA change after first cycle
ctDNA change after second cycle
(38 courses)
No plasma samples before 2nd cycle of chemotherapy
(8 courses)
(38 courses)
No plasma samples before 2nd cycle of chemotherapy
(6 courses)
Plasma samples available pre-cycle 2 (32 courses, 25 patients)
Plasma samples available pre-cycle 2 (30 courses, 23 patients)
Recent ascitic drain
(7 courses)
Recent ascitic drain
(8 courses)
No recent drain (25 courses, 20 patients)
No recent drain (22 courses, 16 patients)
