# Supplementary material for: Exploratory Analysis of TP53 Mutations in Circulating Tumour DNA as Biomarkers of Treatment Response for Patients with Relapsed High-Grade Serous Ovarian Carcinoma: A Retrospective Study
Source: PLoS Med. 2016 Dec 20;13(12):e1002198. doi: 10.1371/journal.pmed.1002198 (PMC5172526; doi:10.1371/journal.pmed.1002198)
Supplement: S5 Fig — (DOCX) [file pmed.1002198.s009.docx]

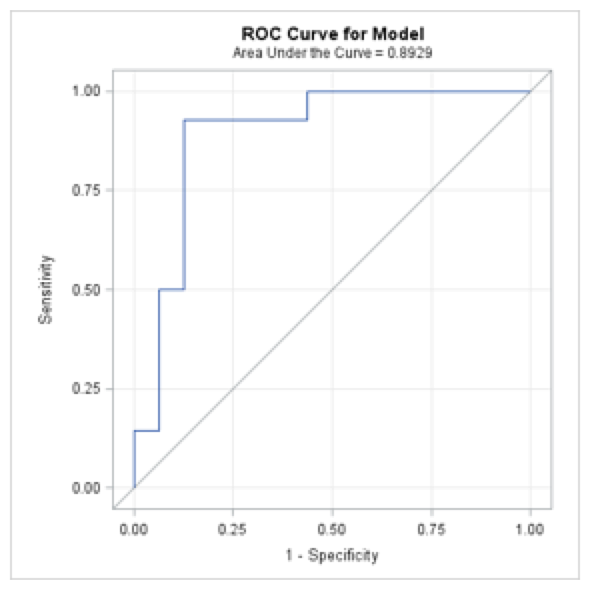


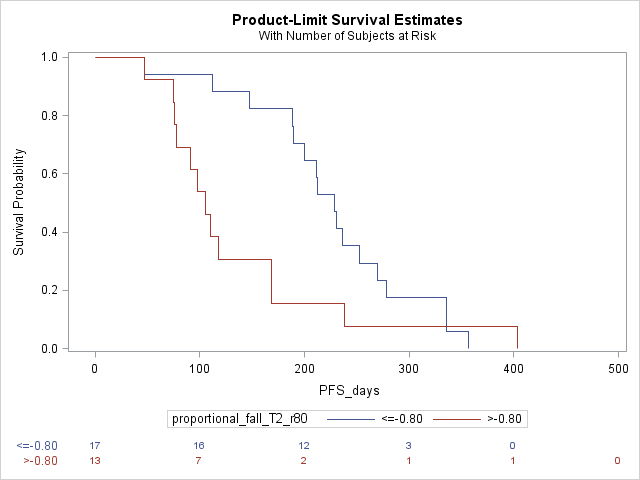


B

A

HR = 0.48 (p=0.0596)

CI=0.223-0.1.030

Median 76 v 188 days


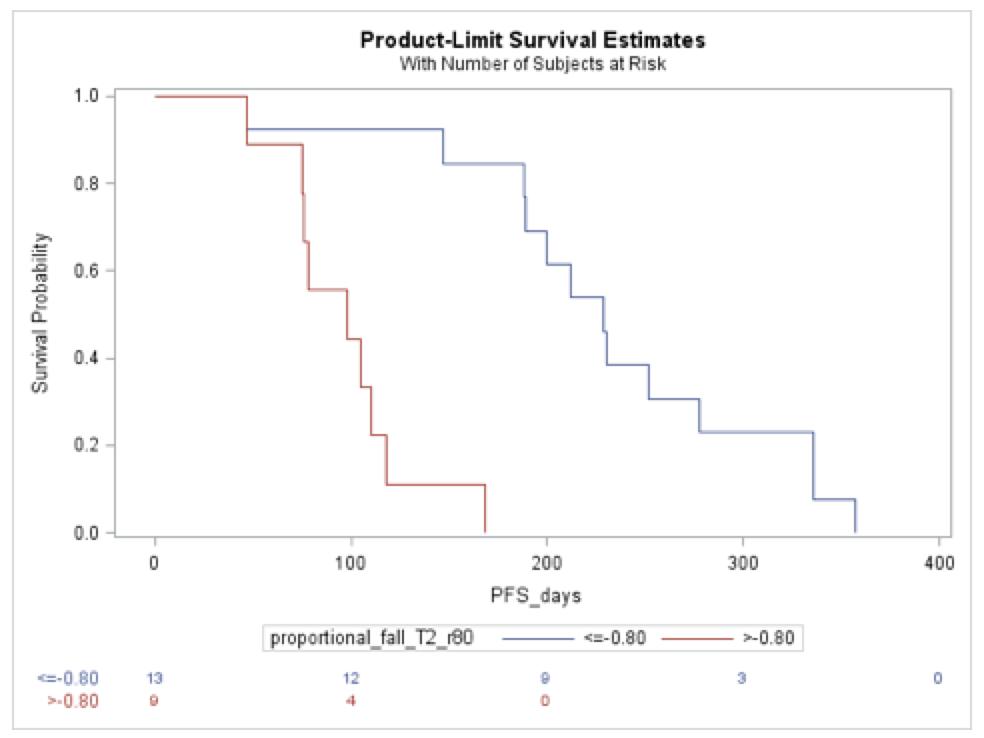

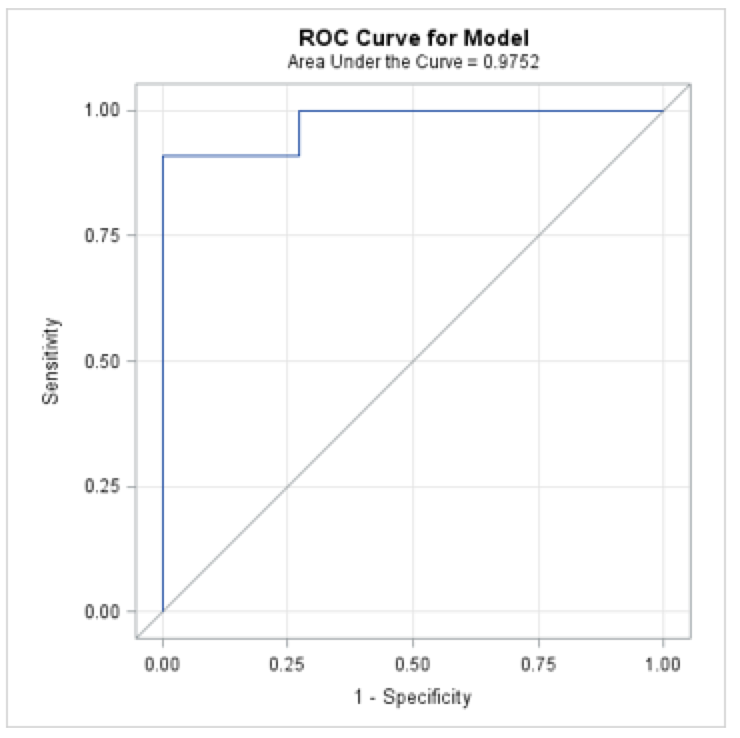


D.

C.

HR = 0.06 (p=0.0006)

CI=0.012-0.302

Median 98 v 229 days

**S5 Fig. ROC curves for TP53MAF decrease after two cycles of chemotherapy to predict 6-mo TTP and Kaplan-Meier curves, including and excluding patients with ascitic drains.** **A.** ROC plot identifies 80% decrease in TP53MAF as the most accurate threshold for predicting 6 months TTP in all patients. **B.** Kaplan-Meier curve showing TTP for patients with decrease less than, or equal to/greater than 80%, after 2 cycles of chemotherapy. **C.** ROC plot identifies 80% decrease in TP53MAF as most accurate threshold for predicting 6 months TTP in patients without ascitic drains. **D.** Kaplan-Meier curve for TP53MAF decrease after 2 cycles of chemotherapy to predict 6 month TTP in patients without ascitic drains.
