# Supplementary material for: Exploratory Analysis of TP53 Mutations in Circulating Tumour DNA as Biomarkers of Treatment Response for Patients with Relapsed High-Grade Serous Ovarian Carcinoma: A Retrospective Study
Source: PLoS Med. 2016 Dec 20;13(12):e1002198. doi: 10.1371/journal.pmed.1002198 (PMC5172526; doi:10.1371/journal.pmed.1002198)

**S6 Fig**. Consistency of predictive category by TP53MAF decrease after one and two cycles in all patients and excluding patients with recent ascitic drains. **A** All patients. **B.** Patients excluding those with recent ascitic drains.


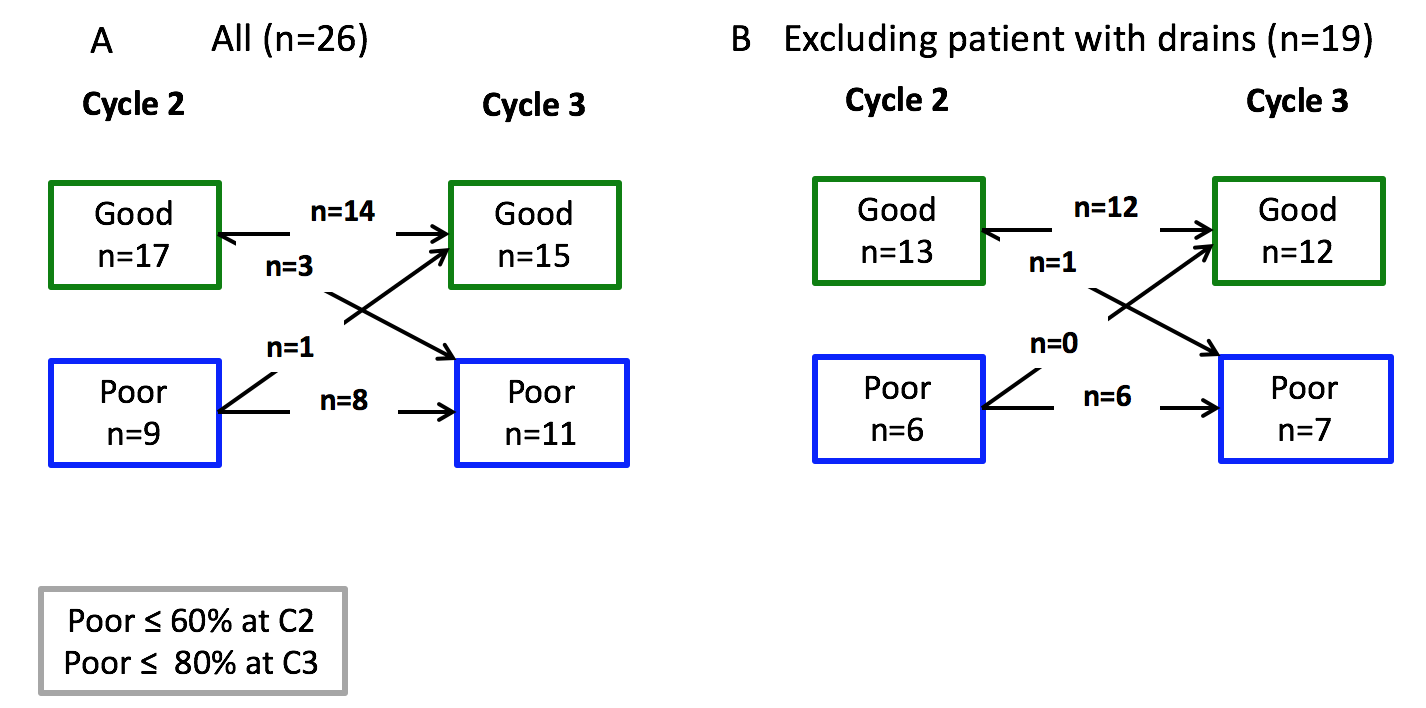

Supplement: S6 Fig — (DOCX) [file pmed.1002198.s010.docx]
