# Supplementary material for: Exploratory Analysis of TP53 Mutations in Circulating Tumour DNA as Biomarkers of Treatment Response for Patients with Relapsed High-Grade Serous Ovarian Carcinoma: A Retrospective Study
Source: PLoS Med. 2016 Dec 20;13(12):e1002198. doi: 10.1371/journal.pmed.1002198 (PMC5172526; doi:10.1371/journal.pmed.1002198)
Supplement: S3 Table — (A) Samples from chemotherapy at relapse. (B) Samples collected during first-line treatment. (PDF) [file pmed.1002198.s013.pdf]

S3 Table. Context of ctDNA samples collected on study relative to chemotherapy cycle.
A. Samples from chemotherapy at relapse. B. Samples collected during 1st line treatment

A.

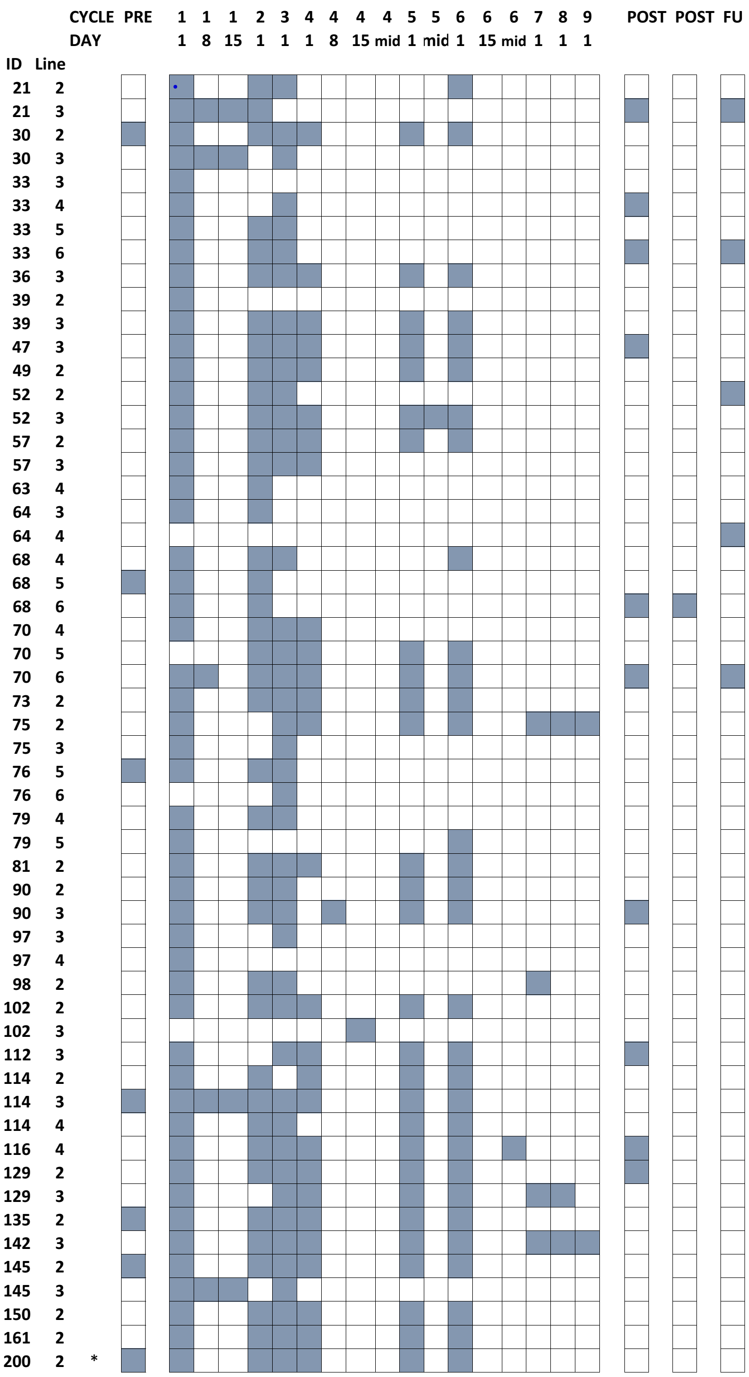

* 1 follow up sample post primary chemotherapy not marked

B.

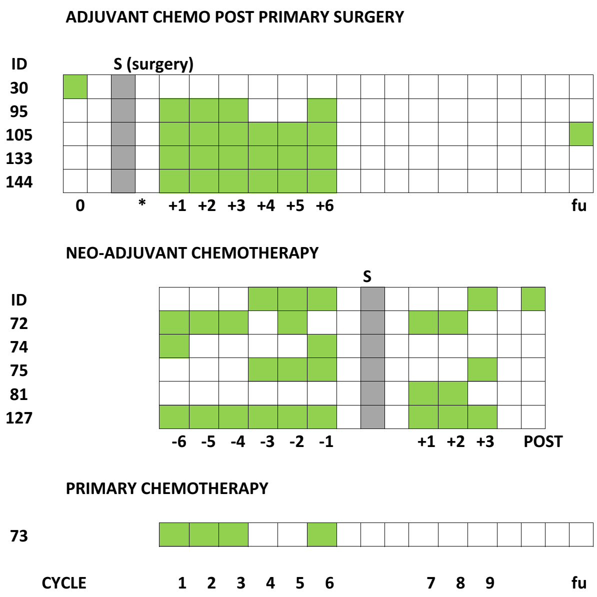

+/- indicates cycle relation to surgery
