# Supplementary material for: Exploratory Analysis of TP53 Mutations in Circulating Tumour DNA as Biomarkers of Treatment Response for Patients with Relapsed High-Grade Serous Ovarian Carcinoma: A Retrospective Study
Source: PLoS Med. 2016 Dec 20;13(12):e1002198. doi: 10.1371/journal.pmed.1002198 (PMC5172526; doi:10.1371/journal.pmed.1002198)
Supplement: S4 Table — (DOCX) [file pmed.1002198.s014.docx]

**S4 Table. Pre-treatment descriptive statistics for T53PMAF, TP53MAC,TP53TAC and CA-125**.

|  | **Mean** | **Std Dev** | **Median** | **Minimum** | **Maximum** | **25th Pctl** | **75th Pctl** |
| --- | --- | --- | --- | --- | --- | --- | --- |
| **Relapsed (n_courses_=51)** |  |  |  |  |  |  |  |
| \| **TP53MAF (%)** \| \| --- \| \| **TP53MAC (AC/ml)** \| \| **TP53TAC (AC/ml)** \| \| **CA-125 (IU/ml)** \| | \| 13.8 \| \| --- \| \| 2066 \| \| 12552 \| \| 933 \| | \| 16.3 \| \| --- \| \| 4763 \| \| 14262 \| \| 1087 \| | \| 8.0 \| \| --- \| \| 639 \| \| 8103 \| \| 422 \| | \| 0 \| \| --- \| \| 0 \| \| 1707 \| \| 46 \| | \| 62.1 \| \| --- \| \| 28577 \| \| 77357 \| \| 4502 \| | \| 1.2 \| \| --- \| \| 53 \| \| 4783 \| \| 203 \| | \| 22.6 \| \| --- \| \| 1762 \| \| 11777 \| \| 1157 \| |
| **New diagnosis (n_courses_=7)** |  |  |  |  |  |  |  |
| \| **TP53MAF (%)** \| \| --- \| \| **TP53MAC (AC/ml)** \| \| **TP53TAC (AC/ml)** \| \| **CA-125 (IU/ml)** \| | \| 4.5 \| \| --- \| \| 1031 \| \| 13899 \| \| 2137 \| | \| 9.3 \| \| --- \| \| 2505 \| \| 8732 \| \| 2504 \| | \| 0.7 \| \| --- \| \| 94 \| \| 11535 \| \| 964 \| | \| 0 \| \| --- \| \| 0 \| \| 6329 \| \| 112 \| | \| 25.4 \| \| --- \| \| 6708 \| \| 26418 \| \| 7090 \| | \| 0.2 \| \| --- \| \| 26 \| \| 6708 \| \| 186 \| | \| 3.4 \| \| --- \| \| 227 \| \| 25507 \| \| 3543 \| |
| **Post primary surgery (n_courses_=4)** |  |  |  |  |  |  |  |
| \| **TP53MAF (%)** \| \| --- \| \| **TP53MAC (AC/ml)** \| \| **TP53TAC/ml (AC/ml)** \| \| **CA-125 (IU/ml)** \| | \| 0.2 \| \| --- \| \| 17 \| \| 7331 \| \| 45 \| | \| 0.1 \| \| --- \| \| 17 \| \| 3628 \| \| 25 \| | \| 0.3 \| \| --- \| \| 15 \| \| 6609 \| \| 50 \| | \| 0 \| \| --- \| \| 0 \| \| 3933 \| \| 16 \| | \| 0.3 \| \| --- \| \| 40 \| \| 12174 \| \| 66 \| | \| 0.1 \| \| --- \| \| 5 \| \| 4615 \| \| 25 \| | \| 0.3 \| \| --- \| \| 30 \| \| 10048 \| \| 66 \| |
