# Supplementary material for: Exploratory Analysis of TP53 Mutations in Circulating Tumour DNA as Biomarkers of Treatment Response for Patients with Relapsed High-Grade Serous Ovarian Carcinoma: A Retrospective Study
Source: PLoS Med. 2016 Dec 20;13(12):e1002198. doi: 10.1371/journal.pmed.1002198 (PMC5172526; doi:10.1371/journal.pmed.1002198)
Supplement: S5 Table — (DOCX) [file pmed.1002198.s015.docx]

**S5 Table. Pearson correlation of pre-treatment TP53MAF, TP53MAC, TP53TAC and CA-125 in relapsed courses.**

| n_courses_=51 | **TP53MAF** | **TP53MAC/ml** | **TP53TAC/ml** | **CA-125 IU/ml** |
| --- | --- | --- | --- | --- |
| **TP53MAF** | 1.00 | 0.98  (p=<0.001) | 0.17  (p=0.236) | 0.49  (p<0.001) |
| **TP53MAC/ml** |  | 1.00 | 0.36  (p=0.010) | 0.50  (p<0.001) |
| **TP53TAC/ml** |  |  | 1.00 | 0.19  (p=0.194) |
| **CA-125 IU/ml** |  |  |  | 1.00 |
