# Supplementary material for: Exploratory Analysis of TP53 Mutations in Circulating Tumour DNA as Biomarkers of Treatment Response for Patients with Relapsed High-Grade Serous Ovarian Carcinoma: A Retrospective Study
Source: PLoS Med. 2016 Dec 20;13(12):e1002198. doi: 10.1371/journal.pmed.1002198 (PMC5172526; doi:10.1371/journal.pmed.1002198)
Supplement: S6 Table — (DOCX) [file pmed.1002198.s016.docx]

**S6 Table. Pearson correlation of blood parameters with volume of disease in relapsed. Rho value is shown, p-value in brackets**

|  | **TP53MAF** | **TP53MAC (AC/ml)** | **TP53TAC**  **(AC/ml)** | **CA-125**  **(IU/ml)** |
| --- | --- | --- | --- | --- |
| All (n=35) | 0.59 (<0.001) | 0.58 (<0.001) | 0.18 (0.294) | 0.52 (0.001) |
| -No ascites (n=22) | 0.82 (<0.001) | 0.81 (<0.001) | 0.29 (0.183) | 0.51 (0.016) |
| -With ascites (n=13) | 0.15 (0.625) | 0.11 (0.713) | 0.113 (0.713) | 0.54 (0.055) |
