# Supplementary material for: Exploratory Analysis of TP53 Mutations in Circulating Tumour DNA as Biomarkers of Treatment Response for Patients with Relapsed High-Grade Serous Ovarian Carcinoma: A Retrospective Study
Source: PLoS Med. 2016 Dec 20;13(12):e1002198. doi: 10.1371/journal.pmed.1002198 (PMC5172526; doi:10.1371/journal.pmed.1002198)
Supplement: S7 Table — (DOCX) [file pmed.1002198.s017.docx]

**S7 Table. Inclusion criteria for courses of treatment eligible for nadir analysis.**

| - ctDNA detectable at baseline (≥20 amplifications/ml) |
| --- |
| - Consecutive samples with at least 1 sample per cycle available to a nadir point or ctDNA falls to 0 |
| - For patients who completed a line of treatment (either as the course is complete or due to cancer progression) in whom ctDNA is still falling, the last (lowest) reading is taken as the nadir |
| - ctDNA is evaluable up to and including 1 cycle length from day 1 of their final cycle |
| - Patients with ascitic drains that could potentially interfere with nadir analysis were excluded or who had recent radiotherapy that could interfere. |
