# Supplementary material for: Exploratory Analysis of TP53 Mutations in Circulating Tumour DNA as Biomarkers of Treatment Response for Patients with Relapsed High-Grade Serous Ovarian Carcinoma: A Retrospective Study
Source: PLoS Med. 2016 Dec 20;13(12):e1002198. doi: 10.1371/journal.pmed.1002198 (PMC5172526; doi:10.1371/journal.pmed.1002198)
Supplement: S8 Table — (DOCX) [file pmed.1002198.s018.docx]

**S8 Table. Sensitivity and specificity by T5P53MAF decrease after one cycle including and excluding courses with recent ascites drains.**

A. Sensitivity and specificity according to TP53MAF decrease after 1 cycle of chemotherapy for predicting TTP <6 months versus ≥ 6 months in relapsed patients including patients with recent ascitic drains (n=31).

| Obs | PROB | POS | NEG | FALPOS | FALNEG | SENSIT | 1MSPEC | cutpoint | j |
| --- | --- | --- | --- | --- | --- | --- | --- | --- | --- |
| 1 | 0.98530 | 1 | 17 | 0 | 13 | 0.07143 | 0.00000 | 0.40823 | 0.07143 |
| 2 | 0.97343 | 2 | 17 | 0 | 12 | 0.14286 | 0.00000 | 0.26334 | 0.14286 |
| 3 | 0.96445 | 3 | 17 | 0 | 11 | 0.21429 | 0.00000 | 0.19116 | 0.21429 |
| 4 | 0.91684 | 3 | 16 | 1 | 11 | 0.21429 | 0.05882 | -0.02497 | 0.15546 |
| 5 | 0.89909 | 4 | 16 | 1 | 10 | 0.28571 | 0.05882 | -0.07610 | 0.22689 |
| 6 | 0.89562 | 5 | 16 | 1 | 9 | 0.35714 | 0.05882 | -0.08514 | 0.29832 |
| 7 | 0.61061 | 6 | 16 | 1 | 8 | 0.42857 | 0.05882 | -0.49316 | 0.36975 |
| 8 | 0.58373 | 7 | 16 | 1 | 7 | 0.50000 | 0.05882 | -0.51999 | 0.44118 |
| 9 | 0.54956 | 8 | 16 | 1 | 6 | 0.57143 | 0.05882 | -0.55340 | 0.51261 |
| 10 | 0.52997 | 9 | 16 | 1 | 5 | 0.64286 | 0.05882 | -0.57234 | 0.58403 |
| 11** | 0.51257 | 10 | 16 | 1 | 4 | 0.71429 | 0.05882 | -0.58908 | 0.65546 |
| 12 | 0.50966 | 10 | 15 | 2 | 4 | 0.71429 | 0.11765 | -0.59187 | 0.59664 |
| 13 | 0.46212 | 10 | 14 | 3 | 4 | 0.71429 | 0.17647 | -0.63759 | 0.53782 |
| 14 | 0.42930 | 10 | 13 | 4 | 4 | 0.71429 | 0.23529 | -0.66949 | 0.47899 |
| 15 | 0.42765 | 11 | 13 | 4 | 3 | 0.78571 | 0.23529 | -0.67111 | 0.55042 |
| 16 | 0.41055 | 11 | 12 | 5 | 3 | 0.78571 | 0.29412 | -0.68797 | 0.49160 |
| 17 | 0.30269 | 11 | 11 | 6 | 3 | 0.78571 | 0.35294 | -0.80148 | 0.43277 |
| 18 | 0.29673 | 11 | 10 | 7 | 3 | 0.78571 | 0.41176 | -0.80830 | 0.37395 |
| 19 | 0.29445 | 11 | 9 | 8 | 3 | 0.78571 | 0.47059 | -0.81092 | 0.31513 |
| 20 | 0.26944 | 12 | 9 | 8 | 2 | 0.85714 | 0.47059 | -0.84060 | 0.38655 |
| 21 | 0.26763 | 13 | 9 | 8 | 1 | 0.92857 | 0.47059 | -0.84280 | 0.45798 |
| 22 | 0.24426 | 13 | 8 | 9 | 1 | 0.92857 | 0.52941 | -0.87229 | 0.39916 |
| 23 | 0.23809 | 14 | 8 | 9 | 0 | 1.00000 | 0.52941 | -0.88037 | 0.47059 |
| 24 | 0.23411 | 14 | 7 | 10 | 0 | 1.00000 | 0.58824 | -0.88568 | 0.41176 |
| 25 | 0.22515 | 14 | 6 | 11 | 0 | 1.00000 | 0.64706 | -0.89783 | 0.35294 |
| 26 | 0.16919 | 14 | 5 | 12 | 0 | 1.00000 | 0.70588 | -0.98316 | 0.29412 |
| 27 | 0.15955 | 14 | 0 | 17 | 0 | 1.00000 | 1.00000 | -1.00002 | 0.00000 |

Note: 1/32 patients was censored at <6 months and was therefore included in the analysis.

**shows observation with optimal cutpoint for sensitivity and specificity.

B. Optimal sensitivity and specificity according to TP53MAF fall after 1 cycle of chemotherapy for predicting TTP <6 months versus ≥ 6 months in relapsed patients excluding patients with recent ascitic drains (n=24).

| Obs | PROB | POS | NEG | FALPOS | FALNEG | SENSIT | 1MSPEC | cutpoint | j |
| --- | --- | --- | --- | --- | --- | --- | --- | --- | --- |
| 1 | 0.99998 | 1 | 12 | 0 | 11 | 0.08333 | 0.00000 | 0.40823 | 0.08333 |
| 2 | 0.99993 | 2 | 12 | 0 | 10 | 0.16667 | 0.00000 | 0.26334 | 0.16667 |
| 3 | 0.99798 | 3 | 12 | 0 | 9 | 0.25000 | 0.00000 | -0.07610 | 0.25000 |
| 4 | 0.99779 | 4 | 12 | 0 | 8 | 0.33333 | 0.00000 | -0.08514 | 0.33333 |
| 5 | 0.88874 | 5 | 12 | 0 | 7 | 0.41667 | 0.00000 | -0.49315 | 0.41667 |
| 6 | 0.85968 | 6 | 12 | 0 | 6 | 0.50000 | 0.00000 | -0.51998 | 0.50000 |
| 7 | 0.81489 | 7 | 12 | 0 | 5 | 0.58333 | 0.00000 | -0.55340 | 0.58333 |
| 8 | 0.78498 | 8 | 12 | 0 | 4 | 0.66667 | 0.00000 | -0.57233 | 0.66667 |
| 9** | 0.75573 | 9 | 12 | 0 | 3 | 0.75000 | 0.00000 | -0.58907 | 0.75000 |
| 10 | 0.65694 | 9 | 11 | 1 | 3 | 0.75000 | 0.08333 | -0.63758 | 0.66667 |
| 11 | 0.58277 | 9 | 10 | 2 | 3 | 0.75000 | 0.16667 | -0.66948 | 0.58333 |
| 12 | 0.57887 | 10 | 10 | 2 | 2 | 0.83333 | 0.16667 | -0.67110 | 0.66667 |
| 13 | 0.53778 | 10 | 9 | 3 | 2 | 0.83333 | 0.25000 | -0.68796 | 0.58333 |
| 14 | 0.27467 | 10 | 8 | 4 | 2 | 0.83333 | 0.33333 | -0.80147 | 0.50000 |
| 15 | 0.26145 | 10 | 7 | 5 | 2 | 0.83333 | 0.41667 | -0.80829 | 0.41667 |
| 16 | 0.25646 | 10 | 6 | 6 | 2 | 0.83333 | 0.50000 | -0.81091 | 0.33333 |
| 17 | 0.20106 | 11 | 6 | 6 | 1 | 0.91667 | 0.50000 | -0.84279 | 0.41667 |
| 18 | 0.14790 | 12 | 6 | 6 | 0 | 1.00000 | 0.50000 | -0.88036 | 0.50000 |
| 19 | 0.14140 | 12 | 5 | 7 | 0 | 1.00000 | 0.58333 | -0.88567 | 0.41667 |
| 20 | 0.05910 | 12 | 4 | 8 | 0 | 1.00000 | 0.66667 | -0.98315 | 0.33333 |
| 21 | 0.05048 | 12 | 0 | 12 | 0 | 1.00000 | 1.00000 | -1.00001 | 0.00000 |

Note: 1/25 patients was censored at <6 months and was therefore included in the analysis.

**shows observation with optimal cutpoint for sensitivity and specificity.
