# Supplementary material for: Exploratory Analysis of TP53 Mutations in Circulating Tumour DNA as Biomarkers of Treatment Response for Patients with Relapsed High-Grade Serous Ovarian Carcinoma: A Retrospective Study
Source: PLoS Med. 2016 Dec 20;13(12):e1002198. doi: 10.1371/journal.pmed.1002198 (PMC5172526; doi:10.1371/journal.pmed.1002198)
Supplement: S9 Table — (DOCX) [file pmed.1002198.s019.docx]

**S9 Table.** Univariable and multivariable analysis of decrease in TP53MAF as a continuous variable to predict TTP after 1 cycle of chemotherapy.

|  |  | Univariable |  |  | Multivariable |  |
| --- | --- | --- | --- | --- | --- | --- |
| n_courses_=32; variable (units) | HR | CI | P value | HR | CI | P value |
| TP53MAF decrease from C1 to C2 (%) | **0.99** | **0.98-0.99** | **0.001** | **0.99** | **0.98-0.999** | **0.034** |
| CA-125 decrease from C1 to C2 (%) | 0.997 | 0.99-1.01 | 0.456 | 1.002 | 0.99-1.01 | 0.751 |
| Age (years) | 0.99 | 0.95-1.04 | 0.840 | 0.98 | 0.93-1.04 | 0.500 |
| Performance status (0-2) | 0.78 | 0.35-1.76 | 0.549 | 0.80 | 0.32-1.99 | 0.627 |
| Platinum sensitive (y/n) | 0.49 | 0.23-1.02 | 0.057 | 0.73 | 0.28-1.92 | 0.522 |
| No lines chemotherapy (2,≥3) | 0.53 | 0.24-1.16 | 0.114 | 0.74 | 0.26-2.10 | 0.570 |
| Volume of disease (10 cm^3^) | **1.02** | **1.001-1.028** | **0.031** | 1.002 | 0.99-1.02 | 0.819 |
| Ascites (n/y) | 1.36 | 0.66-2.81 | 0.409 | 1.81 | 0.78-4.19 | 0.168 |
