# Supplementary material for: Exploratory Analysis of TP53 Mutations in Circulating Tumour DNA as Biomarkers of Treatment Response for Patients with Relapsed High-Grade Serous Ovarian Carcinoma: A Retrospective Study
Source: PLoS Med. 2016 Dec 20;13(12):e1002198. doi: 10.1371/journal.pmed.1002198 (PMC5172526; doi:10.1371/journal.pmed.1002198)
Supplement: S10 Table — (DOCX) [file pmed.1002198.s020.docx]

**S10 Table.** Univariable and multivariable analysis of pre-treatment TP53MAC as a continuous variable to predict TTP.

|  |  | Univariable |  |  | Multivariable |  |
| --- | --- | --- | --- | --- | --- | --- |
| n_courses_=49; variable (units) | HR | CI | P value | HR | CI | P value |
| TP53MAC (10^3^ copies/ml) | **1.08** | **1.03-1.14** | **0.003** | 1.04 | 0.94-1.14 | 0.441 |
| CA-125 (10^2^ IU/ml) | 1.02 | 0.998-1.05 | 0.078 | 1.01 | 0.988-1.04 | 0.292 |
| TP53TAC (10^3^ copies/ml) | **1.03** | **1.006-1.04** | **0.009** | 1.02 | 0.990-1.04 | 0.224 |
| Age (years) | **0.96** | **0.93-1.00** | **0.030** | **0.96** | **0.92-0.998** | **0.041** |
| PS (0-2) | 0.72 | 0.44-1.18 | 0.192 | 0.71 | 0.40-1.26 | 0.242 |
| Platinum sensitive (y/n) | **0.35** | **0.18-0.68** | **0.002** | 0.46 | 0.20-1.02 | 0.054 |
| No. of lines chemotherapy (2,≥3) | **0.43** | **0.22-0.83** | **0.013** | 0.76 | 0.33-1.76 | 0.528 |
| Volume of disease (10 cm^3^) | **1.02** | **1.007-1.03** | **0.002** | 1.01 | 0.98-1.03 | 0.684 |
| Ascites (n/y) | 0.95 | 0.53-1.71 | 0.858 | 0.92 | 0.48-1.77 | 0.803 |
