# Supplementary material for: Exploratory Analysis of TP53 Mutations in Circulating Tumour DNA as Biomarkers of Treatment Response for Patients with Relapsed High-Grade Serous Ovarian Carcinoma: A Retrospective Study
Source: PLoS Med. 2016 Dec 20;13(12):e1002198. doi: 10.1371/journal.pmed.1002198 (PMC5172526; doi:10.1371/journal.pmed.1002198)
Supplement: S11 Table — (DOCX) [file pmed.1002198.s021.docx]

**S11 Table.** Univariable and multivariable analysis of decrease in TP53MAC as continuous variable to predict TTP after 1 cycle of chemotherapy.

|  |  | Univariable |  |  | Multivariable |  |
| --- | --- | --- | --- | --- | --- | --- |
| n_courses_=32; variable (units) | HR | CI | P value | HR | CI | P value |
| TP53MAC decrease from C1 to C2 (%) | **0.995** | **0.992-0.998** | **0.004** | 0.996 | 0.989-1.002 | 0.207 |
| CA-125 decrease from C1 to C2 (%) | 0.997 | 0.99-1.01 | 0.456 | 1.002 | 0.99-1.01 | 0.654 |
| Age (years) | 0.99 | 0.95-1.04 | 0.840 | 0.99 | 0.93-1.04 | 0.653 |
| Performance status (0-2) | 0.78 | 0.35-1.76 | 0.549 | 0.76 | 0.30-1.92 | 0.559 |
| Platinum sensitive (y/n) | 0.49 | 0.23-1.02 | 0.057 | 0.50 | 0.20-1.21 | 0.124 |
| No lines chemotherapy (2,≥3) | 0.53 | 0.24-1.16 | 0.114 | 0.63 | 0.24-1.68 | 0.360 |
| Volume of disease (10 cm^3^) | **1.02** | **1.001-1.028** | **0.031** | 0.997 | 0.97-1.02 | 0.774 |
| Ascites (n/y) | 1.36 | 0.66-2.81 | 0.409 | 1.39 | 0.62-3.16 | 0.426 |
