# Supplementary material for: Exploratory Analysis of TP53 Mutations in Circulating Tumour DNA as Biomarkers of Treatment Response for Patients with Relapsed High-Grade Serous Ovarian Carcinoma: A Retrospective Study
Source: PLoS Med. 2016 Dec 20;13(12):e1002198. doi: 10.1371/journal.pmed.1002198 (PMC5172526; doi:10.1371/journal.pmed.1002198)
Supplement: S12 Table — (DOCX) [file pmed.1002198.s022.docx]

**S12 Table. Sensitivity and specificity of CA-125 decrease after one cycle including and excluding courses with recent ascites drains.**

1. Sensitivity and specificity by CA-125 decrease after 1 cycle of chemotherapy for predicting TTP <6 months versus ≥ 6 months in relapsed patients including patients with recent ascitic drains (n=31).

| Obs | PROB | POS | NEG | FALPOS | FALNEG | SENSIT | 1MSPEC | cutpoint | j |
| --- | --- | --- | --- | --- | --- | --- | --- | --- | --- |
| 1 | 0.54471 | 0 | 16 | 1 | 14 | 0.00000 | 0.05882 | 1.09237 | -0.05882 |
| 2 | 0.53942 | 1 | 16 | 1 | 13 | 0.07143 | 0.05882 | 1.02496 | 0.01261 |
| 3 | 0.50104 | 1 | 15 | 2 | 13 | 0.07143 | 0.11765 | 0.53881 | -0.04622 |
| 4 | 0.49850 | 2 | 15 | 2 | 12 | 0.14286 | 0.11765 | 0.50669 | 0.02521 |
| 5 | 0.49324 | 2 | 14 | 3 | 12 | 0.14286 | 0.17647 | 0.44008 | -0.03361 |
| 6 | 0.48054 | 3 | 14 | 3 | 11 | 0.21429 | 0.17647 | 0.27948 | 0.03782 |
| 7 | 0.47665 | 3 | 13 | 4 | 11 | 0.21429 | 0.23529 | 0.23019 | -0.02101 |
| 8 | 0.47102 | 3 | 12 | 5 | 11 | 0.21429 | 0.29412 | 0.15883 | -0.07983 |
| 9 | 0.46727 | 3 | 11 | 6 | 11 | 0.21429 | 0.35294 | 0.11117 | -0.13866 |
| 10 | 0.46719 | 4 | 11 | 6 | 10 | 0.28571 | 0.35294 | 0.11016 | -0.06723 |
| 11 | 0.46483 | 5 | 11 | 6 | 9 | 0.35714 | 0.35294 | 0.08019 | 0.00420 |
| 12 | 0.45852 | 6 | 11 | 6 | 8 | 0.42857 | 0.35294 | 0.00005 | 0.07563 |
| 13 | 0.45698 | 6 | 10 | 7 | 8 | 0.42857 | 0.41176 | -0.01956 | 0.01681 |
| 14 | 0.45568 | 6 | 9 | 8 | 8 | 0.42857 | 0.47059 | -0.03614 | -0.04202 |
| 15 | 0.44685 | 7 | 9 | 8 | 7 | 0.50000 | 0.47059 | -0.14894 | 0.02941 |
| 16 | 0.44515 | 8 | 9 | 8 | 6 | 0.57143 | 0.47059 | -0.17064 | 0.10084 |
| 17 | 0.44447 | 9 | 9 | 8 | 5 | 0.64286 | 0.47059 | -0.17935 | 0.17227 |
| 18 | 0.44267 | 9 | 8 | 9 | 5 | 0.64286 | 0.52941 | -0.20234 | 0.11345 |
| 19 | 0.43507 | 10 | 8 | 9 | 4 | 0.71429 | 0.52941 | -0.29988 | 0.18487 |
| 20 | 0.43248 | 10 | 7 | 10 | 4 | 0.71429 | 0.58824 | -0.33331 | 0.12605 |
| 21 | 0.43113 | 10 | 6 | 11 | 4 | 0.71429 | 0.64706 | -0.35062 | 0.06723 |
| 22 | 0.42707 | 10 | 5 | 12 | 4 | 0.71429 | 0.70588 | -0.40307 | 0.00840 |
| 23 | 0.42604 | 11 | 5 | 12 | 3 | 0.78571 | 0.70588 | -0.41627 | 0.07983 |
| 24 | 0.42268 | 12 | 5 | 12 | 2 | 0.85714 | 0.70588 | -0.45985 | 0.15126 |
| 25 | 0.42197 | 13 | 5 | 12 | 1 | 0.92857 | 0.70588 | -0.46906 | 0.22269 |
| 26 | 0.41671 | 13 | 4 | 13 | 1 | 0.92857 | 0.76471 | -0.53730 | 0.16387 |
| 27 | 0.41166 | 14 | 4 | 13 | 0 | 1.00000 | 0.76471 | -0.60300 | 0.23529 |
| 28 | 0.40794 | 14 | 3 | 14 | 0 | 1.00000 | 0.82353 | -0.65168 | 0.17647 |
| 29 | 0.40599 | 14 | 2 | 15 | 0 | 1.00000 | 0.88235 | -0.67722 | 0.11765 |
| 30 | 0.40587 | 14 | 1 | 16 | 0 | 1.00000 | 0.94118 | -0.67883 | 0.05882 |
| 31 | 0.40067 | 14 | 0 | 17 | 0 | 1.00000 | 1.00000 | -0.74706 | 0.00000 |

Note: 1/32 patients was censored at <6 months and was therefore included in the analysis

B. Sensitivity and specificity by CA-125 fall after 1 cycle of chemotherapy for predicting TTP <6 months versus ≥ 6 months in relapsed patients excluding patients with recent ascitic drains (n=24).

| Obs | PROB | POS | NEG | FALPOS | FALNEG | SENSIT | 1MSPEC | cutpoint | j |
| --- | --- | --- | --- | --- | --- | --- | --- | --- | --- |
| 1 | 0.83371 | 1 | 12 | 0 | 11 | 0.08333 | 0.00000 | 1.02478 | 0.08333 |
| 2 | 0.71990 | 1 | 11 | 1 | 11 | 0.08333 | 0.08333 | 0.53867 | 0.00000 |
| 3 | 0.71091 | 2 | 11 | 1 | 10 | 0.16667 | 0.08333 | 0.50656 | 0.08333 |
| 4 | 0.64280 | 3 | 11 | 1 | 9 | 0.25000 | 0.08333 | 0.27937 | 0.16667 |
| 5 | 0.62709 | 3 | 10 | 2 | 9 | 0.25000 | 0.16667 | 0.23008 | 0.08333 |
| 6 | 0.58812 | 3 | 9 | 3 | 9 | 0.25000 | 0.25000 | 0.11107 | 0.00000 |
| 7 | 0.58778 | 4 | 9 | 3 | 8 | 0.33333 | 0.25000 | 0.11007 | 0.08333 |
| 8 | 0.57777 | 5 | 9 | 3 | 7 | 0.41667 | 0.25000 | 0.08010 | 0.16667 |
| 9 | 0.53835 | 5 | 8 | 4 | 7 | 0.41667 | 0.33333 | -0.03622 | 0.08333 |
| 10 | 0.49967 | 6 | 8 | 4 | 6 | 0.50000 | 0.33333 | -0.14901 | 0.16667 |
| 11 | 0.49221 | 7 | 8 | 4 | 5 | 0.58333 | 0.33333 | -0.17071 | 0.25000 |
| 12 | 0.48922 | 8 | 8 | 4 | 4 | 0.66667 | 0.33333 | -0.17941 | 0.33333 |
| 13 | 0.48133 | 8 | 7 | 5 | 4 | 0.66667 | 0.41667 | -0.20241 | 0.25000 |
| 14 | 0.44799 | 9 | 7 | 5 | 3 | 0.75000 | 0.41667 | -0.29994 | 0.33333 |
| 15 | 0.43666 | 9 | 6 | 6 | 3 | 0.75000 | 0.50000 | -0.33336 | 0.25000 |
| 16 | 0.43081 | 9 | 5 | 7 | 3 | 0.75000 | 0.58333 | -0.35067 | 0.16667 |
| 17 | 0.41323 | 9 | 4 | 8 | 3 | 0.75000 | 0.66667 | -0.40311 | 0.08333 |
| 18 | 0.40884 | 10 | 4 | 8 | 2 | 0.83333 | 0.66667 | -0.41632 | 0.16667 |
| 19 | 0.39143 | 11 | 4 | 8 | 1 | 0.91667 | 0.66667 | -0.46910 | 0.25000 |
| 20 | 0.36933 | 11 | 3 | 9 | 1 | 0.91667 | 0.75000 | -0.53733 | 0.16667 |
| 21 | 0.34855 | 12 | 3 | 9 | 0 | 1.00000 | 0.75000 | -0.60303 | 0.25000 |
| 22 | 0.33352 | 12 | 2 | 10 | 0 | 1.00000 | 0.83333 | -0.65170 | 0.16667 |
| 23 | 0.32576 | 12 | 1 | 11 | 0 | 1.00000 | 0.91667 | -0.67724 | 0.08333 |
| 24 | 0.30504 | 12 | 0 | 12 | 0 | 1.00000 | 1.00000 | -0.74707 | 0.00000 |

Note: 1/25 patients was censored at <6 months and was therefore included in the analysis
