# Supplementary material for: Exploratory Analysis of TP53 Mutations in Circulating Tumour DNA as Biomarkers of Treatment Response for Patients with Relapsed High-Grade Serous Ovarian Carcinoma: A Retrospective Study
Source: PLoS Med. 2016 Dec 20;13(12):e1002198. doi: 10.1371/journal.pmed.1002198 (PMC5172526; doi:10.1371/journal.pmed.1002198)
Supplement: S13 Table — (DOCX) [file pmed.1002198.s023.docx]

**S13 Table.** Univariable and multivariable analysis of decrease in TP53MAF as a continuous variable to predict TTP after two cycles of chemotherapy.

|  |  | Univariable |  |  | Multivariable |  |
| --- | --- | --- | --- | --- | --- | --- |
| n_courses_=30; variable (units) | HR | CI | P value | HR | CI | P value |
| TP53MAF decrease from C1 to C3 (%) | 0.997 | 0.993-1.002 | 0.253 | 0.998 | 0.992-1.004 | 0.521 |
| CA-125 decrease from C1 to C3 (%) | **0.991** | **0.983-0.999** | **0.025** | 0.991 | 0.982-1.001 | 0.070 |
| Age (years) | 0.92 | 0.95-1.04 | 0.727 | 0.98 | 0.93-1.03 | 0.374 |
| Performance status (0-2) | 1.21 | 0.56-2.60 | 0.630 | 0.999 | 0.44-2.27 | 0.997 |
| Platinum sensitive (y/n) | **0.43** | **0.20-0.94** | **0.033** | 0.50 | 0.21-1.20 | 0.121 |
| No lines chemotherapy (2,≥3) | 0.69 | 0.31-1.53 | 0.359 | 0.99 | 0.34-2.89 | 0.988 |
| Volume of disease (10 cm^3^) | 1.01 | 0.995-1.028 | 0.168 | 1.004 | 0.99-1.02 | 0.649 |
| Ascites (n/y) | 1.70 | 0.81-3.58 | 0.164 | 1.989 | 0.85-4.64 | 0.112 |
