# Supplementary material for: Exploratory Analysis of TP53 Mutations in Circulating Tumour DNA as Biomarkers of Treatment Response for Patients with Relapsed High-Grade Serous Ovarian Carcinoma: A Retrospective Study
Source: PLoS Med. 2016 Dec 20;13(12):e1002198. doi: 10.1371/journal.pmed.1002198 (PMC5172526; doi:10.1371/journal.pmed.1002198)
Supplement: S14 Table — (DOCX) [file pmed.1002198.s024.docx]

**S14 Table. Sensitivity and specificity of T5P53MAF decrease after two cycles of chemotherapy including and excluding courses with recent ascites drains.**

A. Optimal sensitivity and specificity by TP53MAF decrease after 2 cycles of chemotherapy for predicting TTP <6 months versus ≥ 6 months in relapsed patients including patients with recent ascitic drains (n=30**).**

| Obs | PROB | POS | NEG | FALPOS | FALNEG | SENSIT | 1MSPEC | cutpoint | j |
| --- | --- | --- | --- | --- | --- | --- | --- | --- | --- |
| 1 | 0.99995 | 1 | 16 | 0 | 13 | 0.07143 | 0.0000 | 3.27359 | 0.07143 |
| 2 | 0.96740 | 2 | 16 | 0 | 12 | 0.14286 | 0.0000 | 0.70593 | 0.14286 |
| 3 | 0.95921 | 2 | 15 | 1 | 12 | 0.14286 | 0.0625 | 0.61403 | 0.08036 |
| 4 | 0.92416 | 3 | 15 | 1 | 11 | 0.21429 | 0.0625 | 0.35420 | 0.15179 |
| 5 | 0.73296 | 4 | 15 | 1 | 10 | 0.28571 | 0.0625 | -0.23494 | 0.22321 |
| 6 | 0.61742 | 5 | 15 | 1 | 9 | 0.35714 | 0.0625 | -0.44483 | 0.29464 |
| 7 | 0.60711 | 6 | 15 | 1 | 8 | 0.42857 | 0.0625 | -0.46200 | 0.36607 |
| 8 | 0.59563 | 7 | 15 | 1 | 7 | 0.50000 | 0.0625 | -0.48094 | 0.43750 |
| 9 | 0.49297 | 7 | 14 | 2 | 7 | 0.50000 | 0.1250 | -0.64512 | 0.37500 |
| 10 | 0.49237 | 8 | 14 | 2 | 6 | 0.57143 | 0.1250 | -0.64607 | 0.44643 |
| 11 | 0.48447 | 9 | 14 | 2 | 5 | 0.64286 | 0.1250 | -0.65857 | 0.51786 |
| 12 | 0.43771 | 10 | 14 | 2 | 4 | 0.71429 | 0.1250 | -0.73300 | 0.58929 |
| 13 | 0.42971 | 11 | 14 | 2 | 3 | 0.78571 | 0.1250 | -0.74587 | 0.66071 |
| 14 | 0.39433 | 12 | 14 | 2 | 2 | 0.85714 | 0.1250 | -0.80362 | 0.73214 |
| 15** | 0.36369 | 13 | 14 | 2 | 1 | 0.92857 | 0.1250 | -0.85509 | 0.80357 |
| 16 | 0.35877 | 13 | 13 | 3 | 1 | 0.92857 | 0.1875 | -0.86352 | 0.74107 |
| 17 | 0.34763 | 13 | 12 | 4 | 1 | 0.92857 | 0.2500 | -0.88280 | 0.67857 |
| 18 | 0.33135 | 13 | 11 | 5 | 1 | 0.92857 | 0.3125 | -0.91151 | 0.61607 |
| 19 | 0.31039 | 13 | 10 | 6 | 1 | 0.92857 | 0.3750 | -0.94953 | 0.55357 |
| 20 | 0.30624 | 13 | 9 | 7 | 1 | 0.92857 | 0.4375 | -0.95721 | 0.49107 |
| 21 | 0.29294 | 14 | 9 | 7 | 0 | 1.00000 | 0.4375 | -0.98227 | 0.56250 |
| 22 | 0.28373 | 14 | 0 | 16 | 0 | 1.00000 | 1.0000 | -1.00002 | 0.00000 |

**shows observation with optimal cutpoint for sensitivity and specificity.

B. Optimal sensitivity and specificity for TP53MAF fall after 2 cycles of chemotherapy for predicting TTP <6 months versus ≥ 6 months in relapsed patients excluding patients with recent ascitic drains (n=22).

| Obs | PROB | POS | NEG | FALPOS | FALNEG | SENSIT | 1MSPEC | cutpoint | j |
| --- | --- | --- | --- | --- | --- | --- | --- | --- | --- |
| 1 | 1.00000 | 2 | 11 | 0 | 9 | 0.18182 | 0.00000 | 0.35422 | 0.18182 |
| 2 | 0.99999 | 3 | 11 | 0 | 8 | 0.27273 | 0.00000 | -0.23492 | 0.27273 |
| 3 | 0.99968 | 4 | 11 | 0 | 7 | 0.36364 | 0.00000 | -0.44482 | 0.36364 |
| 4 | 0.99955 | 5 | 11 | 0 | 6 | 0.45455 | 0.00000 | -0.46198 | 0.45455 |
| 5 | 0.99934 | 6 | 11 | 0 | 5 | 0.54545 | 0.00000 | -0.48092 | 0.54545 |
| 6 | 0.98313 | 7 | 11 | 0 | 4 | 0.63636 | 0.00000 | -0.64605 | 0.63636 |
| 7 | 0.97851 | 8 | 11 | 0 | 3 | 0.72727 | 0.00000 | -0.65855 | 0.72727 |
| 8 | 0.91290 | 9 | 11 | 0 | 2 | 0.81818 | 0.00000 | -0.73298 | 0.81818 |
| 9** | 0.72232 | 10 | 11 | 0 | 1 | 0.90909 | 0.00000 | -0.80360 | 0.90909 |
| 10 | 0.44369 | 10 | 10 | 1 | 1 | 0.90909 | 0.09091 | -0.86350 | 0.81818 |
| 11 | 0.35284 | 10 | 9 | 2 | 1 | 0.90909 | 0.18182 | -0.88278 | 0.72727 |
| 12 | 0.12747 | 10 | 8 | 3 | 1 | 0.90909 | 0.27273 | -0.94951 | 0.63636 |
| 13 | 0.07112 | 11 | 8 | 3 | 0 | 1.00000 | 0.27273 | -0.98225 | 0.72727 |
| 14 | 0.05118 | 11 | 0 | 11 | 0 | 1.00000 | 1.00000 | -1.00000 | 0.00000 |

**shows observation with optimal cutpoint for sensitivity and specificity.
