# Supplementary material for: Exploratory Analysis of TP53 Mutations in Circulating Tumour DNA as Biomarkers of Treatment Response for Patients with Relapsed High-Grade Serous Ovarian Carcinoma: A Retrospective Study
Source: PLoS Med. 2016 Dec 20;13(12):e1002198. doi: 10.1371/journal.pmed.1002198 (PMC5172526; doi:10.1371/journal.pmed.1002198)
Supplement: S15 Table — (DOCX) [file pmed.1002198.s025.docx]

**S15 Table.** Univariable and multivariable analysis of TP53MAF decrease of >80% after 2 cycles of chemotherapy as a predictor of TTP (n=30).

|  |  | | Univariable |  |  | Multivariable |  |
| --- | --- | --- | --- | --- | --- | --- | --- |
| n_courses_=30; variable (units) | HR | | CI | P value | HR | CI | P value |
| TP53MAF decrease >80% from C1 to C3 | | 0.48 | 0.22-1.03 | 0.060 | **0.26** | **0.08-0.93** | **0.037** |
| CA-125 decrease >50% from C1 to C3 | | 0.66 | 0.32-1.38 | 0.270 | 0.67 | 0.28-1.57 | 0.354 |
| Age (years) | | 0.99 | 0.95-1.04 | 0.727 | 0.97 | 0.91-1.03 | 0.259 |
| Performance status (0-2) | | 1.21 | 0.56-2.60 | 0.630 | 0.76 | 0.32-1.78 | 0.523 |
| Platinum sensitive (y/n) | | **0.43** | **0.20-0.94** | **0.033** | **0.32** | **0.12-0.86** | **0.023** |
| No lines chemotherapy (2,≥3) | | 0.69 | 0.31-1.53 | 0.359 | 1.47 | 0.494-4.37 | 0.490 |
| Volume of disease (10 cm^3^) | | 1.01 | 0.995-1.03 | 0.168 | 0.995 | 0.97-1.02 | 0.660 |
| Ascites (n/y) | | 1.70 | 0.81-3.58 | 0.164 | 2.00 | 0.85-4.69 | 0.110 |
