# Supplementary material for: Exploratory Analysis of TP53 Mutations in Circulating Tumour DNA as Biomarkers of Treatment Response for Patients with Relapsed High-Grade Serous Ovarian Carcinoma: A Retrospective Study
Source: PLoS Med. 2016 Dec 20;13(12):e1002198. doi: 10.1371/journal.pmed.1002198 (PMC5172526; doi:10.1371/journal.pmed.1002198)
Supplement: S16 Table — (DOCX) [file pmed.1002198.s026.docx]

**S16 Table. Sensitivity and specificity of CA-125 decrease after two cycles of chemotherapy including and excluding courses with recent ascitic drainage.**

1. Sensitivity and specificity of CA-125 decrease after 2 cycles of chemotherapy for predicting TTP <6 months versus ≥ 6 months in relapsed patients including patients with recent ascitic drains (n=30).

| **Obs** | **_PROB_** | **_POS_** | **_NEG_** | **_FALPOS_** | **_FALNEG_** | **_SENSIT_** | **_1MSPEC_** | **cutpoint** | **j** |
| --- | --- | --- | --- | --- | --- | --- | --- | --- | --- |
| **1** | 0.94687 | 1 | 16 | 0 | 13 | 0.07143 | 0.0000 | 1.31472 | 0.07143 |
| **2** | 0.86828 | 2 | 16 | 0 | 12 | 0.14286 | 0.0000 | 0.76088 | 0.14286 |
| **3** | 0.83598 | 2 | 15 | 1 | 12 | 0.14286 | 0.0625 | 0.61766 | 0.08036 |
| **4** | 0.75978 | 3 | 15 | 1 | 11 | 0.21429 | 0.0625 | 0.35193 | 0.15179 |
| **5** | 0.73843 | 4 | 15 | 1 | 10 | 0.28571 | 0.0625 | 0.28865 | 0.22321 |
| **6** | 0.61086 | 5 | 15 | 1 | 9 | 0.35714 | 0.0625 | -0.03818 | 0.29464 |
| **7** | 0.57952 | 6 | 15 | 1 | 8 | 0.42857 | 0.0625 | -0.11065 | 0.36607 |
| **8** | 0.56342 | 6 | 14 | 2 | 8 | 0.42857 | 0.1250 | -0.14726 | 0.30357 |
| **9** | 0.53270 | 6 | 13 | 3 | 8 | 0.42857 | 0.1875 | -0.21636 | 0.24107 |
| **10** | 0.52242 | 6 | 12 | 4 | 8 | 0.42857 | 0.2500 | -0.23933 | 0.17857 |
| **11** | 0.51579 | 7 | 12 | 4 | 7 | 0.50000 | 0.2500 | -0.25413 | 0.25000 |
| **12** | 0.49325 | 7 | 11 | 5 | 7 | 0.50000 | 0.3125 | -0.30435 | 0.18750 |
| **13** | 0.47890 | 8 | 11 | 5 | 6 | 0.57143 | 0.3125 | -0.33634 | 0.25893 |
| **14** | 0.46275 | 9 | 11 | 5 | 5 | 0.64286 | 0.3125 | -0.37244 | 0.33036 |
| **15** | 0.44835 | 9 | 10 | 6 | 5 | 0.64286 | 0.3750 | -0.40476 | 0.26786 |
| **16** | 0.40779 | 10 | 10 | 6 | 4 | 0.71429 | 0.3750 | -0.49708 | 0.33929 |
| **17** | 0.38508 | 11 | 10 | 6 | 3 | 0.78571 | 0.3750 | -0.54995 | 0.41071 |
| **18** | 0.36893 | 11 | 9 | 7 | 3 | 0.78571 | 0.4375 | -0.58824 | 0.34821 |
| **19** | 0.34325 | 11 | 8 | 8 | 3 | 0.78571 | 0.5000 | -0.65063 | 0.28571 |
| **20** | 0.32297 | 11 | 7 | 9 | 3 | 0.78571 | 0.5625 | -0.70149 | 0.22321 |
| **21** | 0.32070 | 12 | 7 | 9 | 2 | 0.85714 | 0.5625 | -0.70727 | 0.29464 |
| **22** | 0.31193 | 12 | 6 | 10 | 2 | 0.85714 | 0.6250 | -0.72987 | 0.23214 |
| **23** | 0.30578 | 13 | 6 | 10 | 1 | 0.92857 | 0.6250 | -0.74590 | 0.30357 |
| **24** | 0.30165 | 14 | 6 | 10 | 0 | 1.00000 | 0.6250 | -0.75679 | 0.37500 |
| **25** | 0.29348 | 14 | 5 | 11 | 0 | 1.00000 | 0.6875 | -0.77856 | 0.31250 |
| **26** | 0.28845 | 14 | 4 | 12 | 0 | 1.00000 | 0.7500 | -0.79214 | 0.25000 |
| **27** | 0.25937 | 14 | 3 | 13 | 0 | 1.00000 | 0.8125 | -0.87362 | 0.18750 |
| **28** | 0.25358 | 14 | 2 | 14 | 0 | 1.00000 | 0.8750 | -0.89052 | 0.12500 |
| **29** | 0.24469 | 14 | 1 | 15 | 0 | 1.00000 | 0.9375 | -0.91699 | 0.06250 |
| **30** | 0.23485 | 14 | 0 | 16 | 0 | 1.00000 | 1.0000 | -0.94706 | 0.00000 |

B. Sensitivity and specificity of CA-125 decrease after 2 cycles of chemotherapy for predicting TTP <6 months versus ≥ 6 months in relapsed patients excluding patients with recent ascitic drains (n=22).

| **Obs** | **_PROB_** | **_POS_** | **_NEG_** | **_FALPOS_** | **_FALNEG_** | **_SENSIT_** | **_1MSPEC_** | **cutpoint** | **j** |
| --- | --- | --- | --- | --- | --- | --- | --- | --- | --- |
| **1** | 0.99872 | 1 | 11 | 0 | 10 | 0.09091 | 0.00000 | 1.31467 | 0.09091 |
| **2** | 0.95596 | 2 | 11 | 0 | 9 | 0.18182 | 0.00000 | 0.35190 | 0.18182 |
| **3** | 0.94493 | 3 | 11 | 0 | 8 | 0.27273 | 0.00000 | 0.28863 | 0.27273 |
| **4** | 0.79541 | 4 | 11 | 0 | 7 | 0.36364 | 0.00000 | -0.11066 | 0.36364 |
| **5** | 0.77237 | 4 | 10 | 1 | 7 | 0.36364 | 0.09091 | -0.14728 | 0.27273 |
| **6** | 0.69517 | 5 | 10 | 1 | 6 | 0.45455 | 0.09091 | -0.25414 | 0.36364 |
| **7** | 0.65423 | 5 | 9 | 2 | 6 | 0.45455 | 0.18182 | -0.30435 | 0.27273 |
| **8** | 0.62685 | 6 | 9 | 2 | 5 | 0.54545 | 0.18182 | -0.33635 | 0.36364 |
| **9** | 0.59496 | 7 | 9 | 2 | 4 | 0.63636 | 0.18182 | -0.37245 | 0.45455 |
| **10** | 0.56570 | 7 | 8 | 3 | 4 | 0.63636 | 0.27273 | -0.40477 | 0.36364 |
| **11** | 0.48028 | 8 | 8 | 3 | 3 | 0.72727 | 0.27273 | -0.49709 | 0.45455 |
| **12** | 0.43156 | 9 | 8 | 3 | 2 | 0.81818 | 0.27273 | -0.54996 | 0.54545 |
| **13** | 0.39704 | 9 | 7 | 4 | 2 | 0.81818 | 0.36364 | -0.58824 | 0.45455 |
| **14** | 0.30176 | 9 | 6 | 5 | 2 | 0.81818 | 0.45455 | -0.70149 | 0.36364 |
| **15** | 0.28001 | 9 | 5 | 6 | 2 | 0.81818 | 0.54545 | -0.72987 | 0.27273 |
| **16** | 0.26815 | 10 | 5 | 6 | 1 | 0.90909 | 0.54545 | -0.74589 | 0.36364 |
| **17** | 0.26028 | 11 | 5 | 6 | 0 | 1.00000 | 0.54545 | -0.75678 | 0.45455 |
| **18** | 0.24500 | 11 | 4 | 7 | 0 | 1.00000 | 0.63636 | -0.77856 | 0.36364 |
| **19** | 0.23578 | 11 | 3 | 8 | 0 | 1.00000 | 0.72727 | -0.79213 | 0.27273 |
| **20** | 0.18560 | 11 | 2 | 9 | 0 | 1.00000 | 0.81818 | -0.87361 | 0.18182 |
| **21** | 0.16245 | 11 | 1 | 10 | 0 | 1.00000 | 0.90909 | -0.91698 | 0.09091 |
| **22** | 0.14780 | 11 | 0 | 11 | 0 | 1.00000 | 1.00000 | -0.94706 | 0.00000 |
