# Supplementary material for: Exploratory Analysis of TP53 Mutations in Circulating Tumour DNA as Biomarkers of Treatment Response for Patients with Relapsed High-Grade Serous Ovarian Carcinoma: A Retrospective Study
Source: PLoS Med. 2016 Dec 20;13(12):e1002198. doi: 10.1371/journal.pmed.1002198 (PMC5172526; doi:10.1371/journal.pmed.1002198)
Supplement: S17 Table — (DOCX) [file pmed.1002198.s027.docx]

**S17 Table. Sensitivity and specificity of decrease in TP53MAF and CA-125 for predicting 6 month TTP following one and two cycles of chemotherapy.**

| **Sensitivity and specificity of decrease in TP53MAF and CA-125 for predicting 6 month TTP** | | | | |
| --- | --- | --- | --- | --- |
| **TP53MAF** |  |  |  |  |
| **60% decrease from cycle 1 to 2** | **Sens**  **(95% CI)** | **Spec**  **(95% CI)** | **NPV**  **(95% CI)** | **PPV**  **(95% CI)** |
| All (n_courses_=31)* | 71% (42%–92%) | 88% (64%–99%) | 79% (54%–94%) | 83% (52%–98%) |
| Excluding Drains (n_courses_=24)* | 75% (43%–95%) | 100% (74%–100%) | 80% (52%–96%) | 100% (66%–100%) |
| **80% decrease from cycle 1 to 3** |  |  |  |  |
| All (n=30) | 86% (57%–98%) | 88% (62%–98%) | 88% (62%–98%) | 86% (57%–98%) |
| Exc. Drains (n=22) | 91% (59%–100%) | 100% (72%–100%) | 92% (62%-100%) | 100% (69%–100%) |
| **CA-125** |  |  |  |  |
| **50% decrease from cycle 1 to 2** |  |  |  |  |
| All (n_courses_=31)* | 93% (66%–100%) | 29% (10%–56%) | 83%(36%–100%) | 52% (31%–72%) |
| Excluding Drains (n_courses_=24)* | 92% (62%–100%) | 33% (9%–65%) | 80% (28%–99%) | 58% (34%–80%) |
| **50% decrease from cycle 1 to 3** |  |  |  |  |
| All (n_courses_=30) | 71% (42%–92%) | 63% (35%–85%) | 71% (42%–92%) | 63% (35%–85%) |
| Excluding Drains (n_courses_=22) | 73% (39%–94%) | 73% (39%–94%) | 73% (39%–94%) | 73% (39%–94%) |

*One course of chemotherapy was excluded from the sensitivity/specificity analysis for 6-month TTP since it was censored before 6 months.
