# Supplementary material for: Exploratory Analysis of TP53 Mutations in Circulating Tumour DNA as Biomarkers of Treatment Response for Patients with Relapsed High-Grade Serous Ovarian Carcinoma: A Retrospective Study
Source: PLoS Med. 2016 Dec 20;13(12):e1002198. doi: 10.1371/journal.pmed.1002198 (PMC5172526; doi:10.1371/journal.pmed.1002198)
Supplement: S1 Text — (DOCX) [file pmed.1002198.s028.docx]

**TP53 ctDNA in relapsed ovarian cancer: Statistical analysis plan outline**

**Statistical analysis plan September 2015**

**Primary Aim -** define the distribution and dynamics of ctDNA in patients with recurrent HGSOC treated with standard of care chemotherapy, and to correlate ctDNA with the volume of disease.

- Baseline descriptive statistics of 4 blood parameters of interest pre-treatment (TP53MAF, TP53MAC, TP53TAC, CA-125) and volume of disease in relapsed and newly diagnosed courses.
- Correlation of pre-treatment TP53MAF, TP53MAC, TP53TAC, CA-125 with each other at baseline [Pearson rank correlation].
- Correlation of TP53MAF, TP53MAC, TP53TAC and CA-125 with volume of disease in the total relapsed population, and according the presence or absence of ascites [Pearson rank correlation].
- Description of the kinetics of change of ctDNA compared with CA-125 after 1 cycle of chemotherapy.

**Secondary Aims -** to evaluate whether early change in ctDNA could predict TTP.

- Correlation of pre-treatment TP53MAF, TP53MAC, TP53TAC, CA-125 and volume with time to progression (TTP) [Cox regression model].
- Assessment of the value of change in TP53MAF, TP53MAC, TP53TAC, CA-125 after 1 cycle of chemotherapy in predicting TTP [Cox regression model].
- Assessment of the value of change in TP53MAF, TP53MAC, TP53TAC, CA-125 after 1 cycle of chemotherapy in predicting 6 month TTP: optimal cut-point for fall to be determined by ROC curves [logistic regression].
- Assessment of the value of change in TP53MAF, TP53MAC, TP53TAC, CA-125 after 2 cycles of chemotherapy in predicting TTP [Cox regression model].
- Assessment of the value of change in TP53MAF, TP53MAC, TP53TAC, CA-125 after 2 cycles of chemotherapy in predicting 6 month TTP: optimal cut-point for fall to be determined by ROC curves [logistic regression].

**Changes to initial analysis or additional non-planned analyses carried out following results from planned analysis**

- Assessment of the value of change in TP53MAF, TP53MAC, TP53TAC, CA-125 after 1 & 2 cycles of chemotherapy in predicting 6 month TTP with and without ascites. Cut-point for fall to be determined by ROC curves.
- Initially we did not specify the ‘evaluable’ criteria for ctDNA levels for response analysis. We followed CA-125 criteria for evaluability (double normal range) and used double the detectable ctDNA level as the criteria for evaluable courses for response analysis.

Extended analyses populations to maximise the information used as follows

- Courses assessable for volumetric correlation were initially restricted to those with a CT within 14 days of ctDNA. We noticed what appeared to be an interesting difference in TP53/MAF in the untreated and relapse population, and wished to maximise the number of courses available for analysis in this exploratory study. We therefore removed the 14 day restriction, increasing the number of relapse courses available for volumetric analysis from 40 to 51 (1/51 non-measurable disease on volumetric CT) and new courses from 2 to 7. [Figures 2b, 2e and f].
- We initially planned to exclude courses for response analysis with previous chemotherapy ≤ 28 days (the washout period for most clinical trials). However, in order to maximise the number of courses available for analysis, we removed this restriction (additional maximum n_courses_=2).
- Courses assessable for response analysis were initially restricted to those with a baseline CT scans ≤ 28 from the start of chemotherapy (in line with standard clinical trial exclusion criteria), however in order to maximise the number of courses available for analysis we removed this restriction (increasing the number courses from 30 to 32, Table 2).

**Additional analyses carried out and added to final manuscript through review process and editorial comments**

- Unit of TP53TMAF as a continuous variable in pre-treatment multivariate analysis changed from 100% to 1%.
- Unit of change in TMAF and CA-125 for multivariate analysis changed from a 100% to 1%.
- Confidence intervals added to sensitivity and specificity values as requested by the reviewers.
